# An emerging zoonotic clone in the Netherlands provides clues to virulence and zoonotic potential of *Streptococcus suis*

**DOI:** 10.1038/srep28984

**Published:** 2016-07-06

**Authors:** N. Willemse, K. J. Howell, L. A. Weinert, A. Heuvelink, Y. Pannekoek, J. A. Wagenaar, H. E. Smith, A. van der Ende, C. Schultsz

**Affiliations:** 1 Department of Medical Microbiology, Academic Medical Center, University of Amsterdam, 1105 AZ Amsterdam, The Netherlands; 2 Department of Global Health-Amsterdam Institute for Global Health and Development, Academic Medical Center, University of Amsterdam, 1105 BM Amsterdam, The Netherlands; 3 Department of Paediatrics, School of Clinical Medicine, University of Cambridge, Cambridge CB2 0QQ, United Kingdom; 4 Department of Veterinary Medicine, School of Biological Sciences, University of Cambridge, Cambridge CB3 0ES, United Kingdom; 5 GD Animal Health, 7400 AA Deventer, The Netherlands; 6 Department of Infectious Diseases and Immunology, Faculty of Veterinary Medicine, Utrecht University, 3584 CL Utrecht, The Netherlands; 7 Central Veterinary Institute part of Wageningen UR, 8221 RA Lelystad, The Netherlands; 8 National Reference Laboratory of Bacterial Meningitis, Academic Medical Center, University of Amsterdam, 1105 AZ Amsterdam, The Netherlands; 9 Oxford University Clinical Research Unit, Centre for Tropical Medicine, District 5, Ho Chi Minh City, Vietnam

## Abstract

*Streptococcus suis* is a zoonotic swine pathogen and a major public health concern in Asia, where it emerged as an important cause of bacterial meningitis in adults. While associated with food-borne transmission in Asia, zoonotic *S. suis* infections are mainly occupational hazards elsewhere. To identify genomic differences that can explain zoonotic potential, we compared whole genomes of 98 *S. suis* isolates from human patients and pigs with invasive disease in the Netherlands, and validated our observations with 18 complete and publicly available sequences. Zoonotic isolates have smaller genomes than non-zoonotic isolates, but contain more virulence factors. We identified a zoonotic *S. suis* clone that diverged from a non-zoonotic clone by means of gene loss, a capsule switch, and acquisition of a two-component signalling system in the late 19th century, when foreign pig breeds were introduced. Our results indicate that zoonotic potential of *S. suis* results from gene loss, recombination and horizontal gene transfer events.

*Streptococcus suis* (*S. suis*) is a zoonotic swine pathogen which is carried asymptomatically by up to 80% of healthy pigs^1^,^2^,^3^, and a major public health concern in China and South-East Asia^4^,^5^,^6^,^7^,^8^ causing severe disease in humans including meningitis and septicaemia. Outbreaks of human infection have occurred in China in 1998 and 2005^9^, a high incidence rate of meningitis and sepsis was reported from Thailand^7^, and *S. suis* infection is the leading cause of bacterial meningitis in adults in Vietnam and Hong Kong^10^. In North America and Europe *S. suis* is predominantly an occupational hazard^10^,^11^,^12^. *S. suis* is a Gram-positive bacterial pathogen of which 29 serotypes are known^13^,^14^,^15^,^16^. *S. suis* genotypes can be determined using multi locus sequence typing (MLST)^17^ and further clustered into clonal complexes (CC)^18^.

Human *S. suis* infection is predominantly caused by *S. suis* serotype 2 and to a lesser extent serotype 14^13^, but sporadic cases of human *S. suis* infections with serotypes 1, 4, 5, 9, 16, 21, 24 and 31 have also been described^19^,^20^,^21^,^22^,^23^,^24^,^25^. In contrast, the majority of porcine *S. suis* infections are caused by multiple serotypes including 2, 9, 3, 1/2 and 7^13^. Human serotype 2 isolates belong to a limited number of genotypes of which CC1 has spread worldwide^13^. Other genotypes appear restricted to certain geographical locations. For example, zoonotic isolates of *S. suis* belonging to CC20 have only been reported from the Netherlands. Whilst serotype 2 (CC1 and CC20) is the only *S. suis* serotype isolated from human patients in the Netherlands, the most prevalent serotype causing invasive disease in the Dutch pig population is serotype 9 (CC16)^11^. *S. suis* serotype 9 is carried by the vast majority of healthy pigs in Dutch pig herds. It is unknown why isolates with serotypes that are highly prevalent among diseased pigs, such as serotype 9, rarely cause human disease and which bacterial factors contribute to zoonotic potential.

A recent genomic comparison of porcine *S. suis* isolates from the United Kingdom and porcine and zoonotic isolates from Vietnam showed that isolates associated with porcine disease contained less genes than carriage isolates from healthy pigs, but did contain more virulence factors^26^. However, the isolate collection was not suitable to explore genomic differences that could explain zoonotic potential of *S. suis* because of the high similarity with respect to serotype and genotype, between the Vietnamese zoonotic isolates and the limited number of porcine isolates from Vietnam.

In this study, the differential distribution of serotypes and genotypes across zoonotic isolates and porcine invasive isolates, allowed us to conduct a genomic comparison of invasive *S. suis* isolates isolated in the Netherlands between 1982 and 2008, with the aim to identify genomic differences that could explain differences in zoonotic potential between *S. suis* isolates. We defined the population structure, described the recently emerged zoonotic *S. suis* CC20 isolates that diverged from the non-zoonotic CC16 isolates by means of a capsule switch and dated the divergence back to the late 19^th^ century, when foreign pig breeds were introduced and mixed. During its evolution, the zoonotic CC20 isolates acquired a pathogenicity island and a prophage containing a novel type I restriction modification system. Our findings shed new light on the emergence of a zoonotic *S. suis* clone as well as on genetic factors potentially contributing to virulence and zoonotic potential.

## Results

### Population structure of *S. suis* in the Netherlands

Whole genome sequencing of 98 isolates from the Netherlands isolated between 1982 and 2008 (Supplementary Fig. 1a) confirmed the previously reported serotypes (Supplementary Fig. 1b) and sequence types, as determined by MLST^17^ and represented by CC (Supplementary Fig. 1c), among invasive isolates from human patients and pigs. We assessed the population structure of all isolates by Bayesian Analysis of Population Structure (BAPS)^27^ and included 18 complete reference genomes from NCBI (Supplementary Table 1). BAPS was performed on the nucleotide alignment of the core genome and categorized the isolates into 7 groups (Supplementary Fig. 2) ranging from 1 to 44 isolates (Supplementary Fig. 3a). BAPS-based population grouping did not correlate with serotype as each serotype for which at least two isolates were present, grouped in at least two different BAPS groups (Supplementary Fig. 3b). However, the BAPS grouping correlated well with the CC of the isolates (Supplementary Fig. 3c). BAPS group 1 comprised all CC13 isolates, BAPS group 2 comprised most of the CC16 isolates, BAPS group 4 comprised all CC1 isolates, BAPS group 5 comprised both the CC27 and the CC29 isolates, and BAPS group 6 comprised most CC20 isolates. BAPS group 3 consisted solely of the isolate 9401240, a MLST singleton sequence type not belonging to any clonal complex, and BAPS group 7 comprised mostly diverse unrelated isolates as well as 3 CC16 isolates. Human isolates were found in BAPS groups 4 and 6 (Supplementary Fig. 3d), where BAPS group 4 included the CC1 isolates and BAPS group 6 the CC20 isolates and we postulate that isolates within these two populations are capable of causing human infection (i.e. having zoonotic-potential), whilst isolates outside of these groups are far less capable of causing human infection (termed non-zoonotic).

### Zoonotic isolates have low gene content with a high number of virulence factors

**Figure 1:**
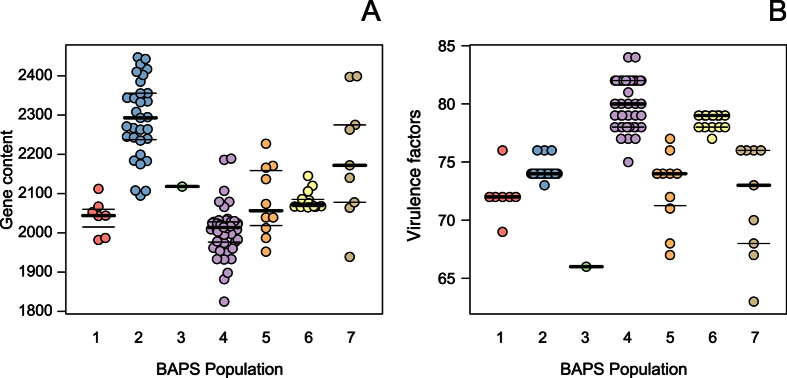
Gene content and virulence factor distribution of the isolates divided by BAPS grouping. (**A**) Dot plot representing the gene content separated by BAPS group. Significant differences are 1 vs 2, p < 0.01; 2 vs 4, p < 0.001; 2 vs 5, p < 0.01; 2 vs 6, p < 0.05; 4 vs 6, p < 0.05 and 4 vs 7, p < 0.01. (**B**) Dot plot representing the number of virulence factors separated by BAPS group. Significant differences are 1 vs 4, p < 0.001; 1 vs 6, p < 0.01; 2 vs 4, p < 0.001; 2 vs 6, p < 0.01; 4 vs 5, p < 0.001; 5 vs 6, p < 0.01; 4 vs 7, p < 0.001 and 6 vs 7, p < 0.01. Statistical significance was determined using the Kruskal Wallis test followed by Dunn’s post-test. Stars were omitted from the graph for clarity. The thick black lines indicate the median and the thinner black lines represent the first and third quartile.

**Figure 2:**
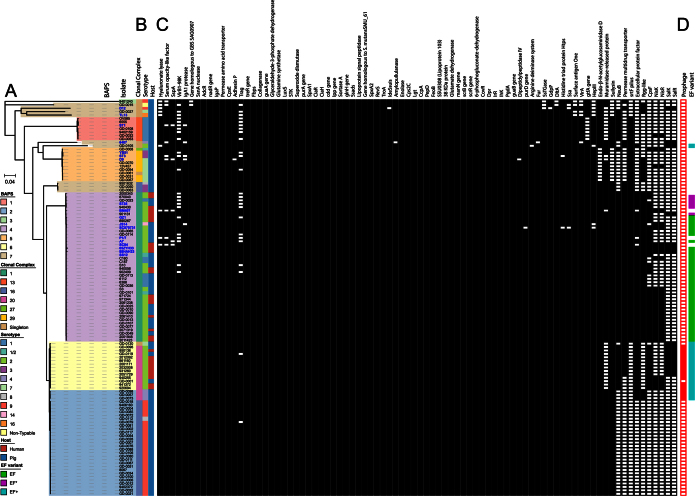
Characteristics and virulence gene content for each isolate in relation to a maximum likelihood phylogenetic tree. (**A**) Maximum likelihood tree of *S. suis* based on 55464 non-recombinant core genome SNPs as determined by Gubbins. The phylogenetic tree included all 116 *S. suis* isolates from this study and BAPS population grouping is overlaid on the branches. The tree illustrates how the isolates from BAPS groups 2 and 6 cluster together on a branch that only consists of Dutch isolates and none of the reference isolates. BAPS group 3, consisting solely of the MLST singleton isolate 9401240, is the most outlying isolate. (**B**) Characteristics of isolates in relation to population group. The serotype, clonal complex and host species from which the isolate was obtained are indicated with colored strips. (**C**) The presence and absence matrix of each of 84 virulence factors for each isolate. Presence is indicated with a black box, and absence with a white box. The name or description of each virulence factor is indicated above the matrix and further information for each factor is provided in Supplementary Table 2. (**D**) Presence and absence of the 18.5 kb prophage region encoding a type I restriction modification system and the extracellular protein variants. Presence of the prophage is indicated with a solid red box, and absence is indicated with a white box. The composition of the prophage is illustrated in Supplementary Fig. 13. Presence of a variant of the extracellular protein for each of the isolates. Green: EF, magenta: EF* and Cyan: EF+.

The gene content of isolates in our complete data set ranged from 1825–2447 genes. Zoonotic BAPS group 4 isolates had a significantly lower gene content with a median of 2014 genes compared to the gene content of BAPS groups 2 (p < 0.001), 6 (p < 0.05) and 7 (p < 0.01) (Fig. 1A). The highest gene content with a median content of 2293 was found among isolates belonging to non-zoonotic BAPS group 2, which was significantly higher than the gene content of BAPS groups 1 (p < 0.01), 4 (p < 0.001), 5 (p < 0.01) and 6 (p < 0.05). In contrast, zoonotic BAPS groups 4 and 6 isolates possessed significantly more putative virulence factors than BAPS groups 1, 2, 5 and 7 (all p < 0.01) isolates (Fig. 1B). Overall the presence of virulence genes was high across all isolates considering that the observed lowest number of virulence factors in a single isolate was 63 (Fig. 2C). Indeed, 48 out of the 84 putative virulence factors that were analyzed (Supplementary Table 2), were part of the core genome. A discriminant analysis of the presence or absence of the remaining 36 non-core virulence factors between the zoonotic BAPS groups (4 and 6) and the non-zoonotic BAPS groups (1, 2, 3, 5 and 7) indicated a clear separation (Supplementary Fig. 4a). Genes that contributed most to this separation encoded for 7 virulence factors, including a capsular polysaccharide biosynthesis gene (*cps2F*)^28^, an N-acetylneuraminic acid synthetase (*neuB*)^29^, an extracellular protein factor (*epf*)^30^, an Rgg-like transcriptional regulator^31^, an ABC-type multidrug transport system^32^, suilysin (*sly*)^33^ and endo-β-N-acetylglucosaminidase D^34^ (Supplementary Fig. 4b). Both BAPS groups 4 and 6 consisted of all but one of the serotype 2 isolates and this explained why *cps2F* and *neuB*, which are part of the serotype 2 capsule locus, showed up in this analysis, confirming the association of serotype 2 isolates with zoonotic infection. We previously reported negative PCRs for detection of *epf* in CC20 isolates, but here we show clearly that a variant of the *epf* gene is present in 55 out of 58 zoonotic isolates and in only 4 out of 58 non-zoonotic isolates. CC20 isolates possess a longer EF protein of 1492 amino acids, similar to EF^* ^30, but with 85% identity at amino acid level including a nearly identical N-terminal region of 835 amino acids (Supplementary Fig. 5). We designated this protein EF^+^. Interestingly, the three CC20 isolates of serotype 4 had a C-terminally truncated variant of the CC20 EF^+^ protein of 1054 amino acids in length (Fig. 2D).

### Divergence of zoonotic BAPS group 6 isolates and non-zoonotic BAPS group 2 isolates

Phylogeny was assessed between BAPS groups by constructing a phylogenetic tree using the non-recombinant single nucleotide polymorphisms (SNPs) from the core genomes, as determined with Gubbins^35^ (Fig. 2A). Isolate 9401240 from BAPS group 3 was an outlier in this tree, but we still considered it to be *S. suis*, as it contained all the housekeeping genes and including isolate 9401240 did not drastically reduce the core genome. The zoonotic BAPS groups 4 and 6 isolates clustered on different branches of the tree with the zoonotic BAPS group 6 isolates clustering on the same branch as the non-zoonotic BAPS group 2 isolates (Fig. 2B). Interestingly, none of the reference sequences clustered in the branch of BAPS groups 2 and 6 suggesting that these isolates are geographically restricted, but comparison with additional recent isolates from other geographic regions would be necessary for confirmation.

**Figure 3:**
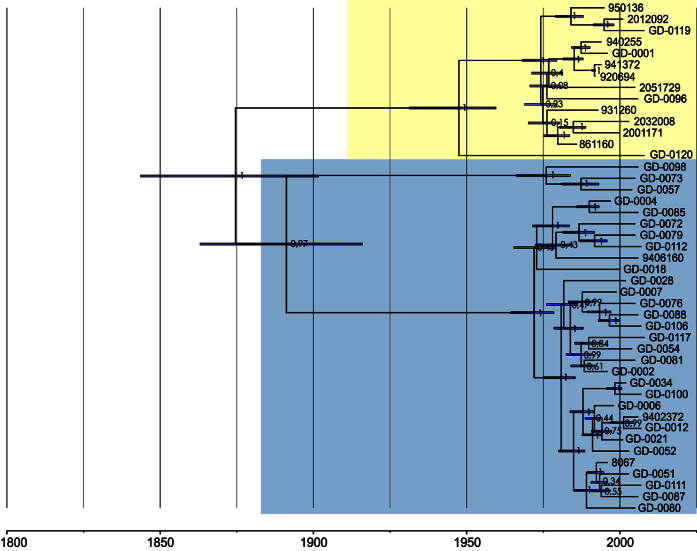
Dated phylogeny of BAPS groups 2 and 6 of *S. suis*. The phylogenetic tree generated using the core genome of 45 isolates belonging to BAPS groups 2 and 6. BAPS grouping is overlaid over the branches where blue indicates BAPS group 2 and yellow BAPS group 6. Node labels indicate the posterior and the node bars indicate the 95% highest posterior density. The dates in years are shown on the x-axis. Major divergence events, coinciding with capsule switches, can be seen around 1875 and 1891.

The temporal distribution of isolates allowed us to determine the origin of divergence between BAPS groups 2 and 6. The non-recombinant SNPs from the core genome from BAPS groups 2 and 6 isolates were analyzed with Bayesian Evolutionary Analysis Sampling Trees (BEAST)^36^. Divergence of BAPS group 2 and 6 occurred in the second half of the 19th century around 1875 (95% posterior of 1844–1902) followed by a second separation around 1891 (95% posterior of 1863–1916) which gave rise to a subset of 3 isolates (Fig. 3). Both divisions were accompanied by a capsule switch, as the first event separated serotype 9 and serotype 2 isolates and the second event separated serotype 9 and serotype 4 isolates. We extracted the serotype 2 capsule locus sequences from the isolates in BAPS groups 4 and 6 and found a higher similarity between these loci compared to the similarity between core genomes of these isolates (Supplementary Fig. 6).

### Zoonotic potential is determined by the accessory genome

We assessed the accessory genome by Principal Component Analysis (PCA), separating isolates based on the presence or absence of accessory genes^37^, overlaid with metadata including BAPS group, host, clonal complex and serotype (Supplementary Fig. 7a–d). In contrast to the results of BAPS on SNPs in the core genome, PCA on genes in the accessory genome separated isolates from BAPS group 2 and 6 and clustered group 6 isolates closer to group 4 isolates. These results indicate not only that BAPS groups 2 and 6 isolates were more diverse in their accessory genome than in their core genome, but also that zoonotic BAPS groups 4 and 6 were more similar in their accessory genome than in their core genome. We constructed a Hierarchical Clustering of Principal Components (HCPC) of the PCA outcomes (Supplementary Fig. 8). Cluster 1 consisted of two Chinese outbreak isolates, 05ZYH33 and 98HAH33, which are outliers in comparison to other CC1 isolates, including the Chinese outbreak strain SC84, consistent with sequence discrepancies between these outbreak isolates reported earlier^26^,^38^. Cluster 6 contained all CC16 isolates and had the largest and most divergent accessory genome. The zoonotic CC1 and CC20 isolates showed similarity in their accessory genome as they are grouped together in cluster 4 when a cut-off of 6 clusters was chosen (Supplementary Fig. 8a,c), which is in contrast with BAPS which indicated that CC20 isolates are similar to CC16 isolates in their core genome. CC1 and CC20 isolates were only separated into clusters 3 and 6 when a cut-off of 8 clusters was chosen (Supplementary Fig 8b,d). A heat map was plotted to illustrate the shared accessory gene content (Supplementary Fig. 9). The heat map demonstrated that isolates belonging to BAPS group 2 had the lowest percentages of their accessory genome present in other isolates, which is expected as BAPS group 2 has the largest accessory genome. The heat map also demonstrates that the BAPS group 6 isolates shared more homologous groups with the accessory genome of BAPS group 4 isolates than with BAPS group 2 isolates.

**Figure 4:**
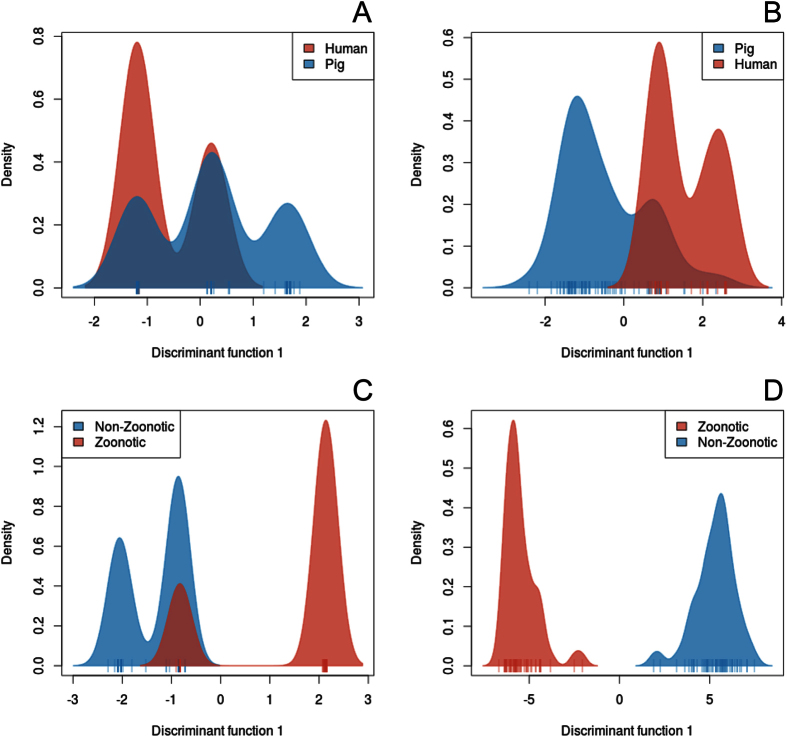
Discriminant analysis of principle components on the core and accessory genome, which illustrates the gene content differences in the accessory genome and SNP differences in the core genome. DAPC plots were performed on the core genome (**A,C**) and on the accessory genome (**B,D**). The analyses were performed using two types of prior grouping: Host (**A,B**) and zoonotic potential (**C,D**). Density plots are displayed as only one linear discriminant is available when comparing between two groups.

We then performed a Discriminant Analysis of Principle Components (DAPC)^39^,^40^ of core genome SNPs and accessory genes to determine discriminating variables for groups of isolates. Isolates were grouped by host and by zoonotic potential and for the latter BAPS groups 4 and 6 isolates were labelled as ‘zoonotic’ and isolates in the remaining BAPS groups as non-zoonotic. At host level, analysis of both core (Fig. 4A) and accessory (Fig. 4B) genomes revealed three distinct peaks for the pig isolates of which two overlapped with peaks from the human isolates indicating some isolates were restricted to the porcine host, which could be traced back to CC16 isolates. The two human host peaks suggested there were two groups of isolates with zoonotic potential, which correlated with the CC1 and CC20 isolates. Zoonotic and non-zoonotic groups separated better on the accessory genome (Fig. 4D) than on the core genome (Fig. 4C). Therefore, DAPC analysis of the accessory genome was consistent with PCA and HCPC analyses, whilst DAPC of the core genome corroborated the BAPS results.

### Unique components of the zoonotic CC20 isolates

**Figure 5:**
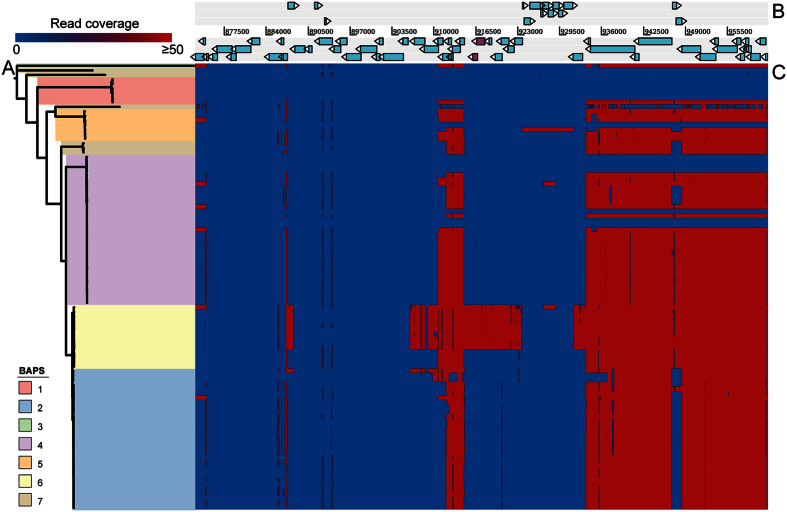
Mapping plot representing the presence of the 89k pathogenicity region in the sequenced isolates. (**A**) Maximum Likelihood tree, created by Gubbins, on the core genome using only the sequenced isolates. Branch lengths were ignored for clarity. (**B**) Annotated pathogenicity island extracted from isolate 05ZYH33 and visualized by Artemis. The *salK*/*salR* two component signaling system is indicated by the magenta coding sequences. (**C**) Heat map demonstrating the presence/absence of the pathogenicity island. The reads from the sequenced isolates were mapped against the 89k pathogenicity island from isolate 05ZYH33 and a maximum read coverage was set at 50 to demonstrate a presence by red and an absence by blue. Each row describes the presence/absence for one isolate. Only CC20 isolates contained the *salK*/*salR* system and the synteny of the pathogenicity island was checked in the assemblies of the CC20 isolates.

A two component signaling system (TCSS) *salK*/*salR* was present in 9 out of 13 zoonotic CC20 isolates. This TCSS was previously found on a pathogenicity island (PAI) that has been linked to high virulence^41^ of Chinese outbreak isolates. So far this TCSS has not been found in European isolates, although a recent study identified divergent *salK*/*salR* genes in respiratory and non-clinical serotype 4 isolates from the United Kingdom^26^. We mapped reads from our dataset (Fig. 5) and from the isolates described by Weinert *et al*. (Supplementary Fig. 10) to the 89 kb pathogenicity island from isolates 05ZYH33 and SC84 and found that the reads of the UK isolates did not map to the TCSS, due to their low identity (55.78%) to *salK*/*salR,* whilst the reads of the CC20 isolates mapped with high identity (97.99%). We determined the insertion location and the complete sequence of the island from isolate 931260, which contained the entire island on a single contig (Supplementary Fig. 11 and Supplementary Note 1). The island was inserted into the SSU0561 (a 23S rRNA uracil-5–methyltransferase RumA) homologue from isolate P1/7 and was located several genes downstream of the capsule locus. The island in 931260 was 65988 nucleotides in length with a GC content of 35.6% (Supplementary Fig. 12) and contained fragments of the 89K pathogenicity island in synteny, which suggests that the pathogenicity island was shared between the Chinese CC1 zoonotic outbreak strains and Dutch CC20 zoonotic endemic strains.

Finally, exploratory DAPC analysis yielded an 18.5 kb prohage region which was unique to the CC20 isolates and only one porcine CC20 isolate (GD-0001) lacked this prophage (Fig. 2D). The prophage region appeared inserted after an integrase with high identity (99%) to a gene in *S. suis* isolates YS12 (WP_024405908) and 89-3576-3 (WP_024395351), and in front of two ribosomal proteins rpsI (30S ribosomal protein S9) and rplM (50S ribosomal protein L13). A PHAge Search Tool (PHAST)^42^ analysis resulted in zero hits but a BLAST search against the Genbank database indicated that *S. suis* isolate YS12, which was isolated from a healthy pig in China, contained the prophage region as well. YS12 is a serotype 7 isolate belonging to sequence type 17, which is part of CC20^43^. In contrast, the Dutch sequence type 17 isolates, expressed serotype 4. None of the 375 isolates described by Weinert *et al*. contained this prophage region. The prophage contained typical phage genes including integrases and Tn*5252* transposons, but more interestingly also contained a complete type I restriction-modification (R-M) system, consisting of a restriction and a modification subunit as well as several specificity subunits (Supplementary Fig. 13), with low identities to known type I R-M systems in the restriction enzyme database REBASE^44^. Whilst we do not know if these genes translate into a functional R-M protein complex, it appears a prophage introduced a novel type I R-M system into the CC20 lineage.

## Discussion

In this study, we performed for the first time whole genome sequence analyses on a collection of invasive *S. suis* isolates from human patients and from pigs with invasive disease, from the same geographical origin and time frame, to assess determinants of zoonotic potential. Zoonotic potential of *S. suis* was associated with smaller gene contents. Recently it has been suggested that increased bacterial virulence is not only associated with the presence of virulence factors, but also with a reduction in gene content^45^,^46^. Large genomes may contain anti-virulence genes that could inhibit a potentially pathogenic phenotype and such phenotype may also be important for zoonotic potential. Our principal component analyses indicated that zoonotic potential of CC1 and CC20 isolates is associated with presence or absence of accessory genes. Zoonotic potential of *S. suis* was also associated with a high number of virulence factors, in particular the capsule related genes *cps2F* and *neuB*, present in the serotype 2 capsule locus, as well as the extracellular protein factor *epf*. The EF protein has been associated with virulence^47^ whilst variant proteins which contain repeats, designated as EF*, were associated with lower virulence^30^. Now, in addition, we identified another EF variant in the CC20 isolates, designated EF^+^. We corroborated the epidemiologically established link between zoonotic potential and serotype, in agreement with the previously experimentally established correlation between serotype and host-pathogen interaction of *S. suis*^48^. In addition, we not only found support for the previously reported association of the *epf* gene with virulence but also linked the presence of the *epf* gene or variant gene to zoonotic potential^47^.

A phylogenetic tree of the core genome indicated high similarity between the zoonotic CC20 and non-zoonotic CC16 isolates and we determined that these clonal complexes diverged in the late 19th century, when foreign pig breeds were imported in the Netherlands and crossed with local breeds (http://encyclopedievanzeeland.nl/Varkens, http://www.encyclopediedrenthe.nl/Varken/varkenshouderij) (Translation in Supplementary Note 2). We postulate that the imported pigs carried *S. suis* which recombined with *S. suis* carried in the indigenous pig population, resulting in novel *S. suis* variants. Whilst we do not know the serotype of the common ancestor to the CC20 and CC16 isolates, we do see high similarity between the serotype 2 capsule loci of CC1 and CC20 isolates. This contrasts with the variation between the core genomes of CC1 and CC20 isolates, suggesting either high conservation of the capsule locus or separate evolution of clonal complexes with recent or frequent capsule switches between the zoonotic clones. Additional diversification occurred within CCs in more recent times, similar to observations reported in the UK^26^, but this expansion appears unlikely to have resulted in increased zoonotic potential.

For this study, human carriage *S. suis* isolates were not available for comparison, but it should be noted that evidence for persistent carriage of *S. suis* in humans is lacking. Previous studies have suggested that the human pharynx of slaughterhouse workers can be colonised with *S. suis*^49^,^50^. However, this finding is insufficient to support persistent healthy carriage. As *S. suis* has been shown to survive in the environment and to reside in bioaerosols, slaughterhouse workers are continuously or frequently exposed to pigs and pork, increasing the likelihood of frequent re-colonization with *S. suis*, as opposed to persistent carriage. In addition, molecular epidemiological data indicative of persistent carriage of *S. suis* in humans are lacking. A case-control study found only one out of 101 patients in whom sequential nasal swabs were found positive^8^, whilst sequential nasal and rectal samples from 300 community controls and 920 household members of cases, hospital controls and community controls were all found to be negative.

Finally, we analysed the recently diverged zoonotic CC20 isolates and found they contained a novel type I R-M system and fragments of a pathogenicity island previously only found in Chinese and South East Asian isolates. The prophage with this type I RM system was located behind an integrase also found in seemingly unrelated *S. suis* isolates YS12, 89-3576-3, and in our CC16 and CC20 isolates, and in front of two ribosomal proteins which are conserved in genomes of the *Streptococcus* genus. YS12 was found to contain the prophage region as well, whilst 89-3576-3 did not. R-M systems protect bacteria against invasion of foreign DNA by endonucleolytic cleavage of DNA that lacks a specific modification. Acquisition of a type I R-M system may explain why CC20 isolates kept small genomes. The lack of this protection in CC16 isolates may have facilitated the acquisition of additional genes. In addition, a type I R-M system may contribute to virulence. *Streptococcus pneumoniae* has recently been shown to possess a variety of R-M systems^51^, including a type I R-M system which contributes to virulence by switching specificity subunits^52^, thereby altering the methylation of the bacterial genome. Variation in methylation resulted in altered gene expression profiles, resulting in modified virulence of *S. pneumoniae*^52^. The type I R-M system present in the CC20 prophage also contained multiple S-subunits and switching between these S-subunits potentially could affect the virulence of the CC20 isolates. This observation is subject for future research. The high number of virulence factors found across our *S. suis* isolates may suggest different means of gene expression which may explain differences in virulence. The s*alK*/*salR* TCSS was previously only found in isolates from South-East Asia, located on a 89K PAI, as well as a variant which was found in 6 UK isolates^26^. The putative amino acid sequence of SalK in our CC20 isolates is 97.99% identical to those of the Chinese isolates. The *salK/salR* TCSS was found in what appears to be a 65 kb pathogenicity island in Dutch CC20 isolates. The similarities between the pathogenicity islands in the CC20 isolates and the CC1 isolates from China suggest horizontal gene transfer between Asian and Dutch *S. suis* isolates or transfer from a common source, potentially the result of early mixing of pig breeds. In conclusion, our results indicate that zoonotic potential of *S. suis* results from gene loss, recombination and horizontal gene transfer events. Our findings are important for understanding the emergence of zoonotic bacterial pathogens in general and *S. suis* in particular.

## Methods

### Bacterial isolates

98 isolates causing invasive disease were selected from the collection of Dutch isolates in the Academic Medical Center^11^. The selection included all 24 available human isolates cultured from cerebrospinal fluid or blood from patients with bacterial meningitis, and a wide variety of pig isolates. One pig isolate of every sequence type was selected and when more pig isolates of a sequence type were available, additional isolates were chosen randomly up to a total of 98 isolates. The anonymized isolates obtained from patients were received from the National Reference Laboratory of Bacterial Meningitis (NLRBM) at the Academic Medical Center of the University of Amsterdam and were isolated between 1986–2007. Isolates from pigs were obtained from the GD Animal Health, Deventer or the Veterinary Microbiological Diagnostic Centre of the Faculty of Veterinary Medicine, Utrecht. A representative sample of each year from a total number of 2773 non-duplicate invasive *S. suis* isolates collected in the period 1996–2008, was obtained by choosing every 20th isolate irrespective of source or serotype. *S. suis* isolates obtained from pigs were defined as invasive as they were cultured from brain tissue, cerebrospinal fluid, blood, or joints from pigs with clinical disease compatible with *S. suis* infection. Serotyping was performed using PCR for detection of serotypes 1 (and 14), 1/2 and 2, 7, and 9, including positive and negative control isolates^53^, and by slide agglutination using serotype specific antibodies for isolates which were untypeable by PCR. Multi Locus Sequence Typing (MLST) was performed as previously described^17^. Eighteen complete publicly available (as of October 1^st^, 2013) *S. suis* genomes were also included in our dataset for a total of 116 isolates. These reference sequences originated from China (14 isolates), UK (1 isolate), Denmark (1 isolate) Vietnam (1 isolate) and the Netherlands (1 isolate) and included 4 patient isolates from China, 1 patient isolate from Vietnam and 2 Chinese isolates from healthy pigs. For the majority of the reference sequences we were unable to determine the year of isolation, but the dates that were recovered fell within the same temporal distribution as the Dutch sequenced isolates. Clonal complexes were identified by grouping the isolates with all isolates present in the *S. suis* database and using the eBURST algorithm^18^. A table of isolates included in this study can be found in Supplementary Table 1. The assemblies of 375 UK and Vietnamese isolates that were used to put our results in a larger context were sequenced and described by Weinert *et al*.^26^.

### Genome sequencing

The bacteria were grown from glycerol stock overnight at 37 °C in Todd Hewitt broth with 0.2% yeast extract. Genomic DNA was isolated using the Wizard genomic DNA purification kit (Promega) according to protocol and the DNA was run on gel as well as measured using Qubit fluormetric quantification (Life Technologies). Four μg of genomic DNA was used as input for multiplex libraries which were created with an in-house library preparation pipeline using Life Technologies enzymes. Twenty isolates were sequenced per lane on an Illumina MiSeq sequencing platform using the 150 cycles paired end protocol for a total of 5 runs. FastQC software was used to inspect quality of the raw MiSeq reads^54^. Sequencing was repeated when the reads failed the quality checks.

### *De novo* genome assembly

A custom Perl script was used to perform post sequencing quality filtering. The cutadapt 1.4.2 software was used to remove the Illumina adapters introduced in the sequence reads during library preparation^55^. Next, undetermined nucleotides (Ns) were removed from the reads. Lastly, the program Sickle was used to trim the low quality nucleotides (Phred quality score <20) at the end of the sequence reads to a minimum length of 31 bp^56^. The trimmed fastq files were assembled with the program SPAdes 3.0.0^57^ using the manual’s default parameters for assembly of paired end 150 bp Illumina reads. Contigs shorter than 300 bp and/or low sequencing depth (<10) were removed from the assemblies as these are often the by-product of SPAdes assemblies. Assembly statistics were viewed to check for errors and 2 isolates were removed as their genome size was over 3,000,000 bp and N50 was <10,000 bp. The genomes from the remaining 98 isolates ranged in size from 1,969,756-2,341,754 bp with a GC content range of 40.95–41.41%, which is typical for *S. suis*^38^. Additional assembly statistics can be found in Supplementary Table 3. Briefly, the Illumina reads were mapped back to the scaffolds using the Burrows-Wheeler Aligner (BWA) software to check for the presence of any major assembly errors^58^. The bam files were visualized using the Artemis program^59^.

### Genome annotation

The prokaryotic genome annotation pipeline Prokka 1.9 was used for automatic annotation of the draft genomes^60^. The rfam parameter was turned on to enable searching for ncRNAs. Descriptive analysis of the gene content of *S. suis* was performed with the statistical program R^61^ and used the Prokka output. Statistical significance between gene contents was determined using the Kruskal Wallis test followed by Dunn’s post-test.

### Identification of orthologous groups

For identification of the pan-genome of *S. suis* we used OrthoMCL to determine the homology groups. The OrthoMCL pipeline was followed according to protocol^62^. The CDS determined by Prokka (which invokes Prodigal^63^) were used as input for an all vs all BLASTP with a BLAST e-value cut-off of 1e-5. A series of Perl scripts was then invoked to load the BLAST output into a MySQL database. The MCL algorithm with varying inflation values was used to cluster CDS in homology groups and we decided on an inflation value of 2.1 in order to maximize the core genome while minimizing the amount of false positives^64^. The output of the OrthoMCL pipeline consisted of 3964 homology groups. Custom scripts were written to translate the identifiers of the homology groups back to protein and nucleotide fasta format. Manual checks were performed on the homology groups to take into account sequencing errors and pseudogenes due to our draft genomes, but criteria were composed in order to select the homolog groups that needed manual inspection. The checks included: 1) Groups containing slightly more or fewer than 116 members, 2) Groups containing isolates with <60% amino acid identity, 3) Groups containing isolates with <80% of the modal gene length of the group. 4) Groups with a high F-statistic >0.6 (calculated using R package seqinr^65^) and <80% alignment identity (calculated using alistat which is part of the HMMER software package^66^) within a group. 5) Pseudogenes were identified as genes that have either <80% or >120% of the modal length of that group (many proteins truncated <10% from either end are still functional and are not pseudogenes^67^). All vs all BLAST searches were performed on each homolog group and clustal omega 1.2.0 was used to align the members of each group^68^. The seaview 4.5.2 program was used to visualize the alignments^69^. The PhyML 3.0 software was used to construct guide trees for the homology groups which were identified by the abovementioned checks^70^. The phylogenetic trees were viewed using FigTree 1.4.2 software^71^. The selected groups were checked manually and splits when necessary. The final number of homology groups after curation was 4028.

### Construction of the core and pan genome

The 4028 homology groups created using the OrthoMCL pipeline were designated the pan genome. 850 homolog groups contained a gene from every isolate and these were considered the core genome. However, after recalculating the core genome leaving one isolate out at the time, the core genome increased significantly when leaving either isolate 05ZYH33 or 98HAH33 out. Leaving both isolates out increased the core genome to 1015 homology groups. The core genome alignment of 850 genes was 805775 nucleotides long and contained 131376 SNPs. The core genome alignment of 1015 genes was 963857 nucleotides long and contained 148365 SNPs. The pan genome consisted of 4028 genes, which meant that the accessory genome consisted of 3013 genes. The core genome of 850 genes was used for population structure analysis and phylogeny, but other analyses used the core genome of 1015 genes. A custom bash script was used to create a nucleotide core genome alignment by concatenating all aligned homology groups of the core genome based on the synteny of isolate P1/7^38^. A presence/absence matrix of the pangenome was constructed using a custom R script^61^.

### Bacterial population structure

Bayesian Analysis of Population Structure (BAPS) software can be used to determine population structure of bacteria using nested clustering^72^ and in particular, hierBAPS 6.0 was used^27^. Clustering was performed with 3 levels in the hierarchy and a prior upper boundary of 50 clusters on the core genome alignment of 850 genes. The estimated number of clusters were 7, 23 and 36 for the levels 1, 2 and 3 respectively. The shuffled SNPs alignment output of hierBAPS is presented in Supplementary Fig. 2. BAPS includes every sequence position which contains at least two alleles. If there is only a single allele and an indel, the site is excluded from the analysis.

### Core genome phylogeny

To determine the phylogenetic relationships between the isolates we identified non-recombinant SNPs via the program Gubbins^35^. Gubbins is an algorithm that iteratively identifies loci containing elevated levels of base substitutions and constructs a phylogeny based on the SNPs outside these regions. The proposed regions of recombination are indicated in Supplementary Fig. 14. Gubbins called upon RAxML to build the phylogenetic tree^73^. The nucleotide core genome alignment of 850 genes was used as input and Gubbins was run for 5 iterations. The phylogenetic tree was visualized using the interactive Tree Of Life (iTOL) webtool^74^. To corroborate the ML tree generated by Gubbins we also used the full 805775 nucleotide alignment of the 850 genes core genome in ExaML to generate an Extended Majority Rule consensus tree (Supplementary Fig. 15), Briefly, we created bootstrap replicated using RAxML of the core genome alignment and generated parsimony starting trees, which were determined to be all unique. ExaML was run on each bootstrap replicate and its starting parsimony tree. RAxML was used to determine when sufficient bootstrap trees were generated and the bootstopping criterion was reached, which required 400 bootstrap replicates. An Extended Majority Rule consensus tree was finally generated using RAxML.

### Capsule locus phylogeny

The capsule gene locus was extracted from all serotype 2 isolates (N = 49). The capsule locus was selected from the 5′-side up and until the N-acetylneuraminic acid (*neuAC*) gene cluster as indicated in Okura *et al*.^75^. The sequences ranged in length from 22049 to 23722 nucleotides with a median length of 22172 and were aligned in Seaview. A maximum likelihood tree was generated using the aligned capsule locus sequences with the GTR model in PhyML, which was optimized for invariable sites and across site rate variation. We ran the algorithm for 100 bootstraps and visualised the most likely tree using FigTree 1.4.2.

### Principle component analysis

A principal component analysis (PCA) was performed on the accessory genome using the PCA function of the FactoMineR package^37^. A PCA is an exploratory analysis that can describe a dataset by reducing the variance to a few dimensions that can be plotted. These new linear dimensions are the principal components. We used the PCA to explore the accessory genome of our isolates based on the presence/absence of genes. The data points in the plotted dimensions were overlaid with metadata including MLST clonal complex, serotype, BAPS population and host (Supplementary Fig. 7). To show the hierarchy relation between the isolates in the PCA, a Hierarchical Clustering of Principal Components (HCPC), which can also be found in the FactoMineR package^37^, was used. HCPC suggests an optimal number of clusters based on inertia gain. The optimal number for our dataset was determined to be either 6 or 8 (Supplementary Fig. 8).

### Discriminant Analysis of Principal Components

A Discriminant Analysis of Principle Components (DAPC)^39^ is part of the adegenet^40^ package and adds a discriminant analysis to the PCA as it aims to maximize the difference between prior groups. The HCPC analysis showed that cluster 1, consisting of isolates 05ZYH33 and 98HAH33, were divergent in their accessory genome from other CC1 isolates. Furthermore, including these outbreak isolates in establishing the core genome also decreased the core genome drastically and it was decided to perform the DAPC without the Chinese outbreak isolates 05ZYH33 and 98HAH33. DAPC can be performed both on the SNPS in the core genome as well the presence and absence of genes in the accessory genome. The prior groupings supplied to the analyses consisted of host and zoonotic potential. In an effort to determine the difference between non-zoonotic and zoonotic isolates we labelled the zoonotic BAPS populations 4 and 6 as ‘zoonotic’ and the remaining BAPS populations a ‘non-zoonotic’. We kept the principal components during the DAPC analysis that explained 80% of the total variance and all discriminant functions.

### Molecular dated phylogeny

To determine the origin of the separation between CC16 and CC20 isolates a subset of the 1015 core genome genes alignment was selected from the non-recombinant SNPs that were determined by Gubbins. We used the Bayesian Evolutionary Analysis Sampling Trees (BEAST 1.8.1) software^36^ to produce a dated phylogeny. The software Path-O-Gen 1.4^76^ was run on the non-recombinant SNPs and a maximum likelihood tree was generated by Gubbins to confirm a molecular signal in the data (Supplementary Fig. 16). We ran BEAST with combinations of molecular clock models (strict or uncorrelated lognormal), substitution models (HKY85 and GTR) and a rate estimated from the dated tip model with a gamma distribution. A constant growth tree prior model, an exponential growth tree prior model and an extended Bayesian skyline plot prior tree model were compared by calculating the Bayes Factor from the harmonic mean of the likelihood with 10,000 bootstraps. All model combinations were run with a MCMC chain of 10,000,000 until burn-in represented less than 10%. A strict molecular clock model with a GTR substitution model and a constant growth tree prior model had the best fit. Convergence of the parameters and the Effective Sample Size (ESS) scores were checked using the program Tracer 1.4^77^. The dated phylogeny was visualized using FigTree 1.4.2.

### Sequence distribution heat map

Reads were mapped to a reference genomic region using BWA in order to visualize the presence and absence of accessory sequences in individual isolates. Coverages for every nucleotide were extracted from the bamfiles using Samtools mpileup^78^ and translated into heat maps representing the distribution of sequences across the population using R and a maximum coverage cutoff was set. Plotting the heat map while setting a coverage maximum demonstrates the presence and absence of the locus in all the isolates.

### Virulence factors

A review of *S. suis* virulence factors was published by Fittipaldi *et al*. in 2012^79^, but additional putative virulence factors have been described since the publication of this article. We performed a systematic search to update the available data and to include these in our comparative analysis. We searched PubMed using the search term ‘*Streptococcus suis* virulence factor’ and included all articles published following the article by Fittipaldi *et al*. On July 16^th^ 2015, this search included 56 articles. Only those novel (putative) virulence factors were included for which sufficient information was available to allow detection of the presence of the gene in our dataset. This information could include a gene identifier of an accessible annotated sequence, a nucleotide or amino acid sequence that could be included in a BLAST analysis, or even primer sequences that could help to identify the DNA sequence of the described virulence factor. Despite our best efforts, not all genes encoding novel putative virulence factors described in recent literature could be identified. In addition, sequences of four virulence factors included in the review from Fittipaldi *et al*., *srtG* pilus, Phospholipase C, RevSC21 and the 44 kDa membrane protein, could also not be found. In total, we included 68 putative virulence factors as reviewed by Fittipaldi *et al*. and we added 16 putative virulence factors identified by our systematic search. A summary of the additional virulence factors is shown in Supplementary Table 2. We list for each of the virulence factors whether a mutant strain in which the gene was knocked-out was created and if it was tested in an animal model. Reference sequences for the identified virulence genes were extracted from 1 of 3 reference genomes: P1/7, BM407 and S735, since not all genes were found in a single isolate. Using these reference virulence factor genes, we identified the corresponding homology groups, which were previously created with OrthoMCL, and subsequently extracted the other members of the group. The virulence factor genes were counted and further processed using R scripts.

### Data Access

Sequencing data for this study have been deposited in the European Nucleotide Archive (ENA) in study accession: http://www.ebi.ac.uk/ena/data/view/PRJEB11219. Assemblies for isolates can be found under accession numbers ERS902322-ERS902419 and reads can be found under accession numbers ERR1055639-ERR1055647. Additional metadata of the sequenced isolates can be found in Supplementary Table 1.

## Additional Information

**How to cite this article**: Willemse, N. *et al*. An emerging zoonotic clone in the Netherlands provides clues to virulence and zoonotic potential of *Streptococcus suis*. *Sci. Rep.* **6**, 28984; doi: 10.1038/srep28984 (2016).

## Supplementary Material

Supplementary Information

Supplementary Table S1

Supplementary Table S2

Supplementary Table S3
