# Supplementary material for: An emerging zoonotic clone in the Netherlands provides clues to virulence and zoonotic potential of Streptococcus suis
Source: Sci Rep. 2016 Jul 6;6:28984. doi: 10.1038/srep28984 (PMC4933891; doi:10.1038/srep28984)
Supplement: Supplementary Information [file srep28984-s1.doc]

## Supplementary Figures and Legends

# An emerging zoonotic clone in the Netherlands provides clues to virulence and zoonotic potential of *Streptococcus suis*

N. Willemse, K.J. Howell, L.A. Weinert, A. Heuvelink, Y. Pannekoek, J.A. Wagenaar, H. E. Smith, A. van der Ende and C. Schultsz


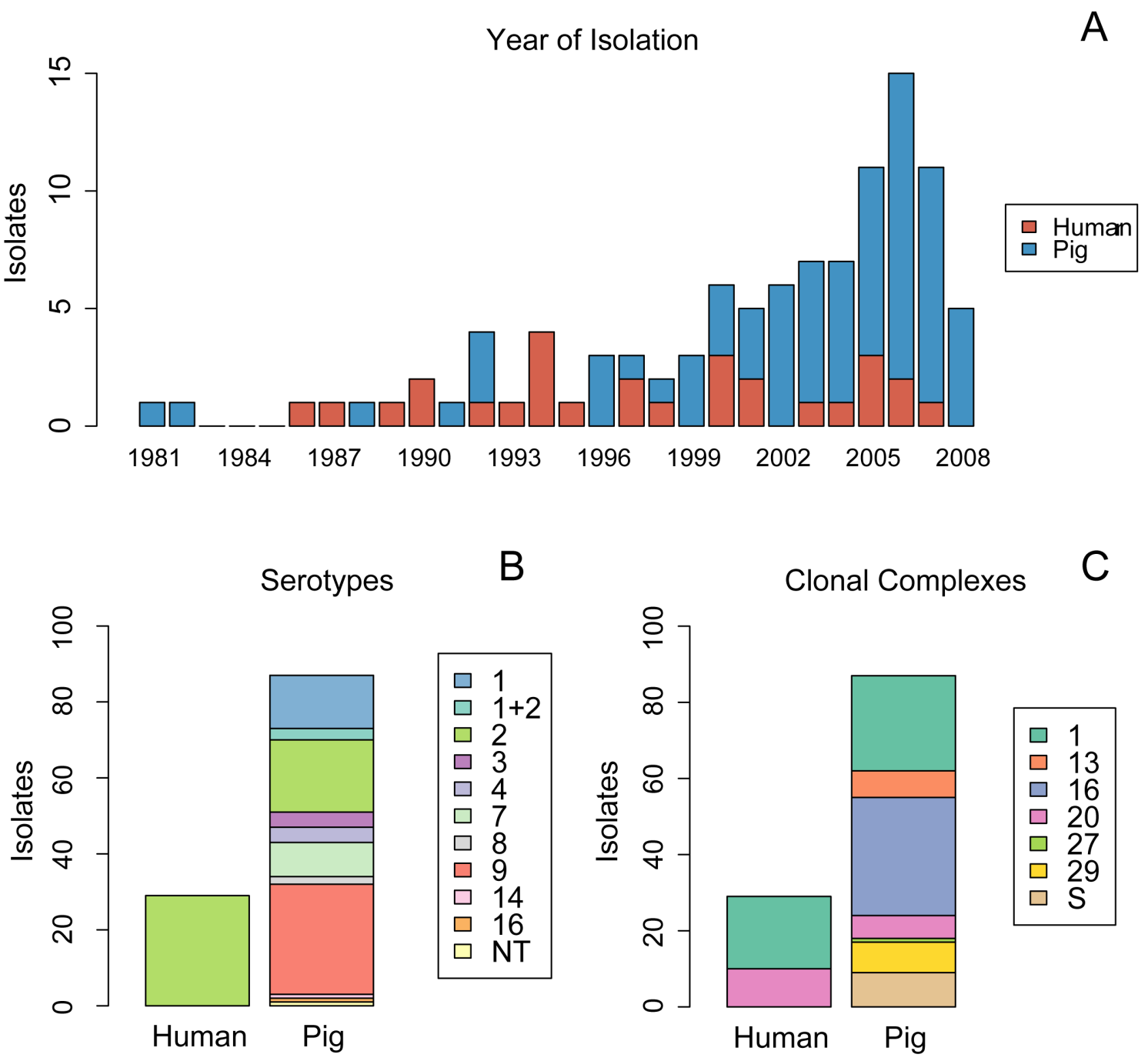


**Supplementary Figure 1** Characteristics of the sequenced isolates of this study. A: Distribution of the year of isolation for all sequenced isolates. B: Distribution of serotypes, separated by host, of all sequenced isolates. C: Distribution of the genotypes as determined by multi locus sequence typing (MLST), separated by host, of all sequenced isolates.


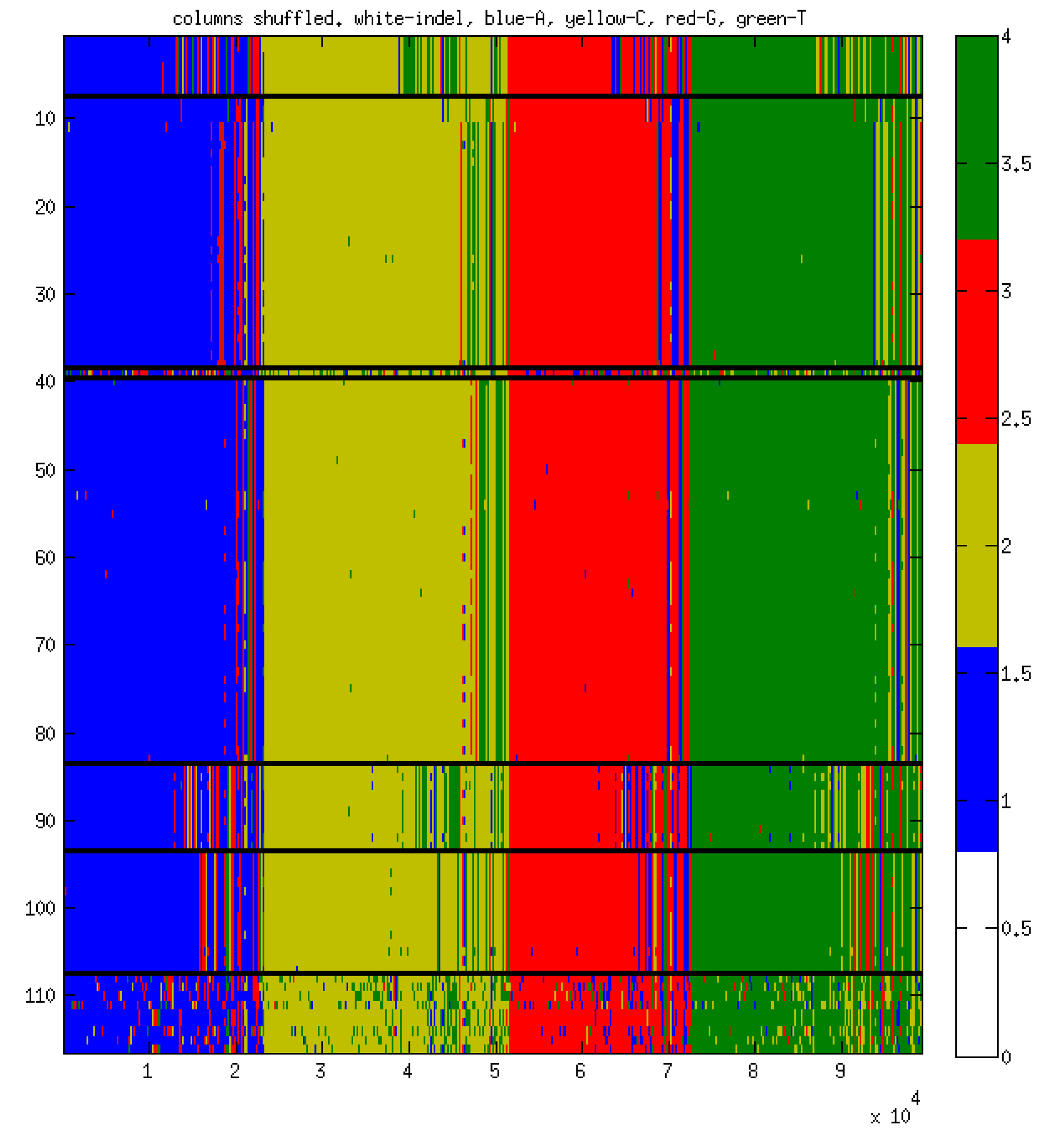


**Supplementary Figure 2** Output alignment for BAPS. A shuffled alignment of the core genome SNPs is divided by black bars to indicate the BAPS groups. Each row represents an isolate and each color represents a different nucleotide. The BAPS groups were numbered from top to bottom.


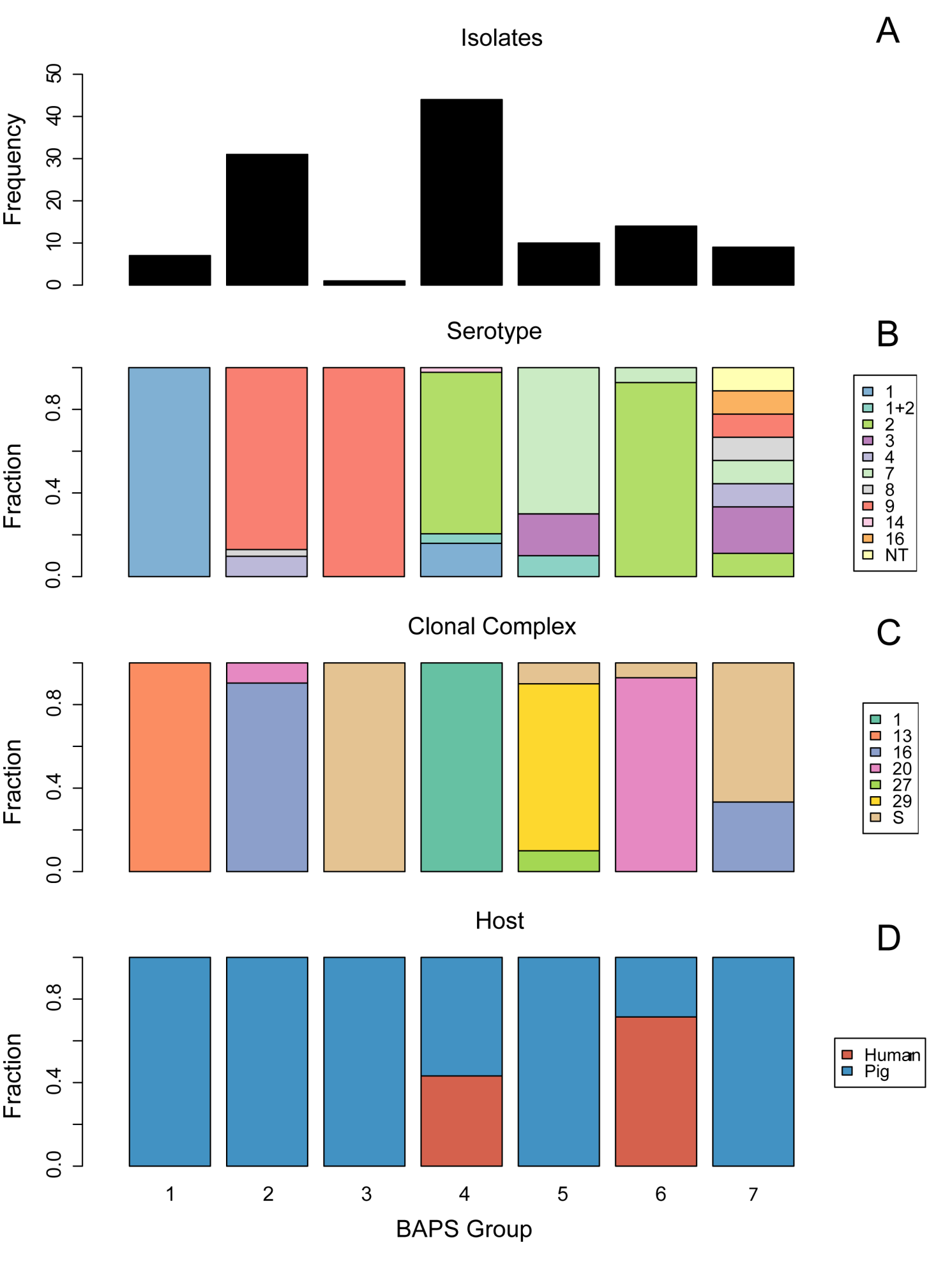


**Supplementary Figure 3** Characteristics of isolates in BAPS population groups. A: Number of isolates that were clustered in each of the population groups. B: Fraction of serotypes present in each population group. C: Fraction of clonal complexes present in each population group. D: Fraction of isolates in population groups isolated from which host. BAPS population groups correlate with genotype, but not with serotype. The zoonotic isolates are clustered in BAPS population groups 4 and 6 and correlate with clonal complex 1 and 20.


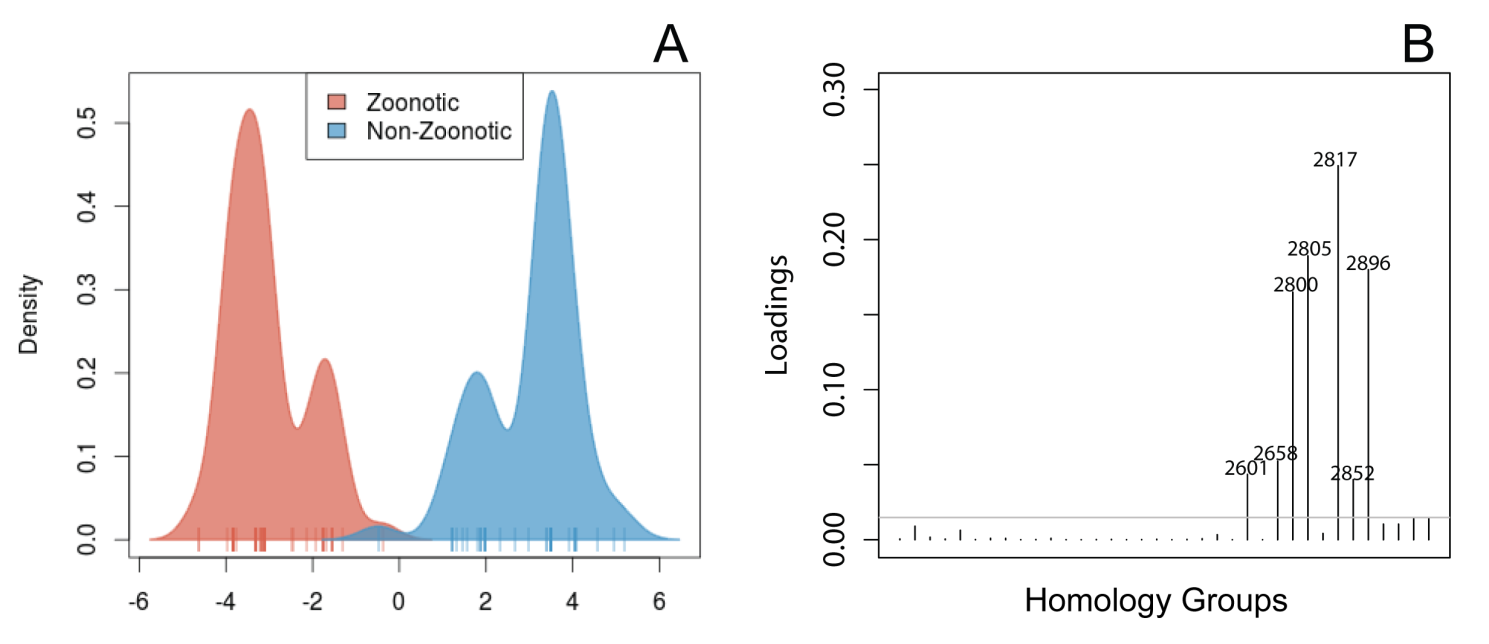


**Supplementary Figure 4** Discriminant analysis on putative *S. suis* virulence factors between zoonotic and non-zoonotic isolates. A: The first linear discriminant illustrates a separation between zoonotic and non-zoonotic isolates with minor overlap. B: The contribution of the virulence genes to the first linear discriminant. The seven virulence genes which provide the highest contribution are indicated with their homology group number as established by OrthoMCL. The corresponding seven virulence factors include a capsular polysaccharide biosynthesis gene, a N-acetylneuraminic acid synthetase, an extracellular protein factor, an Rgg-like transcriptional regulator, an ABC-type multidrug transport system, suilysin and endo-β-N-acetylglucosaminidase D.


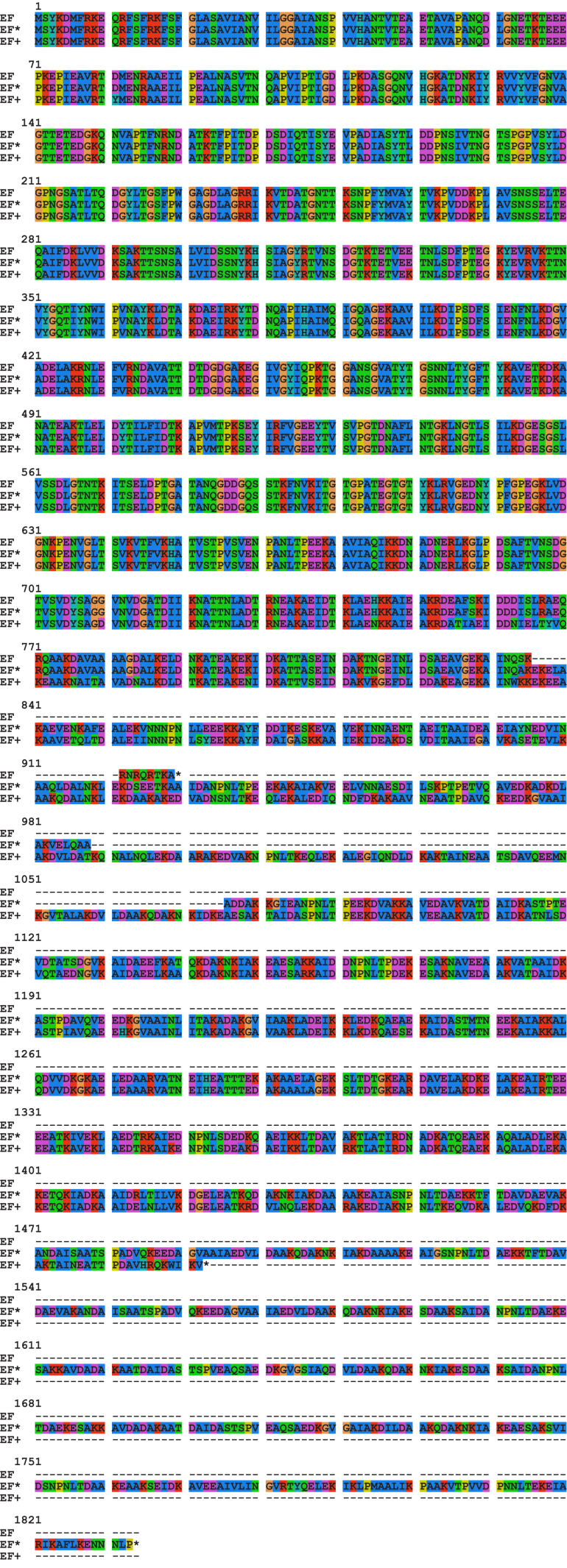


**Supplementary Figure 5** Amino acid alignment of the extracellular protein variants. The alignment illustrates the similarity of the N-terminal region between the different EF variants. Here we aligned representative EF amino acid sequences from the isolates S10 (EF), 901131 (EF*) and 2001171 (EF^+^). The C-terminally truncated EF^+^ protein as found in the serotype 4 CC20 isolates was omitted from the alignment for clarity.


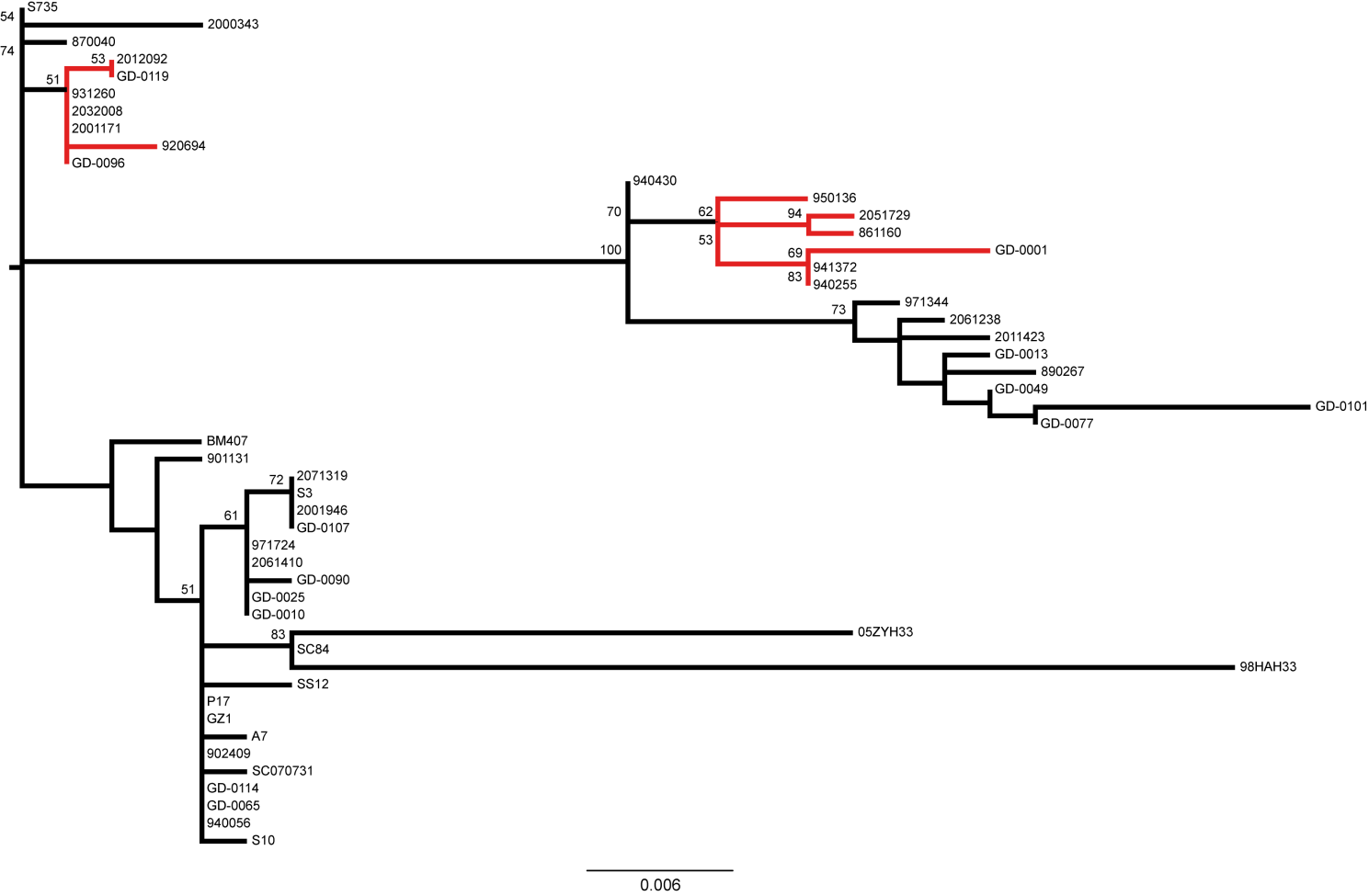


**Supplementary Figure 6** Maximum Likelihood tree from 100 bootstraps based on DNA sequences of the *S. suis* serotype 2 capsule locus. Capsule locus sequences of MLST CC20 isolates and CC1 isolates cluster on similar branches of the tree, whilst having low bootstrap values. The high sequence similarity, as indicated by the fact that isolates of CC20 and CC1 intermingle in the tree, supports the hypothesis that isolates of CC20 obtained the capsule locus from CC1 isolates, as these clonal complexes are highly dissimilar in their core genome sequences (**Fig. 2a**). For clarity only bootstrap values above 50 are shown. CC20 isolates are indicated in red and CC1 isolates in black.


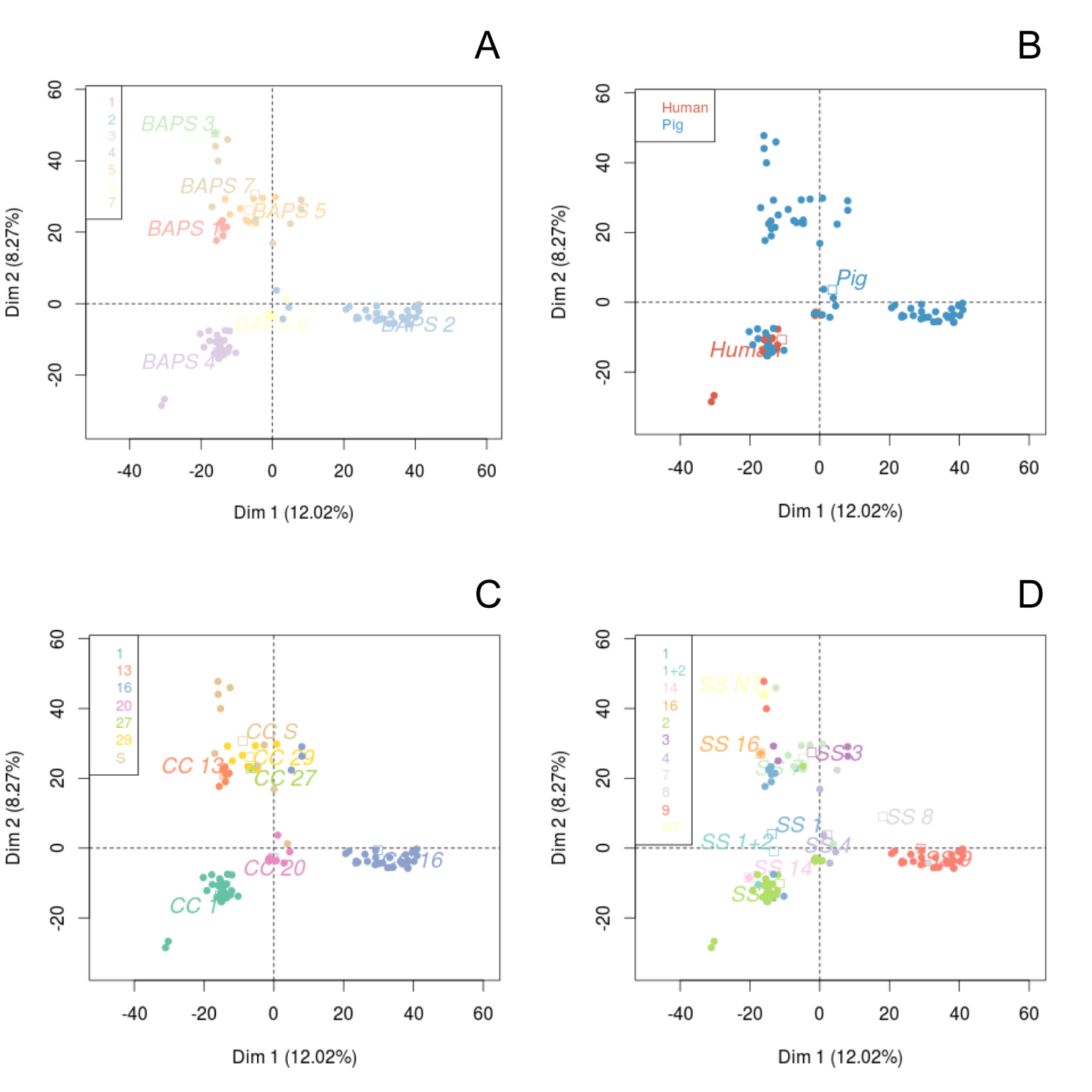


**Supplementary Figure 7** Principal Components Analysis (PCA) on the accessory genome. A PCA was performed on genes of the accessory genomes using a presence/absence table and the first two principal components are plotted. The variance retained in these two variables is shown in the axis labels. The points are overlaid with metadata: BAPS population (A), Host (B), MLST Clonal Complex (C) and Serotype (D).


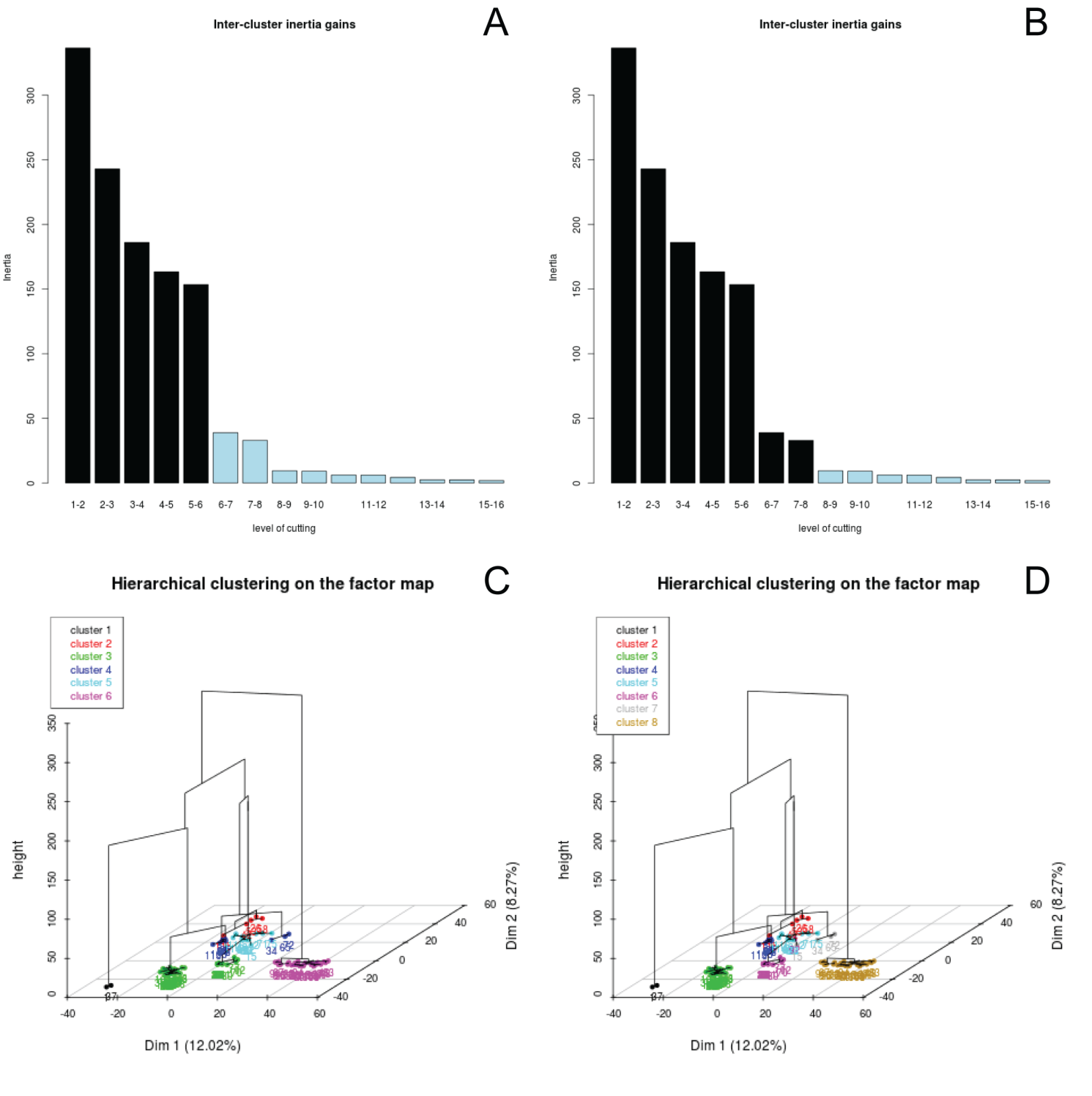


**Supplementary Figure 8** Hierarchical Clustering of Principal Components (HCPC) to identify groups of isolates based on presence/absence of genes in the accessory genome and to establish links between these groups. Plots a and b show the inter-clusters inertia gains which indicate good cut-off points for clustering. Good cut-off points would be at 6 (a) or 8 (b) clusters. Plots c and d show a 3D map of the hierarchical clustering on a PCA plot for 6 and 8 clusters, respectively.

**
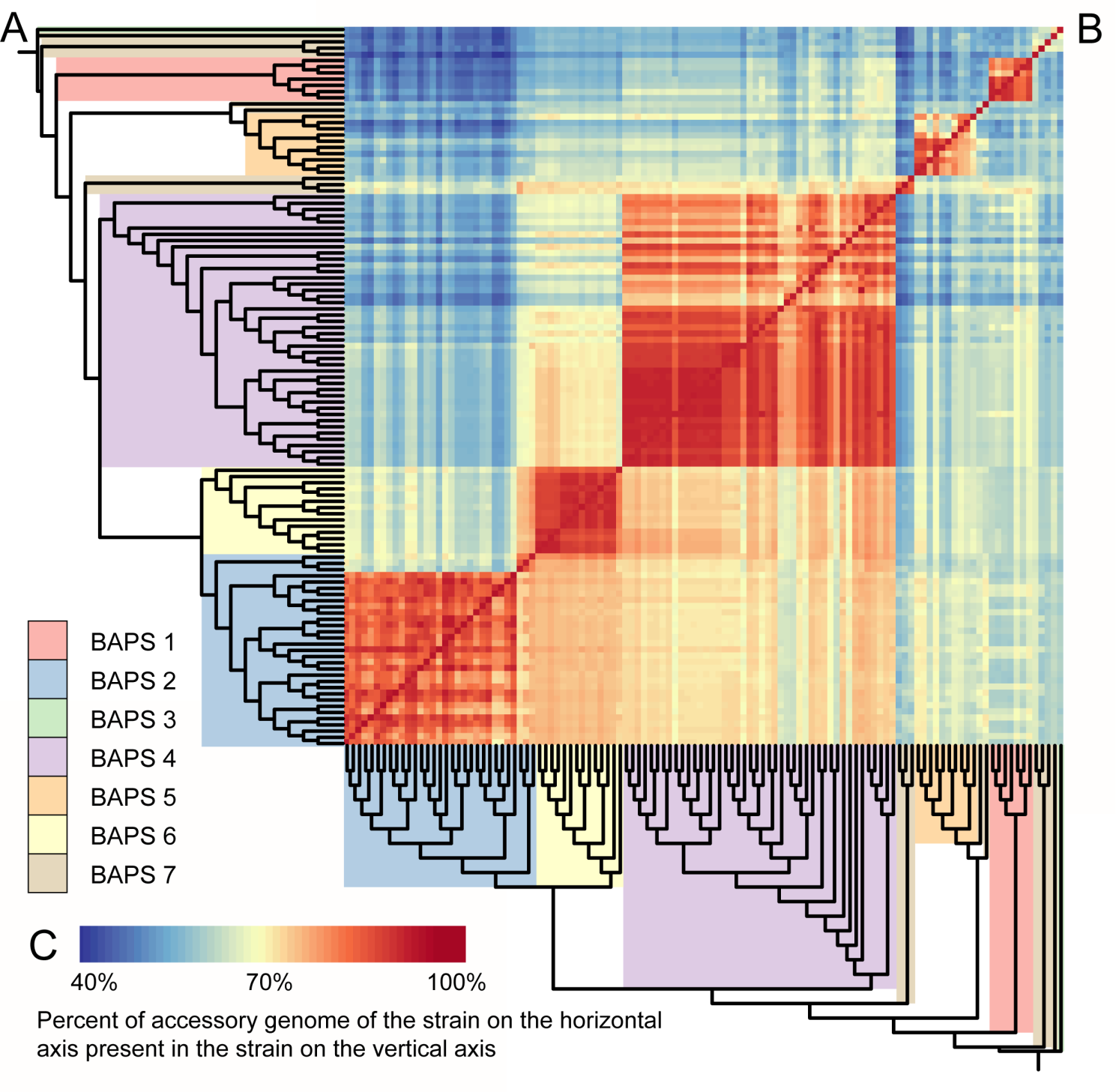
**

**Supplementary Figure 9** Percentage of shared accessory gene content. A heat map was generated by identifying the number of shared accessory genes between every two isolates and subsequently dividing that number by the total number of genes of the isolate from the tree on the left. A: Maximum Likelihood tree generated using Gubbins as shown in **Figure 2a**, except branch lengths were ignored for clarity. B: Hierarchical clustering tree generated on the accessory genome using HCPC as shown in **Supplementary Figure 8c,d**. C: Heat map representing the percentage of genes in the accessory genome shared between isolates. The light red shaded area between the BAPS groups 6 and 4 isolates indicated a higher shared accessory genome content compared to the yellow shaded area between BAPS groups 2 and 4 isolates.


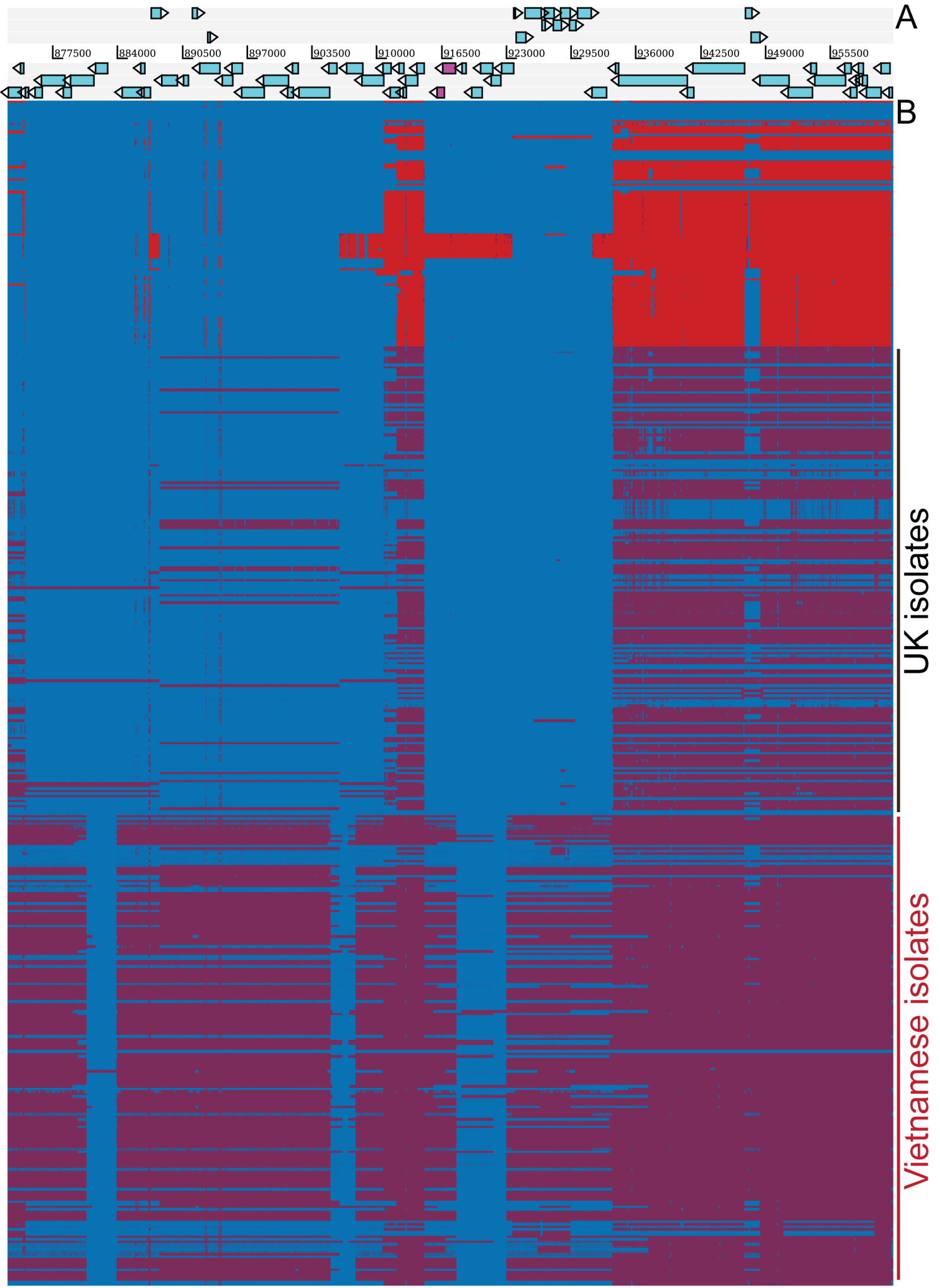


**Supplementary Figure 10** Mapping plot on the 89k pathogenicity island using additional sequencing reads of 375 isolates from the UK and Vietnam (Weinert et al. 2015). The same colour gradient was used as in **Figure 5**, but the maximum coverage was set at 30 as the overall coverage for the isolates from the UK and Vietnam was lower than for the isolates sequenced in this study. The darker red also makes it easier to identify where the additional reads are mapped in the plot. The additional reads were simply sorted by country of isolation and appended to the heat map of **Figure 5**. A: Coding sequences present in the 89kb pathogenicity island of isolate 05ZYH33. The magenta genes indicate the *salK/salR* genes. B. Heat map of mapping coverage demonstrating presence/absence by respectively red and blue. Each row represents one isolate.


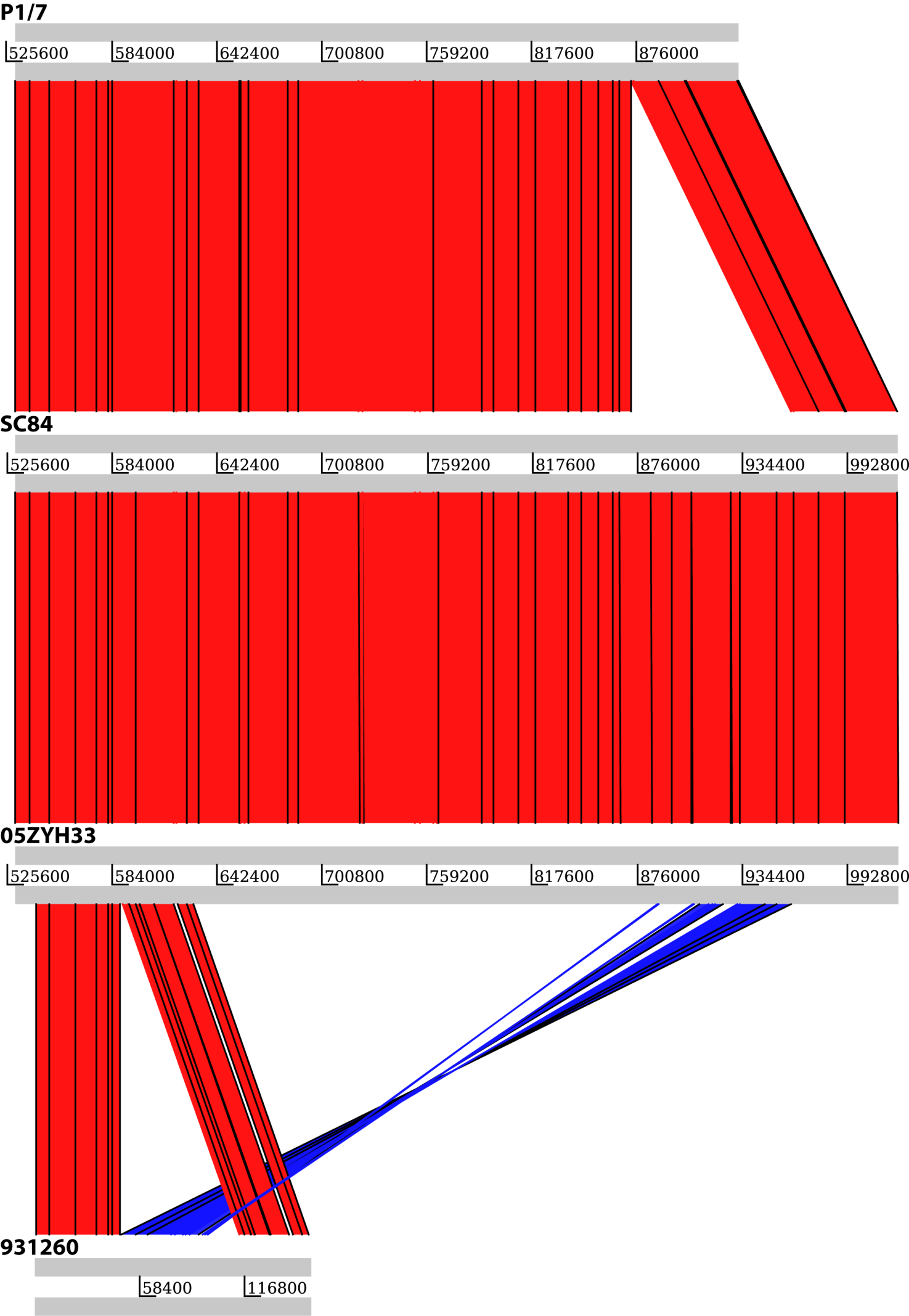


**Supplementary Figure 11** Alignment of isolate P1/7, the Chinese outbreak isolates SC84 and 05ZYH33 and one of the contigs from the draft assembly of isolate 931260. The red blocks indicate regions of high similarity between isolates that are in order and blue indicates regions of high similarity that are in reverse order. The alignment illustrates the 89K pathogenicity island that was inserted into the genomes of SC84 and 05ZYH33 compared to P1/7. It then further demonstrates that a large part of that 89K pathogenicity island is also inserted in the genome of 931260 in a different location. The island in 931260 region of 65988 nucleotides in length was inserted inside a 23S rRNA uracil-5-methyltransferase RumA, which together with the two component signaling system (*salK/salR*) and the low GC content (35.6%) suggests it is a pathogenicity island.


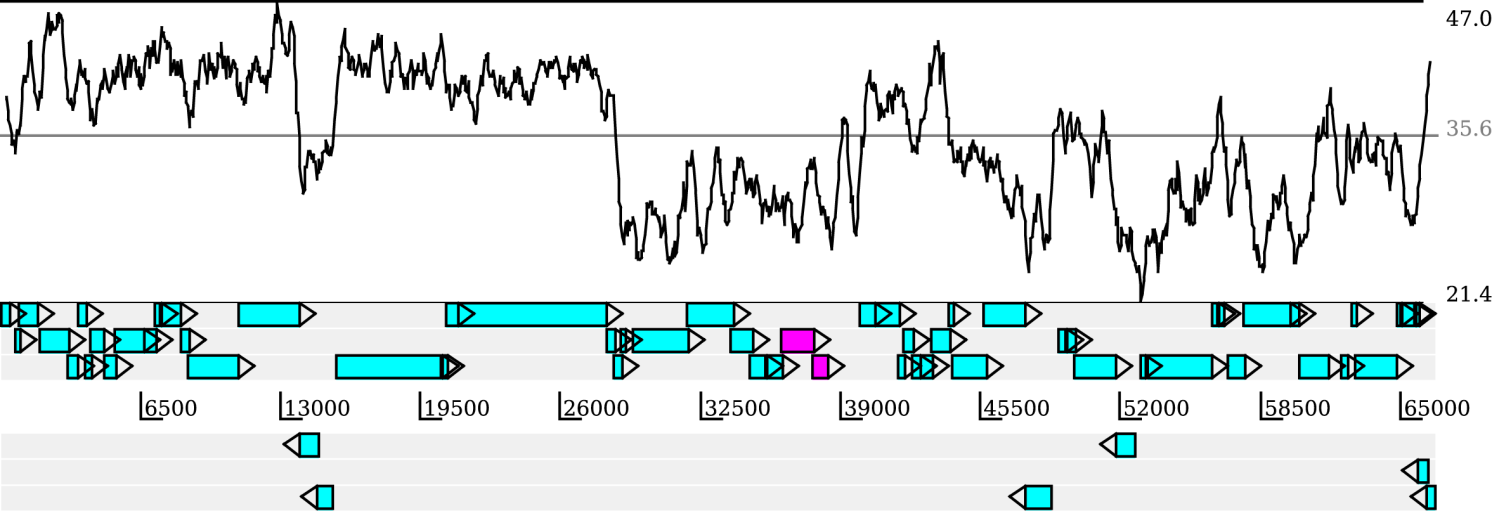


**Supplementary Figure 12** Artemis view of the pathogenicity island in isolate 931260. The average GC content for the entire island is 35.6%, but there is a distinct difference between the first and the second region. The second region contains the *salK* and *salR* genes (indicated in magenta) and has an average GC content around 30%. The first region represents the type IV secretion system which was found to present in many *S. suis* isolates (**Fig. 5**) and its GC content is around 40%; similar to the entire genome of *S. suis*.


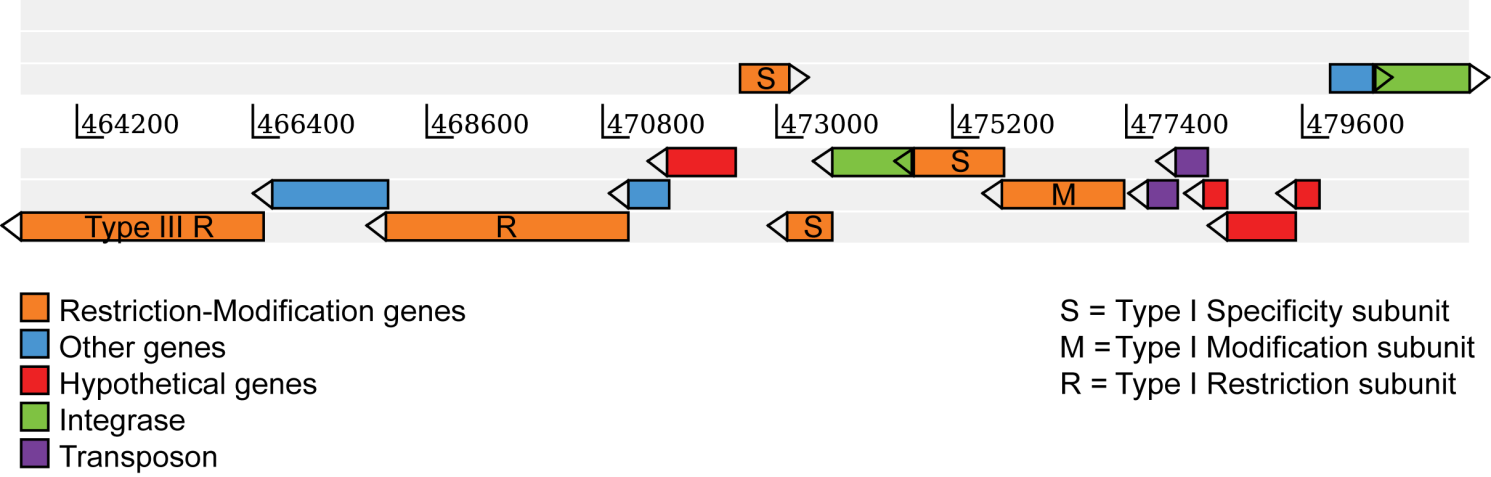


**Supplementary Figure 13** The 18.5 kb prophage region with restriction modification systems as found in isolate 2001171. This genomic region encodes transposons and integrases typical for a prophage as well as several restriction-modification (R-M) genes: A single type III R-M restriction subunit and a complete type I R-M system including a restriction subunit, a modification subunit and three specificity subunits. The most left and most right specificity subunit have switched place in some CC20 isolates. The genes indicated as ‘other’ encode a ATP-dependent DNA-helicase, the molecular Chaperone Tir and a XRE family transcriptional regulator from left to right according to best BLASTp hits.


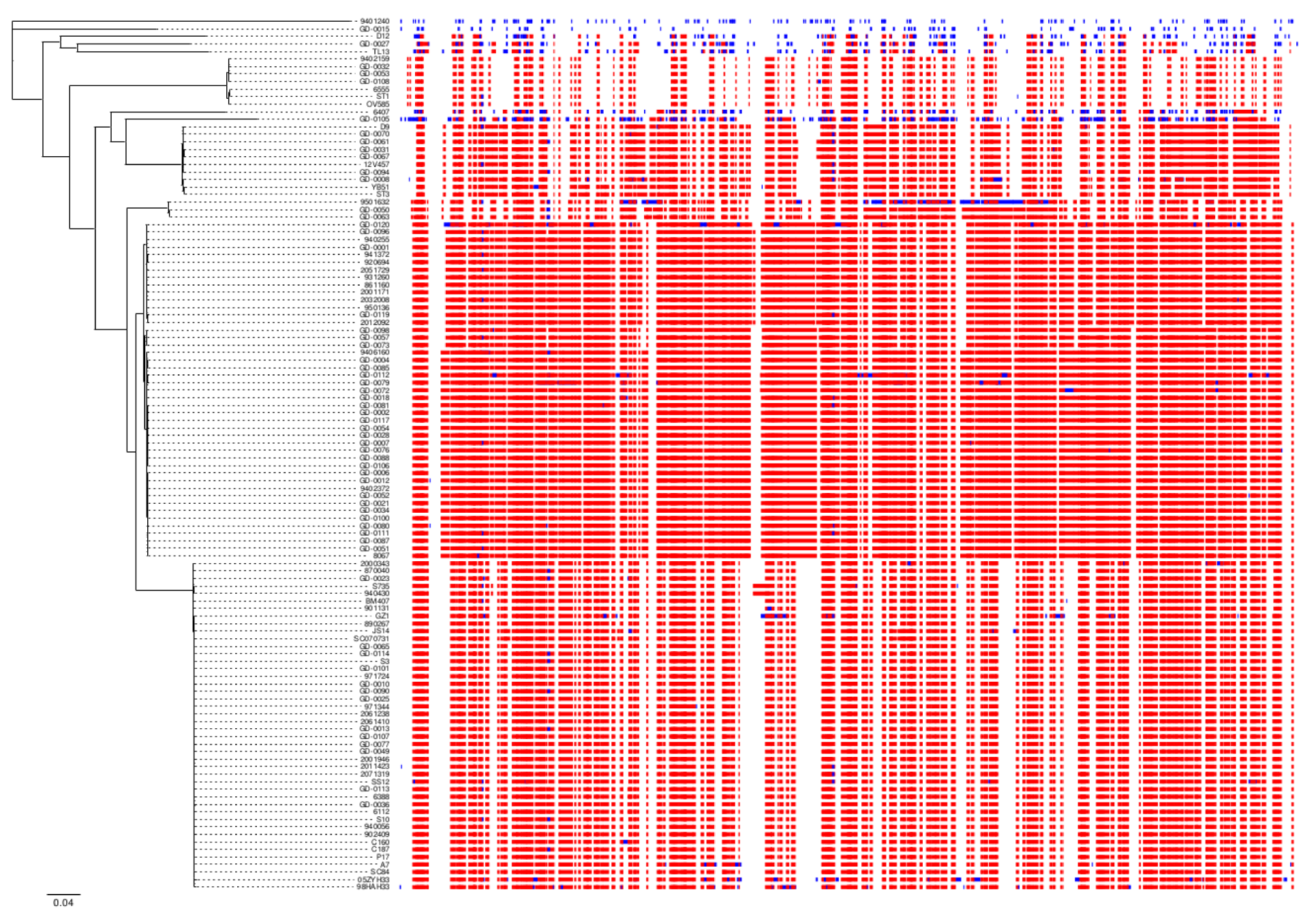


**Supplementary Figure 14** Output of the gubbins_drawer.py script which represent the proposed recombination blocks in the core genome alignment.


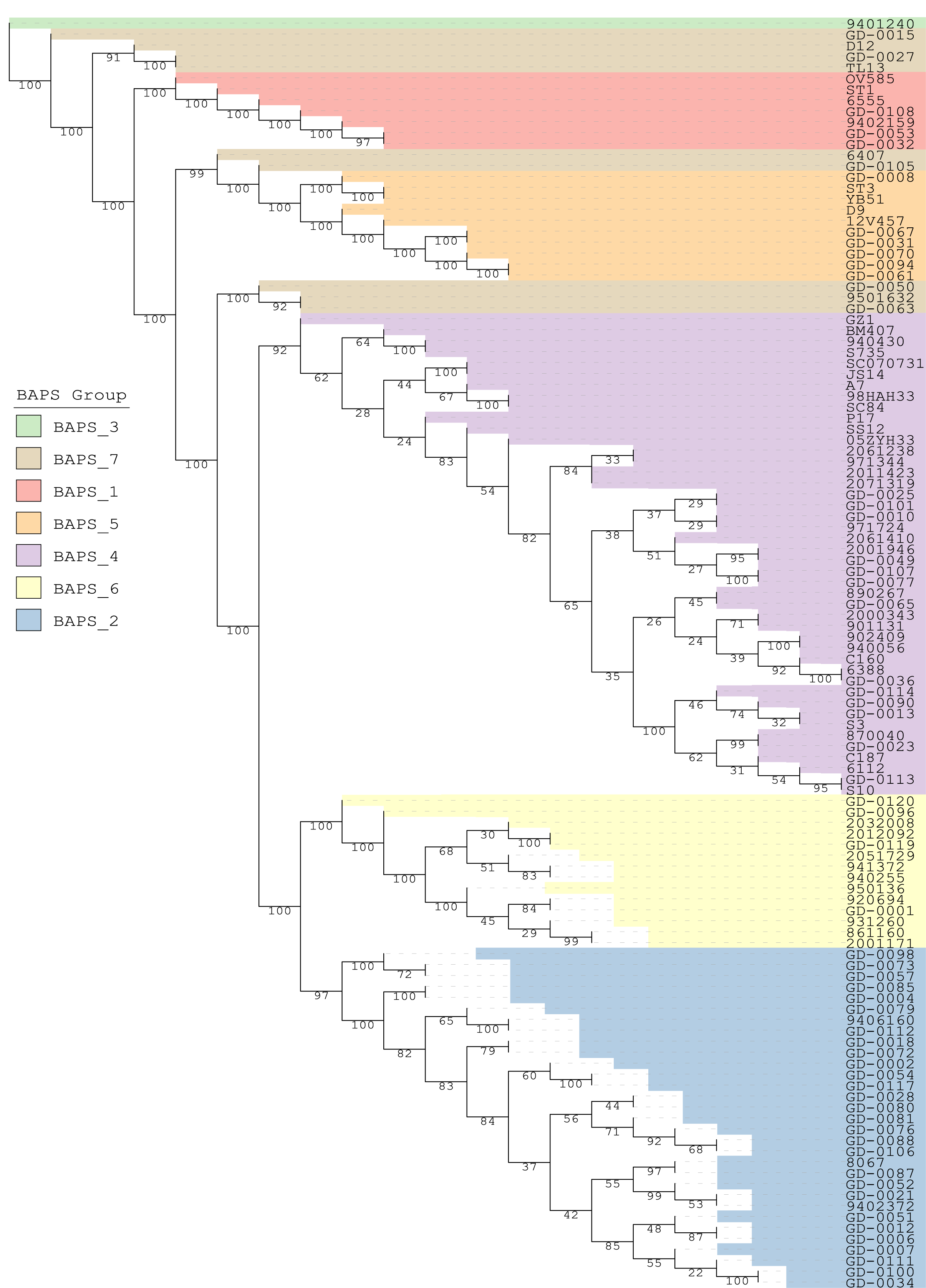


**Supplementary Figure 15** Extended Majority Rule consensus tree created using by using the entire 805775 nucleotide alignment from the 850 genes core genome. RAxML was used to generate bootstrap replicate files from the alignment and to create parsimony starting trees for each bootstrap replicate. ExaML was run for each bootstrap replicate with parsimony tree. The built-in bootstopping creating in RAxML was used to determine when convergens was reach which was after 400 bootstraps in our case. The Extended Majority Rule consensus tree was determined with RAxML. Bootstraps are indicated on the nodes, but branch length are not available for a Majority Rule tree. BAPS grouping is plotted over the tree to illustrate the similarities between this tree and the tree generated by Gubbins in **Figure 2a**. High bootstrapping scores confirm the branching identical branching and support the ML tree generated by Gubbins.


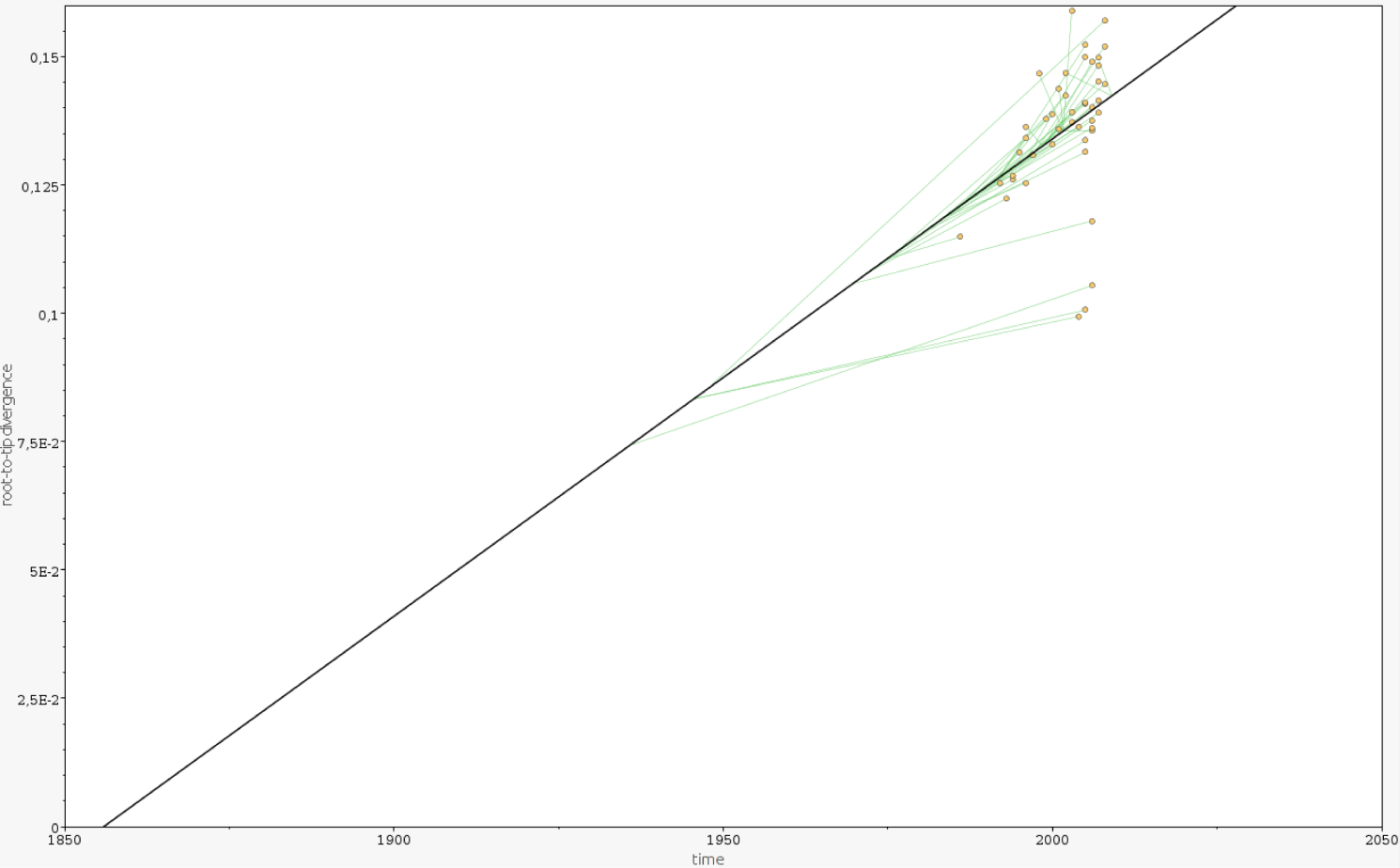


**Supplementary Figure 16** Results from the Path-O-Gen analysis of the CC16 and CC20 isolates using 1340 non-recombinant SNPs in the core genome as determined by Gubbins. The R squared value is 0.13333.

## Supplementary Tables Legends

**Supplementary Table 1** Characteristics of the isolates used in this study. Table listing the metadata for all 116 isolates included in this study.

**Supplementary Table 2** Table of 84 virulence factors included in this study. The list includes 16 new putative virulence factors identified after publication of the virulence factors review by Fittipaldi et al. The remaining 68 virulence factors have been described previously by Fittipaldi et al.

**Supplementary Table 3** Assembly statistics of the sequenced isolates. Table describing the assemblies of all 98 sequenced isolates after removing the low coverage (<10) and short (<300 bp) contigs. N50 is the length of the scaffold that spans the 50 percent mark of the total length draft genome when ordering the scaffolds from longest to shortest scaffold.

## Supplementary Note 1

### Sequence of the pathogenicity island in isolate 931260

Below is the sequence of the pathogenicity island as found in isolate 931260 starting from the first nucleotide difference with P1/7 and until the last nucleotide difference with isolate P1/7. The length of the sequence is 65988 nucleotides.

>sequence

cgttgagtgcgtagtgttgctacaacgaagcaacgggtaaaaatcctttattttaagcactttttcaagcattttgtctttattgaaaagagtgattttgacataaaaaaggttcaaaaaaagtacattgatgtgaacgtctgtttggatgtcgactgcgtagacaaaaaatagatacgtcataaaatataatagttcagcgagaacattttaataagtgttctcgcttttttaatttcaggaggaattcatgcaaaagaaggaacaatcatcacgccaaatcgtgatatgtcatctcgtgactattttaggagttgatattgtgaaagcaactcagctaattgatgaaatggaacaactaggtttaatccgatttgatgaatttggaaatgttggaatattagtcttggagggacaatcatgaaacgaattaccgctaatcagtatcagacatcagagcgttattataaactcccaaaagtcttgtttgagagtgaacggtataaggatatgaagctggaagttaaggtagcctacgcggttttaaaagatcggttggagttgtctttgagtaaaggttggattgatgaggatggggctatttatttgatttattccaattcaaatctgatggcactattaggctgttcaaagtcaaaactactctctatcaagaaaatattacgcgaatatggcttaattgatgaagtccaacagtcctctagtgaaagaggtcgaatggcaaataaaatttacttgggggaattagaacatgaaactaccccagtcttacatacagacggggctagtgttaaaaaaacactaggggggtctcaaagaaagacggggccggtcttatattcagcccctagtgagactgaaggaagtgagactgaatatagtgaaactgaagggagtgatttccttattgaggacgaggaggagaggcagctagtagatgagaaacaagaagaaaactttacttcaaaagtcgatggcgtgaccaagtacgatcgagactatatttggggtttggttcatgaccagttaagacagacaggtctatctcagtcggctagtgactatgccatgctctattttagtgaccgttatcagtatgcattggaacatatgcgatttgctcggtcagcggaagtaatagctgaatacgtatttaatggtgtgctgtcagagtggaccaagcaactgagacgacaagaagtaaaaggaggtgaataaggtgatttggtggatactaggtggaatctatctgatttctatcatcattttgattgttgaaatcatccgtgcaccagaaatggatgatcatatataagcaagagttttacaagtgtaaggctcttttttagttggaaagagaggaaaaatgaaatttttagatttatttgctggaataggcggttttaggctaggaatggaatcacagggtcataaatgcctgggcttttgtgaaattgataaattcgctagaacatcttataaagccatgtttaacacagaaggggaaatagaataccatgacattaaagaggtcacagaccatgactttagacaatttagagggcaagtggacatcatctgcgggggatttccttgccaagcattttcactcgcaggcagacgattgggatttgaagatactcgagggactctcttttttgagattgctcgagcggccaaacaaatccaaccacgttttctatttttggaaaacgtcaaagggctactcaatcacgacgagggacggacgttcgccacaatcctctccacgatggatgaattggggtatgatgtcgaatggcaggtgcttaacagtaaggacttccaagtcccgcaaaacagagagcgggtctttattatcggacattctagaagataccgttccagattcatatttcctctcagaagagaaaacagcccagctcatcttgaaaggctaggaaatatcaatccctctaaacgtggttttaatggtgaggtctatctgacgagtggacttgctcctacactaacaagaggtaaaggagagggagctaaaatcgccattccagtcttaacaccagatagactagaaaaacggcaacatggtcgtcgatttaaggacaatcaagaccctatgtttactttgacaagtcaagacagacacggagttgttgtcgcaggaaatctgccgactagctttgaccagactggaagagtatttgacatatctggcttgtcaccgactttgaccaccatgcaaggtggagacaaggtgccaaagattttactgagggaggagctgccatttctgaaaatcaaggaagccacaaaaacagggtacgcaaaggcaactcttggagactctgtcaatctggcttatccagactcaaccaaacgtaggggacgagtgggaaagggaatatccaatactctaacgacttcagacaatatgggagtggtggttgctgctatggaatatcgacaggctaagtggtatgaagtcacaggcattgtcttagaggggaaactttatcgcctgagaataagacgactgacaccaagagagtgtttcagacttcaaggctttcctgattgggcttatgaaagagcagagagtgtttctagtaagagccaactatacaaacaggccggcaatagcgtgactgtcacagttattgaagccattgccagagaatttagaagaacggaagaggaagaaaaacatgaacctactacataagaaaagtatcctagattgtactgaattagaagaacatattcaccaagttgaaactaatcagttattacaaaagatactgtcactccccaattttgattgtgactttgaggtgacttttgaagatgattaccacaaagagatgaatgatcccctattctacgaatccaatcttcatcggatttcggattttatggaaactagggatattaaaaatggtgtagatacactattgacgaaagacaatcacctggcctttcgtgcctttggtgaaaattattctgctaggggaaaggatggcattttaacgactctagtgacggtcaagtgttttggtgaaggacggatgcccattgatatgagtcgctatttctcaactccagaaccaacagttgaaaatagcctaaccctataaggaggagcctatgcttgaggtatatctaggaaataatgccaatacaaatcaagacttactagccattttgacgacctatggtgtgacttatcgttgtataaaagcgtgtgatgtaaaccgtgaaatcttactgtcactctttgctaaaaccactgattgttttgagttgctgtcgccacgatttcttcgctttaagcgtcaatatacgataagtttgaatgagatgattcagctcattctccaaaagccagaccaaaaccttcgtctgcctctcattgtctgtcagaatcacgtctatccagctattgggctagacgaagtgcgaacttttcttcccagacaggtaaaagaagagctgtttcagaccagtctgatgaaacaagtgacaggggatgtgtgaagatgaatgaacgattttgggacaatttagaaatcattctggcagaaaaagacctcacttgggcagaactagctcgcaaagtattcaacggtcaatatgtttatccaagtgagtttaatcgcctctatcaaaaattacggcattacaaatcgaatcgtctgatgccgcaaactagatgggttgagcgaatcgttctagtcttagatattgattatgaagatttattcaagaggtgacaatgataaggatagtgttgttttatctagccatacagcttaatggacttctggtgagtttatacctgaaagagtatctgacaatagagggtatagtcttgctacaattggtcctattaagtgtgacttgcttagagattgcccgtcataaaactgttcaagcaaaaaatataatcttaagaaatcgcctaagttggttgcttcttggttttgtgtgtatggttgcttttgcagtcttcatcagtttcctattttcagttcagactaggaatcaagcggtcttgttacaagtaggaaaacaggttcctcctattatctttttattgtttctggtcaatgcaagtctccttgaagagattgtttataggcaattgctgtgggaaaaattgacattcccttttgtacaaataggcgtgaccagttttctctttgttctatcccatggtcccaatcagttagggagttggctcatgtatagctgtcttggcttgaccttggctgttgttcgattgaaaactgattgtatgacggcaatcgccttacatttactttggaatagtttggtttatgtcttgaccttcttgtgataccaaaatcaagagtgcttccgtattatggaagcaccttatgtttgatagggaatcgaaaaaagaggaggtcaccatgtcatctgaacaacaagaacgtcaagcgatgcaatacgtggaaagaagtagtttattgacagttagaaccctcttaaagctcttagagtggtcggctaggcaagccttagctcaagattcagcttataagattggggttcaaaaactggaagaactccttcaaagcccttacgtcattgaagcgattgatgttagtaaagtcattctagataagccaatcgatgtggagagatttaaggagttgatggagtcagaaaaactgccaatcgcaattagttggcaaaaagactatctctacttttatgccaaggataagaccttgcttgatcatcacttggacgaacttgtgaagaaactgatgtctaatccagaaaagttagaaggtctaacctttgataaaaccttggaccaagagatagaactggccaaagaaaaaataagagtgacagaaccttcggcagtcaaaaccaaggaggtgaccttgtaatgtattcaagacgaaaagctttagtctttggactcatggggctcgcctttggttatttctgccatcgtctaaccctgctctatgatagtttaaccaatgccccacctatggaacgttttgcctacctcttaggagaggggctaaatcaagtcttcaatcctttatggctattttcctttactcaaaaatctcttcttgcctttatccttggggttctaacgatgacactagtctatctttatgtatcgacaggacagaaggtctatcgagaaggggaagaatacggttctgcacgatttggaaccagtaaggaaaagcggaacttttacagtaagaatcctttaaatgacacgattttggctcgtgatgttcgtctaaccttgttggaaaagaagaaaccccagtttgaccgaaataaaaatttgattgtcattgggggttctggggcaggtaagacctttcgctttgtgaaacccaaccttatccaacttaattgttccaatattgtcgtagatccgaaagaccatttggctgagaagacaggtaaactctttttagagaacggttatcaggtcaaggttttagacttggttaatatgaccaattcggacggttttaatccctttcgttatgtagaaacagagaatgatttgaaccgcatgttaacggtctattttaacaatacccgtgggtctggttctcgcagtgatccattttgggacgaggcttccatgacattggtgagagctattgcctcttatttggtagatttttacaatccaccaggaagttccaagcaagagcaggaagcaagacgtaagcgtggccgttatccagccttttctgagattgggaaactcatcaaactcttatcgaagggagacaatcaggacaaaagtgtacttgaagtcttgtttgaagactatgctaagaaatatggtcatgagaactttaccatgagaaactgggcggattttcagaactacaaggacaagaccttggattcggtgattgcggtcacaacagctaaatttgccctctttaatatccaatcggtgattgatttgacgcaaagagacactatggatttgaaaacgtggagcactcaaaagaccatggtctatcttgttattccagataatgacactacctttcgttttctatctgcattatttttctctacggttttctccactttgaccagacaggctgatgttgactttaaagggcaactgcctatccatgttagaagctatctggacgagtttgcgaatgtctgagaaatcccagatttcgccgaacaaacctcaacagttcgatctagaaacatgagtctagttccaatcttacaaaatatcgctcaactccaaggactttataaggaaaaagaagcttggaaaactatactgggaaactgtgatagcctcctctatttgggtggaaatgacgaggaaactttcaaatttatgagtggtcttttaggcaaacaaaccgttgatgtcagaagcaccagtcgttcttttgggcagactggttcaagttcgacctctcaccagaaaattgcccgtgacttgatgacggctgatgaagtcggaactatgaaacgagatgagtgcctcgtacgcattgcaggggttcctgtttttcgaaccaagaaatattttccactcaaacataagaattggaaatggcttgcggataaggaaaccgatgtacgctggtggcactatcatatcaatccactaaccgctgaggaagaggtagatttgtcaggccataaaataagggatttaagcacagaaacgacactacattaatagaaatgaggaattatatgaatcaaaaactaacaggctttgtttacggagtggacgccagctccatgttctcccaagccatgtctctattacaaaaaggcttaattgcagtaggagcctttcttgttgtcatgggcattatcaacctttccaccaacattaaagatggtggggcaggtgtccgaaatgccattctagaaattgttggtggggtcatggtgggagccgctggtgcttttgtgacacagattaccatttaggggaggacagcacatgacatgataatgaattttacgtcaccttttgtatttctagcctctgaaaaagtatctagcgatagcctttttgaagggtttcaagtggatttagaatcaaccgccaacttggttaagtcactagcggactttaatccgacggtgtggtcttacatgacagccattaccaaggggattatgcagcccttgggagtagctattcttgcggttgttctcgtactcgaattttcaaaaatggccaagaaaatcgcaaactcaggtggtgcaatgacctttgaagccatagcacctatgattgtcagctacatcatggtcgcgatcgtgattaccaatacgacggttattgtggaagccattattgccactgcctcttatgttattgaacaagtggcaagtcttgtcacgaatggcggtgccagttacgataccgtctcaggcatcaagggttctggaattgtaggaaaaatgattattggcttttttgcgattctcatttggttggtacgaatggccagtatcatggttgtcaatatcttgattaccattcgttttatccaactctatctgatgataccttttgcccctgttaccattccaacctttcttagtgatgactggagaagtgttgggattggctatcttaagaacatcatggtctatgccgttcaaggtattttgatttttctaattgtatctcttgttcccttgtttgaatcggctggaaagattgccgtatcaaatggagctggagtgatggaatcactcgccattatgtttggtggcttggtacaggctatattgttaatcattgccttagttggcagtcaacgaaccgcccgaagtattttggggatgtaggaggaagggagtcattatctcgcctcctctttttatagcttacttagattggagaacatttatgaacactcgtgtctttaaggacatctcaaaggttcaacacagggcatggctgggatttacaactagacaggttatttttgtatttccagctgtcgccctaaccattttggtattgggcttgaacctattttactggcaatttggggattggtttgtctatggttttgtttttagctttaccattccactcatgttatttggggtctatcgacccaatgacttaccatttgaaacctatctcaagtaccgttttcattatgaactaacggtgccagaccgcacatttactggaagaaaaggagaccaacgtgaaaaaattaaaacactcaatgaaaccaaagacctcttctaatgacaaaaatcaaaagacgaaaatacagaagcaggagatcagaccttctaccgtaaatacgttagcctatcaagggctttttcagaatggccttatgcaggtcagtccaagctatttctcacaaacctatcttttaggagatgttaattatcaaacaattggcttagatgataagggagctatcgtggagaaatattccgatttaatcaattcactggatgatcagaccaatttccaactgactattttcaatcaaaaagttaatttggaaaaattccgtaagagtattctctatcccttgcaggaagatgggtttgatgcttatcgtgatgaattgaatcgcatgatggatgccaacttagaggctggtgagaacaacttttcagccgttaagtttctttcatttggcaagagcgaccaaacaccaaaactagcttttcgttcactgtcacaaattggggaatatttcaggagtggcttttcagagattgatgtggctcttggtctactcggtggagaggagcgagtgaatgtccttgcggatatgttgcggggtgaaaatcattcaccgttttcttacaaggatttgaccctgtcaggtcaatccactaaacactttattgccccaacctacctttcctttaaacacaagaatcatattgaattggacgatcgattattacagattgtttatgttcgtgactatggtatggagctaggggataaatttattcgagacttaatgcagtcagatttggaagtgatgattagcctacatgctaaaggctctaccaagtctgaaaccatgaccaagctgcgaaccaagaagaccttgatggaatcgcaaaagattggggaacaacaaaagatggctcggactggaatctatttggagaaggtcggccatgttcttgagaacaacatcaatgaagcagaagctcttcttcaaaccatgacccagacaggagataaactgtttgacactgtatttctaattggcgtgctggcggatactgaagaccaactcaaacaatctcttgatattatcaagcaagttgcagggtctaatgatatgattatcgataacttgacctatatgcaagaagcagcctttaatagtctcttgccatttgggaagaactatctcgagggtgtttctcggtctctattgacttcaaacattgccgtgaatgcaccttggacttccgttgatattcaggacaagggtgggaaattttatggcatcaatcaaatctcaagtaatatcatcagtattgaccgtggtaagttaaatactccgtcaggtttgattttagggacttctggagctggcaaagggatggcgacaaaacatgaaatcatctctactaagctcaaggaagcagattgcgatactgaaattattattgttgacccagaaaacgagtacagcattatcggtcaagcctttggtggggagagtattgatattgccccagactctaccaccttcttaaatgtcttagatctatctgatgagaatatggatgaggacccagttaaggtcaaatccgaatttctcttgtcttggattgggaagctcttggatcgcaaaatggatggtcgtgagaagtccttgattgaccgtgtgacacgactcacctataagcattttgagacaccgtctttggtcgagtgggtctttgtcttatcccaacaacctgaacaggaagctaaggacttggcactagatatggaactctatgtggaagggtcgctggacatattttcacatcggaccaatattaaaacagacagtcatttcctgatttacaatgtcaaaaagttaggcgatgaattaaaacaaattgccctcatggttatctttgaccagatttggaatcgggtggtcaaaaaccaaaagctagggaagaagacctggatttactttgatgaaatgcagcttctcttattggacaagtatgcctctgattttttctttaaactttggagtcgtgtccgtaagtatggggcgataccgacaggcattactcaaaatgtggagacacttcttttggatgccaacggcagacgcattattgccaatagcgagttcatgattcttttaaaacaggctaagagcgatcgagaagaattggtgcatatgcttgggctttctaaagagctggagaagtacttagtcaatcctgaaaaaggggctgggctaatcaaggccggttcaaccgttgttcctttcaaaaataagattccacaacacaccaagctctttgacatcatgagtacggaccctgaaaaaatgaggacatgagatgaaagaggataaaaagctagtcaaacaggctaggcaaaactttcgaagcaacttaaaatcagcccgtatgcattatcggaaagaagttaggaccttaagacagactgttcctaaaaaaggcctatttcgaaaaccagcccaaaattctctttttcaagataagaagatagagttaaaagaaaatctactcagtagccaaaaggaagcagaagagaagttcctaaaagaaattacctatgtgtctcctagactgttgaaggtaaaggaaatcaagaactatcgacttcctcaagctcaagagcgtttgcggacggcaagaaaacatttgtcagaagtgaaactaagtgagaagcaaaaggcagttaatcccaagtttactttccaaaaagaaaatccttccctgaagtctcgctttcagtttcaccaagaaaaatcatttgatcggctaggtgcagaaaaagacgtaagttctgccaagcgtgaggttaaacaactcaagaaagtccaaaagtctaagaaaaactctaccaaagtcaaagttggattaggcttagctgcatctgaatcgcttgacttggtagcacaggatgatgatttagatggtctaagaaccttaaaggatactagcttaaaagccagacgctatggcaggtttacctttcaagcaggtaaggtggcagtaaaaagtggacagacaggtgtgcggtttaccaaaacaaaattttctcatggaaaggaacgattccagaacttcaaaaagggaaaaggattcacacgccagaaacctcttaaaccaagaagacgctaccaaacctttttaaagcttgccagaaaacaaagtgtcgcaggtttcaaaggaatcgtccaagccattaagggaagtctgaccttcttttctgtccttgcgggaaatcctttgacttggattgtctcaggcgttctcttgatactccttttgatgatgagctttttcatgagtgtctcaggtagcagtgtcattcaacaagatgagatagaattgagtaagagttacacccacatgacgtgggaagatgcggagcatacaagaaccaacgaaaagggaattaccttttacaccaaggttgatgagattatggtttatatgaatcatcaataccaagactataagcttgacgatttcatggaaacgggtggtactacctacaaagcatttctcagtcaggtgtggacggacttaaatggtggagattctattaaatccatgtctgacttatataaagaacctgcttacaagctgtctgatgaggatcaagaagaactaaaggaattaatcgaagaaggcaactatctagcccttcaagaattggacaatccctttcagggacagaccgatgaggatagcttaaacatgacctaccgatatggttatgaggtcattgatgagaaaccaacgcttcatcaccatatcatcttagaagcaaaagaaggccaagtcattgtggctccgatggatggtaaggtatctcttgatggagaaaacattgttttgacatctggtaagggagtgaataagactaaactaaccttgtttggcattcattcaggccgagtgagcgaaaatcaacaagtcttggcaggagacattattggtcagaccaaggatggaacgggtctaaaaatcacctatcaaaaggttgatggagacacggataagctagtctatgtcaatccagctttctactttccaaaagtaatccaggttcagaccaccattcttccaactatcggtcagtttggcggcgatgagttcgagagagccaaggcaatttatgactacctcaaaagcaaaggtgcgaccaatcaagctatcgcagccattctaggaaattggtcggtagaatcctctattaatcccaagcgtgcggagggcgactatctatctccccctattggtgcgacagacggttcgtgggatgatgagggctggctttcactcaatggtccaactatatataatgggcgttacccaaatattctcaaacgtggtttaggcttaggacaatggacagataccgcggatgggtcacgcagacataccttattgctcgaatatgccaaaggaaaacatcaaaagtggtatgacttaggcttacaactggatttcatgttgcatggggataatccttattacatcaactggttaaaggactttttcaaaaattcaggaagtccagctagtcttgcccaactctttctcatttactgggaaggaaatagtggtgataaactacttgaacgccagacaagagcaagtgagtggtattaccaaattgaaaaaggctttagtcaacccaacggtgggacagcacaaagtgatccaaaatctttagaagctgtacgaggagacctctttgaaaactctattccaggaggtggtgacggtatgggatatgcttacggccaatgtacttggggagtcgcagcccgtattaaccaactgggtctaaaactcaaaggtaaaaatggtgagaagattccaattatcagtaccatgggcaatggccaagattgggtacgaacagccgcaagtctcggtggagagacagggacaagtccacaagaaggagctatcctttcctttgcgggaggaggacatggcacaccaacagaatacggacatgtggcttttgtggagaaagtctacccagatggttcatttcttatctcagaaaccaactataatggcaatccaaactataccttccgtaaattatctggagtggatagtagcttgagttttgcttatacgacgaaataaaaaaaggatatgctaacaaaatgaataaacgttagcatatcttttgtgtttttttgtcacgattaatcagaatttatagaaatgagaactgttataaaacgaatcatctcatcaagaacatctgcaagtgaaatcccctttgtatagctatatttttttgaataattttgccattgtttattctgaatagggtcagatttgaacctttcgagtagttcaataatcttttcaaaatcgagttctgtttcgcgatatgaaaatgttctctgacaggcatttttcaattgaatgaagtcaatgtcttctttcttcagttttgaaagaatataaacatcgtaaaaatctttgcttctactgttcaagaagttgcgtgaatagatggtttgaagtttctcagctagaattgtttcaattgtataagcaatgattggaaaattgtcttcatcaaaaatagctttatagtcgtatgtaatcggctgaggagttacaacatcacctgtcgcaatatccaaatgaataacttgtttaatattctcaagttggcacaaaatggttgctcgatagccaccatagtcatcactttctttaatagctgtgattgattgaatcacaaaagagataccttcttcggaatctgctaaaatttccttgagttgctgcttaacagtttcttctgagagtgttatttgatggaataaaaagtcaatatcaacagtgctacgagattcaactccgatgacattcgacagtaggaatcctcctttgaagatgtagtgatttgaataagaactttgacttaattttttcaaaatgacttctaggaagtaataggtcatcacggaattaaaggttaaaccagtattctttgatattttatgacagagtgctgttaatttagctttgttcatattagaacctccagagtttgtttaactttttctaaagtattcatttttgtcgcatattcatagagttttgctagattttttttaggatagttcccataagattgaagtgtcttgacaaagagttcggaatcaatcttttctcgatgtattacaaaatcacaaataatacgttcaaaatcatatacccttacgttgtttcccataggagtaggtacagtagtcattcccagctcgctgtattctttagagacaaagtgaatatttagattggctggaggagtattaaagcggtagcctctaggaactgtcacatcaaaatattgtggaatttcatctgtaaactgttgtagatacagtgccgaaatataagagaaaatagccttaggaaaacgatattgaaagaagtagtattcatcatagtctccgttttgggttaggaaaatacctttttcaactcggaagataatgccttctttctctagtcttgtcagatatattgttggaatacctagtgctttacaatctttgtttgtgacaattccattgttcttttctataaaatcgagtagaatctctttttttgacatacggcccccacttgttgcttttacactacaattttacaatatttgtggtataaaagcaacattttcttttccctcaatttattgtgataacaaaatcaagctttcttttttatgatggtattgaacttgggctacgagctgtacaaaaaagagagtttctcctagaacggaaagttcttcgtcaaaactcctatttttgtcgtgctcgcttaacgccctcgtatctaataaggagtgacagtttgttgctacataaaaaagaaaagaaggactatttatgaccaaaacatgtaatcatcactttcttgtcaatcaggataaaggcgagaaacacgtctttcgcaagagtaaaaaatatcgtactttatgttccgttgcccttggaaccatggtgacggctgttgttgcttggggaggagcggttacacatgctgatgaagttacaacatcagttgacaccaccattcaacgaacggaaaatccagccacgaatttaccagaagcacagccaaaccctgtatctgaacaaactgaaagtttagtttcaactggacaatctaacggtgcaattgcagtcaccgtaccacatgatacggtaacacaagcagttgaagaggcaaaggctgaaggtgtttctacggttgaagatagtccaatggatttgggaaatacaagatctgcgtcagagaccagccaacaaatttcaaaagcagaagcagatgcccaaaaccaagttgtggctatcaatgaagttactgaaacctacaaagctgacaaagcgacatacgaatcgaataaagcccgcattgaacaggaaaataaggagctgtcacaggcctacgaaggggccaaccaaactggtaaagagacaaatgcttgggttgataccaaagtcaaagacctaaaatcccagtatgcagatgctgatgtgacagtaaatgaacaagtagtggcatcaggaaatgggacatctgtacttgactatacaaactatggcaaggctgttgaaaccattcaatcaactaacgaacaagctgtagcagattatctaacaaagaaaacaaaggcagatgatattgttgcgaaaaatctggccattcaaaaagaaaatgaagctggacttgctaaggcaaaggcagataatgaagccattgaaaggcgtaatcaggctggtcaagcagctgttgatgctgaaaaccgtgcatgtcaagccgcagtagatcaagcgaatcaggagaaagaacaattagtttcagatcgtgcagccgagattgaagccattacaaaacgtaataaagaaaaagaagctgcagccagaaaagagaatgaagcgattgatgcctataatgccaaagaaatggaacgctatcaacgtgacttagccgagatttcaaaaggtgaagaaggttatatttctgaagcccttgctcaagccctcaatctcaataacggtgagccacaagcacaacatggtgccattactcgaaatcctgatcagattatttcaactggcgatgctatgctgggtggctactcaagaattttggattcaacaggattctttgtctatgacagcttcaaaacaggtgagacccttagttttaactatcaaaatctccaaaatgcacgttttgatggtaaaaagattagtcgtgtgacttacgatattaccaaccttgtatcaccagctggaaccgatgccgtgaaattggttgtgccaaatgatccaaccgaaggttttattgcctatcgaaatgacggaaacggtgattggcgaacagacaagatggagtttcgtgtagttgccaagtatttcttggaagacggttcacaagttaccttctcaaaagaaaagccaggtgtcttcacgcattcttccctcaatcataatgacattggtctagaatatgtcaaagatactactgggaaatttgtgccgattaatggttcaaccgttcaagtgactaatgaaggtctagcacgttctttgggttccaaccgtgcaagtgatttgaatttgcctgaggaatgggataccacatcaagtcgttatgcttataaaggagctattgtctcaacggtcacatcaggcaatacctatacagtcacctttgggcaaggcgatatgccacagaatgttggcttgtcatactggttcgccttaaataccctaccagttgcacgtacagtaacaccgtatagtcccaaacctcatgtaacggtggatcttgaccccgttccagaacctattacggtaacaccagatgtctttactcctaaaacatttacaccagaaaagcctgtaacctttacgccaaagcctttggaagaagtggtgcagcctagtctaactttgaccaaggtaaccttacctgttaaacctattccaaaagaacttccaacgccaccacaagtaccaactgtccattatcatgcgtaccgtttgacgacaactccagagattatgaaagaagtggtcaatagtgaccaagctaatcttcatgagaaaactgtcgcaaaagattcaacggtgatttatcccttaacagttgatgccttatcgcccaatcgtgcccaaacgactagtctcatttttgaggactacttgcctgctggtttatttgataaggaaacaacacaaaaagagaatggaaactatgtccttagctttgatgagactaagaattttgtgaccttaatcgcaaaggaaaacttgttgcaggaggtaaataaagatttaaccaagttttatcaattgaccgctccaaaactctatggttctgttcaaaatgatggggcaacctattccagtagttacaaactccttttgaacaagggtacaaccaatgcttacacagtcacttcaaatgttgtaacggttcgtacaccaggtgatggggagacaaccacactcattgcaccagataaaaacaatgaaaatgcggatggtgtcctcattaatgacacggtcgtagcccttggcacaaccaaccactaccgattgatttgggatttggaccagtataagggagatcgttctgctaaagagacaattgcacgaggtttcttctttgtggacgattacccagaggaagtgcttgatgtggtggaaaatggcacgggtgttacaacccttgatggtcagaaggtatcaggaataacggttaaaaactatgcttcactaaatgaagctcctaaagaccttcaagataaattagctcgtgctaagattacaccgacaggtgcctttcaagtctttttgccggatgacaaccaagccttttatgaccagtatgttcaaactggaatatctttagctttactgaccaaaatgacagttaaagatagtctctatggtcagacaaagacctatgcaaacaaggcttatcaagttgatttcggcaatggctatgaaaccatggaagtgactaacacgcttgtttttccagaacccaagaaacaaaacctcaataaagacaaagtagacatcaatgggaagcccatgctagtgggaagtcaaaatcactatactctctcatgggacttggaccaataccgagggattaaagcagacaactctcagattgcacaaggtttttactttgtggatgattatccagaagaagctttattgccggatgaagcagctattcagtttgtcacatctgatggcaaaacagtttcaggaatcacggtgaaggcttattctcaattatcagaagctcctaaaacgctacaagcagccctttcgaaacaaaaaattcagcctaaaggagcttttcaagttttcatgcctgaggacccacaagccttttttgaatcttatgtgaccaagggggagaatattaccattttcactccgatgacggttttggaaaccatgcataattcagggaagtcttatgaaaacgtggcttatcaggtggactttgggcaagcctatgaaaccaacacggtgaccaattttgtccctaaagtaactccacataagtctaataccaaccaagaaggtatttcaattgatggaaagactgttcttccgaatacggtcaattattacaaaattgtcttggattacagtcaatacaaggacatggtcgtgacggatgatgttcttgccaagggattttacctggtagacgattacccagaagaagcccttaccctaaatcctgatggcattcaagttttggataaggatggcaatcgtgtatctggtatttctgttagcacatacgctagtttgtcagaagctccgaaagttgttcaagatgccatggctaaacgtcagtttacacctaaaggagccattcaagttcttagtagcgatgatccaaaagccttttatgatacctatgtgaagactggtcaaaccttagttgtcacgcttccgatgacggtaaaaaatgagttgaccaaaacaggtggtcagtacgaaaatacagcctatcagattgattttggcttggcctatgtcacggaaacagtggtcaataatgttcccaaactagacccacaaaaagatgtggtgattgatttgtctcataaagatgagagccttgacgggaaagaagtggccttgcatcaaacctttaactatcgtttggttggagctttgattccaagcaatcgtgcgacagatttatttgaatatggttttgaagataactatgatgaaaagcatgatgagtacaatggtgtttatcgcagctatctgatgacggatgtcaccctcaaagacggttctgtcttaaaagaaggaacggaagtcacgaaatataccttgcaacaggtggatacagaaaatggcctagtgtcaatttcatttgataaatccttcttagagtatatctctgatgattcagcttttcaggcagatgtttacctgcagatgaaacggattgcggctggtcaggtggagaatacctatctccatacagtgaatggctatgtcatcagttcaaatacagttgtaacacatacacctcaacctgaagaaccaagtccaaatcaacccactccacctcaaccaccgattgagtctcttgaaccacctgttccagcaagcgttttgccaaatacaggggaacaggaatcccttttgggcttgattagagctggtattctacttggtacggcttatggactgaagaaaaaggaggagaagtagatgaatccaaaaactatttatgaaaaggattcagaccaagatggtttgacagatgctcaggaactagctttgggaaccaatccgcagtctgttgacacagatggtgatggtcaagctgatttagaagagctacactctggatattcaccactagtcccacaaaaggagttgtgcgatgacttggaactttgacaccatgaaagaggctttgtctgaaatggagaaggtcgattaccaagagtttatcaaagcctttctctctttggaattaagtatttctgatagaacaatcctcaatcaggtttatcaagattatatggatgaggatgacctatccttgattagtgatgagctacgagttaaagttgatagttatcaggatgaagtacaggcagatatgactgacattctggaaaagttgtaccgaacaggtgaaggctctagttttattatggatttaatgtcctcaaatagtctctcagacaccttggaacagtatgaggttttggatagtgacgattattcaccacttagccttgaaaccttacaggccataattcaacaggaattggctatttccagtcaggattattttggagatttggttcaccttgccttgcaaaaagatttactagaccagaaaagtcatttcttacaacactatgtggcaactgtaatggaaggtattccacaagaaagagaccaacgagccttggtcctagattaacacaaagggctgagaaaaactctcagcccttttaaaatgggagggaaaatgaatcaagaagtcttactacaaatgatgagagccaccattcctcgtgatagagccctgcttgaggcatttttatattaccaagcagagcattttgatgaggagtgggatagtcttattcgtcagtttttaaccaataggaaagaaattaagaaatctgttcaggtacttcactttgagacagatgtttcagcttttgtccaggctagtccttatgatactgctcatgatctattgacctatacacaagttttcggccaaagtggtctccaaaaactagacaaactatcgccgactgaaaaaaacttggtgatagaagtggccttgttcaatctggccactcgttttcaattattggattccaatggacactaccaaaccatatcgccggattcactcttacaaaagagtaggggagctaatttggtcaatgtgtatcgtgtggctaataatttagcggatcggatcagccgagatattgagcagtttctcttgacttacgagtctgagcttgaaacaggagttgatgaaacagttctagaaaatgaagcaactgttgatgagcacaaaacaagtgttcatcaagcaatatcttttagagaagatggctctttgattattgctagtttggatgtagatttgtctcaactagatgttcaaacaggaaaaaccagtcatctgcctgcctatgaagagttatctttacgacgtaaatttgagatactaacatattttgaccaaattcgaaatgaacgttccaaagtcccaagttttaaacgaggtgattttgacactgaaatggaaatgacaccaatctttgagggcgaggagttactcacctatctagaagctgatggtagtccctatgagttgaaaagaacattgactacagtcgaagaaaaggaattagaaaaaattggacaagccattagggcagaaaatcaagaaaaattgattcaggtagggattgatttatctcagtttgacccagaccgaatcggtattttattggatgcagcaggtcgttttcgtttaaaaaatgcagaccttgctttactaggtggttatcccaaagcctcggtaactcaactagcccttgcgacagaactactccaaatgggactaagtcatgaaaaggttgaatttttctttggtagccagctttcccttgaagagctgcgacaagttgcctacgcctttttacaccaagaactcagtagagaagatgcggaacattttgaaaaggataagattaatcagccagatttaacccttagagattggaagagcaagctagagaaagttgagggaaaagaagtagttgatgaagaatttgcggaaaatccactagtccagagagtattggacacttatcctctggggtcattggtttcctataagggacaggactttgaggtcatgtcggtcagcgatgctcgattgaacggtttgattcggattgagttagtcaatgatttttcggatatcattgaacaaaatccagttctttatgtgaggatctgggaagaagtcagtcaggcacttcatcagccaaaggcagaaccacaaacagagttagaagaagcggaccaagaactaaatcttttttcatttctgaaagatgagccagttcagagtattggactattggaacccgatggttctgaaaaaggtcaaaacgatactgatcttgaagaaacagatagtcaaattcctgaagaggtagtcgtcgaaacaattccagagattccagtaacggacttttattttccagaagatatgacggacttttatccaaagactgctagagataaggttgagacaaacattgcggctattcgtttggtaaaaaaactagaagtagagcaccgcaatgctttaccaagtgaacaagaactccttgccaagtatgtaggctggggtggactagccaatgaattttttgatgactataatccaaaattttctaaggaacgagaagaactgaaaagcctagtcacagataaagagtattcggatatgaaacagtcctctctgacagcctattatacagaccctgcccttatccgtcagatgtgggataagttggaaagtgatggctttacaggtggcaaaatcctagatccttccatgggaacaggaaatttttttgcggctatgcccaaacacttaagagaaaagagtgagttatatggtgtggaattagatactattacaggagctattgccaaacaccttcatccaaatagtcacattgaaattaagggctttgagacggtggcttttaacgacaatagttttgatttggtgatttcaaatgtgccatttgccaatatacgaattgcggataacaggtacgataggccttacatgattcatgactactttgtcaaaaagtcacttgatttggttcatgatggtggacaagtagcgattatctcttccacaggaactatggataagcgaacagaaaatattttacaagatattcgtgagacaactaaatttcttggtggggttcgactgcctgattctgcctttaaggccattgcaggaacgagtgtcacaacggatatgttattcttccagaaacacttagacaagggatatgtggcagatgacttagccttttcaggttctattcgctatgagaaggatagtcgcatttggctcaatccttactttgatggagaatacaatggccaagtgctaggaacctacgaggtcagaaattttaacggcggaacactttctgttaaggggactagtgatgacttgattgcaagtgttgaaacagctctacatcaagtaaaagccccaagaaagattgatagaaatgaggtcatcattaatccaaatgtgttgaccaaacaagtcattgatacctccattccagctgaaatgagggagaatctaggtcagtacagttttggttatcagggttctacagtttactatcgagataataaaggcattcgagtcggaaccaagacggaagaaatcagttactatgtcgatgaagagggcaactttaaagcatgggacaccaaacattctcaaaaacagattgatagatttaatagcttagaggtgactgatagcacagctctggatgtttatgtgaccgatgatgcagctaaacgcggacaatttaagggttattataaaaagacagttttctatgaagctccattgtctgataaagaagtggcacgaatcaaaggaatggtcgatattcgcaatgcttaccaagaggtcattgccattcaacgccattatgactatgataagaagacctttaaccacttgttaggtaaactcaatcgtacctatgatagctttgtcaaacgctatgggtatttgaatagtgctgtgaaccgcaatctttttgatagtgatgataagtattcgcttcttgctagtttggaagatgaaagtctagatccaagtggaaagtctgttatctatactaaatcccttgcctttgagaaggctctagtgcgtcctgaaaaagaggttaaaaaagtacatactgcccttgatgctttaaattcgagcttggctgacggacgaggcgttgattttgcttatatgatgtctatctatcaggttgaatcgaagatgaccttgattgaggagttaggtgacctcattatgcctgatcctgagaagtatttgaatggagaattgacctatgtttctcgccaagactttctgtcaggggatgtcgtcactaagttagaagtggtagatatattcgtcaaacaagacaatcaggactttaactggccgcattatgcaggacttctagaagctatcaaaccagcacgtattactttggcagacattgattatcgaatcggttcacgctggattcctttgtctgtttacggaaaatttgcccaagaaacctttatggggaaagtctatgaactgtcagaccaagaagtagcaatagtccttgaagtcagtcccattgacggggttatcacttaccaatctaagtttgcctacacctattccaacgcaacggataggagtttaggtgtccctgcttcacgctatgatagtggtcaaaaaatcttcgaaaatctcctgaattccaatcaaccaacgatcacaaaacaaattgttgaaggggataagaaaaagaatgtgacggatgtagagaaaacaacagtcctgcgtgccaaggaaacacacctacaggaactctttcaagattttgtagcaaggtatccagaagtccaacagatgattgaagacacctataacagtctctataatcgtacagtatcaaagacctatgatggtagccatttaaccattgatggacttgctcagaatatctccttacgtcctcaccaaaagaatgccattcaacgaattgtggaggaaaaacgtgccctactagcccacgaggtagggtctggaaaaaccttgaccatgcttggagctggtttcaaattgaaagaattggggatggtacataaaccactttatgtggtgccgtctagtctgactgcacagtttggtcaagaaatcatgaaattcttcccaaccaagaaagtctatgtgaccactaagaaagactttgccaaagctaaacgcaagcagtttgtgtcccgtattattacaggggactatgatgccattgtcattggggattcacaatttgagaagataccgatgagtcgtgaaaaacaggtcacctatatcaatgacaaactcgagcaactcagagaaatcaagctaggaagtgacagtgattataccgtaaaagaagcagagcgttcaattaagggattagaacaccaactagaagaactccaaaaactagagcgggatacctttatcgaatttgaaaaccttggtattgattttctttttgtggatgaggctcatcactttaagaatatccgtccaatcactggacttgggaatgtcgcgggaatcaccaataccacttcaaaaaagaacgtagacatggagatgaaggtgagacaagttcaggcagagcatggagatagaaatgtcgtttttgcgacaggaacaccagtttctaactctattagtgaacttttcaccatgatgaattacattcaacccgatgtcttggaacgataccaggtatcaaattttgactcctgggttggggcttttgggaatatcgaaaactccatggaactagcaccgacaggtgataagtaccaacctaagaaacggttcaagaaatttgtcaatttgcctgaactcatgcgaatctataaggaaactgccgatattcagacctcagacatgcttgatttaccagtaccagaagctaagattgttgcggtggaaagcgagttaacgcaagctcagaaatactatttagaagagctggtaaagcgttcagacgctatcaagtcaggtagtgttgatccaagtagagataacatgcttaagataacaggtgaagccagaaaactagctattgatatgcggttgattgaccctgcttataccttatcggataatcagaaaatccttcaagtagtggataatgtcgagcgaatctaccgtgaaggagctgaatacaaagccactcagatgattttctctgatattggaacccctaaaagtaaggaagaaggctttgatgtctacaatgaactgaaagctttgctggttgatcgagggattccaaaagaagaaattgcctttgtccatgatgccaatactgatgaaaagaaaaactcactgtctagaaaggtcaatagcggagaagtacggattctcatggcttcgactgaaaaaggtggaacgggtctaaacgtgcaatcacgcatgaaagctgttcaccacttagatgttccatggcgtccctcagacattgtccagcgaaatggacgactaattcgacaaggaaacatgcaccaggaggtagatatttatcactatattactaaagggagcttcgacaattacctctggcagacacaggagaataagctcaaatacattacgcagataatgacctcaaaagatcctgtgagatcagctgaagacattgatgaacaaaccatgactgcttctgactttaaggctttagccactggtaatccctaccttaagctcaaaatggaattggaaaatgaactgacagttttagaaaatcaaaagcgagccttcaatcgctcaaaagatgaataccgccacacagtttcctattgcgagaagcacctccctatcatgaaaaaacggttgagtcaatatgataaggatattgcccgatctttggcaacaaagtcgcaagattttgtcatgcgatttgacaatcaagcaatggataatcgtgctgaagctggggactatctgcgaaaactcatcacctataaccgctcagaaactaaggaagttcggactctgaccaactttagaggatttgatttgaaaatgacaacacgtggtcctagtgagcccttgccagagactgtctctttaatgattgttggtgacaaccactatactgttaccttagatttgaaatcagacgttggaactattcaacggattagcaatgccattgaccatatcatggatgaccaagaaaagacagaagggatggtaaaggatttaaaagataagcttcaagtagccaaagttgaagttgagaaaatttttcccaaggaagaggaatatcagcttgtaaaggctaagtatgatgttttggctcccttggttgaaaaagaagcagaaattgaggaaatagatacagctttggccaagtttagtgaagatacaacaccccaaaagaagcaacaaatagcactcgagatataagaaaaaaggctgacaaataattttgaacgcggtaaaatgaagtcaagaaatcacaaaaggagaatagatcatgacccaaacactagaagaaatgcgttatcaacttgaagaatggttggcacaaggctacacaagtccagaagatagggcaaactaccaaaacttaaaggaacagtatgaagatgaaactcttgattatagcttttcaaggcgtgaaatcactggacagctggaactcatcatcacaagtcgtgagaatgattttccagacctagatgaggtggtgaaggcggaataccttgatttggttgcccaacttgatgatttagacaagagacaggctgactactatcgcaagcaattagcctagaaagaggtgtaagatgttggagcaaattctacaaagccttttgattatcgcagcaataggactgatgttgtttgtcctttatcggattgtgaaagtttcaggtgctttatttcttatcggactcatcagtggtttgatttttatagaagtttatggaatttacctattctttacggaaaggtatctctatacagaagatttagccaccaatggtatttggagttttactggtttttatattgcctttaataactttctcttgtttataattactataaaaatcttaaaaatagagcaacctaaatcttaggattatcaggaatgtgtgaattggtattcctagtgattggtttaagatattatttgtttttttcaatacacatagaagaaagtcaatgtgaactgatatttcaagataaagtacagttcagaatagatgaacttgagaagcaaaaagcaaaatttaataagaggaataaagagaactaattatttagtttgcaggtttattttctcgtttcgttatttttaggatttgtaccatagagttttgccttttttactcatgttttacatcactaatcgatttataataatcttatagggaaacatagttacaaatatttaattattcaaggaggaatttataatgaataatattaaaccagaaatctatgttcaaactgcaactgatcaagaaattacattattaattggtggtgctggtagtgggtttgttaagactctaactaaagactgtccaggctttttatcaaatgtttgcgtaaatattggattcatctctggatgcaaaaattgttaaaataaaaattagtttgtaactatgtatgtcctatcgccataattgataggacacttccttttattttactagaggtaacatctatgacaattaatattacagaggaaaaaagttttgatatagtaatttcaaaggtaatagaggaattttctttctccacttcaaaatttagtgaagtgtgctctttttctgatagcattattaatagttttaaggaagagtttagaaaaatactgcttcctgtattggtccaagaaataaatttatttaggatagggaaaggaaccaattcaaaatatagtgaatacaatgaattttgtaaaaatattcttaataacggtttgtattttcttgataaatatccagttttaaaaaggagattgaatcttttaaaagaaaattatgagattagcattgaatgttttttgaaaaatttatccaagaattataatgaaattatagaaaattttgatctaaggcaagaaaatataagtgtaaaaatattatcaatggtgggagacaatcacggtagtaataggaatatcagctttgaacttcaaaataaatcatttttttataagacttcaggatacactttgtatccaattttgaatgaattaaattgtagagtattcgattctaagtatttcaaatttcctgatacattcattggtagcaattttatgatccaagagaagattgacaatttgtcctgccatgataaaagtcaaatccattcattttatagaaatatgggagtacttttggcatttacgtatgtgttgaatggaaatgatatgcacaatgaaaatattattgcttgtaaagaacatccctatgtgattgattttgaaactttaataaatcctgtgtcacaaatagagggaagaatttcacaaagcatcttttcaactggcttactcccaatgaaatatagacgaaattttgatggaatatttgactgtagcagtataggtcaagtaaatatagttgtaaaaaaagtttttgaagaaattaacccgtttagttcggaattacgattggaattgacagaacaccaatttaatgatatcaaagaatttttacctttactccatgaatctcatatttctgctaataaatttttaggtgatattgaatctggattcatatttggttattcaagattgagaagttgttataagattataaaaacagtagtcttaaggtataaaaatgccttactatgtagattggtcttaagaaatacatctgtatattcagcacttattgatcgaattactgtgccagatttattgatggatgagctaaaaacaagaaatgttttaacaagcttcttaaaagaaataccagtaatcagtagtcatgaaaaaaagtacattatgcttgaagttgagcaattagttaatggtgaggttccattgtttacatttggtaattttggaaatgtttcaagaaatgatacaaaataccttatgagtctttacgattctgtaaattttaagttaaattgtatgtccgatgaagatttacaatatcaattaaatctccttagagtttgtttgaatattgataattacaatttttctaaattgagaaataatcatcttgaattagatgttaaaaaattagtaagggaatcaatatttaatgtcacacggccttatgcactgactcttcagaaagattctagtggtaattacatatatggagagtctaatttgggactatatgaaggaaagttgggattgctattatcttatgatgaattggaagaaatatttgatttaaaagttattgaacaagaaattataaattcaaaagatataggcttaattaatggctatgcttcattattgctttataaaaatttaaataactctttaaataaagatgattatttacttcatgatatagactttactgaatttgatataattgatggtttgggtggattaattttacaaagctatttaatatttttgaaaaaaccagaattagtagatgtagttgaattgaagaagttaggagaagtttttttagaattaacaaaaaatcaggaacagtttaaagtaggatttgctcatggatttaccggtataaaggttatttataaaatatgttctcttattttaccagataattcagagtttatgaatagatttgagtactttgagagtcaggataatgtttcattatatcctagctcgggatggtgcaacggattgacaggttatcttgtttctgaatatattttatataaaataagtaacgactctagatatcttgataaagtattatataatattgattatcttatgaataaattatgtgagactgaggaatattgtttgtgccacggatttttaggaggattggatttcttgcaagtattgaatcaggctaatttgctttctaacgagcataagaagagactggaaatattggagaaaactttaatttttaaatccaatcacaataatattctaaaaaaagatatttcgctatttacaggaatctcaggatttctctattatctcaaacgaaaaaaaacacttagagggtctgttattactctagggttctaagggagatgccaatgaaaaaaataagagaaaagctacaatcccaacaaaatgattgtggaccatgctgtttagcaatgattttggactatttaggaagagatgttgagttatcagaactatataaatataaaaataccgctgttggatggagcatgggagatataaagaacgttgcaaaacaattcggtgtagacgctactgtgtttaggatagttaatatgaaagggatgtcatcaatactacttccagcaatactttactgggatttctcacattttgtagtgctagaaaagtacaacgagttagaggtaactatacttgaccctaattatggtcgaaaaactcttaacaaagatacatttataaaattcttttcaggatactgtatggagctaagaccaaatcaatcctttacaaagcgaaagtcatccctaagagataaagtatctaagatcagaaacacaaaaatttggaatttgaaatttggaaaagtctatcctactgttttggtgggttacaatatattattattaattatacctgttttaataagtaaattaataaatgaattatcaacaagagacattaatataaatcaagtaattattttaataggtgttatatggttaacaactggtttgaactatattttagaaaaatttcggaatcaaaaaacaataattcttgaagaaaaatcaacagttaatatttacgagaagttatttagtctaactgaatctgaaatatgcagataccatagtggcgacatcatgtcacgaatatcggttaatcctcaaatctgtagatttttggcagttgaagtacctaatatgattatctcaataatgcttattttctttgcaagtttatacttaatattaaacactggaatctatgcttgtatgttgattttgctacttttactaattgctatcattaactgtatattcatcactattttaatgaaattatccaaaaatgaaagttataaacggtctatacttcgaagtgttgctaatgatggtattctatcttataattttttaataggatcaggaattacctatcgttatctaaaaaaaatcaaaagtagtttatcagattatgtacagtcgcagattgatagaggaattgtagaagctaaatcaataacattccagcaagccacttcgacattcttttctcttctaatttctattatgagcctagttatagtgttattgaatcctgaaagaagtggggaaattgccttaatcagttctatggctatgatattgtattctcccacgatgggaattgtaagtagcctaattaacatggctaatctgtatccaaatattgaaagacttgtagacttggtaggagaagaacaaattcctaaaacctttacgaagattcaaaacggcacagttaatgttgatgggttatcatatagatacgacgacgcatcaacatatcttttcaaaaatctttcgttctctcttaaggaaggagatactctttttataactggaaaatcaggtaccggcaaaacctccttagtaaaaattttactaggtttacaaagtattagtggggaaggtaatgaaggaaatataattattggaggagttaacttactagaccaaaacctagatttaagaagagatatttgctatatatcacatccgtcaattctttttaaaggcactctgaaatcaaatttgaaattattctgtccaaactatactattgaggaactgatgttagcaattgataaagcaaatctttcagatatatttcctcatgctttaaatattgaaaatttatttatattggaaaatggtactaatttttcaactggtcaacgtcaaagaataggaatgctaagattatttttgcaagaatataaggttattattttagatgagccaacaagtaatatggattctgaaaacgcaaaagctatcatggcagctattaatgctcttgaatcaaccaaaattattattacacatgataatgaattaattcaacctggatcaaaacaattagaattaggagtgggagaaaatggatatacatttaaatcatataagtaagaaatatggtaatcgaatcattttaaacgatgtcactattaggattccttcagggaaaatttatgggtttattggtgcaaatggagctggtaaaactacaacgatgaaaatccttactggattattacctgctacaaaaggtaacatatactttgacggtgtaagtcttaacgacttaaaaaatcaggagtcaataataggagcttttattagtacaccaagttactataaaaacttaacagcttatgaaaacttagctattatacaagaagttctcaaaaaaccgcaagaagaaattgatcgagttttgaatctggtgggcctaagtgaggttcgcaataaagttgtgtcttcgttctcatttggtatgaaacaacgtcttggtttagcatttgcttttttaaataatccagatgtattggtattagatgaacctacgaatggattagatccgaaaggaattgttgaaatcagagaactcttatacagactagctaaagaagaaggaaaaacgatttttatatcaagtcaccatattagtgaaatagaaacgattgcagatatggtcggtataatacatgacggtgagcttatttttgagggaacactctctgaactttataataaaagggacagctcttatctttttgaagtcgcaaatccagatgtttttgaacatattttgagagttcgagatatctcttttattcgaaaagaatcgcattttgaaataaattcttacaaaaaagatataccaaatttaataaaattaattgtacaagaaggaatcgatattttggaggtttcaccaaataaaaacctagaaagaatcttcttagacttaacgaaaggagataaagtgtatggtcacattgataaaaaatgaaataaagaggtttgtaggaaccccaacagccttgttattgcttggcattactttttgtttgcaaatgctaggaacggtttttatcattaaatcattagatgtgcaagttggggacatagcacaaacaccttttcagaagtctatgctaatttttttaggtttttctggattggtgagtttagtggtaaatggtattactaacttttttgttacttcaagcgagcacaataaccatacttgggaattgttgcttttggggatagggaagaaaaataaaattctcttttcaaagtattttacagtcattttatcttttattttttatcaattactgtcaactattttctttgttatagttacacagacctatttatcgttaacgattgattggtctacggtattcttagtgttatcatcaacaatatttttcagcttactcacgatcacacttcagttttgtactcacctgctagaaaaaaatagtttatctgctctaagcagtgttattattcttattctgttgcaaacacttttcgcctctaatgctatatttatttatgtattcccgataaatggtatttcttatctgataacttctagtattctcaatccaattataattttagtaatcactttagagaatattatatggagtggtgctgttcttttagtagtttttaaaaagtttcatctttaggagggaattatgtggaaattagaattattaaagtataagagaacctatttagctcccgtattcatagggataggtatgtttttagtcatttttcaaagctacgctggatatacgattaaagaaattccatcgaaggttttatttgtgacaggtctagatttatatagtaattttttactaccagtgatgattcctgtatttcttatcatttcattgcaccgtgaagttgagaatactgcttttcaaaacttaacaataaaaggaatatctagtcaacagataaagtggtctatagtgaaattttattggatagtaatgccaattttatttctttgtcactttttgattccactgatttttatttccttgagaggagaaagtgcccttaatgtgattattgataatagtttatttttactgttatcaattttaagtattattgcactcatcaatattagtctactgatacatcagtcaagcgggaattatgtattacctattttaacagctattgtaggagtcgttgttggacgtttccctctgggagaaatcacatggattgttaacccctactcctatttaagttatttagcaaattttaaatcatttactgtaacccactatttagcgttttttctagtcttttctttgtcttttattggtataatattatgtaagaaaaaaatagatttgagtaagacatgagaagaaatttgttaagtacagcgtattatgctttgtttattacaggactaatctatttgtccgctaaatgctttctaaataacatcgaaaatatcttttttttgcttgctttaaatgtattattgttattggattttattttgcacagagcaatatctactaggtctaagattagcaaatttattatttggttaatcagactttgtcagtgtcactcaattttatttgtcataggagattccgcatttcctccatggatacagtatatgattggcattataatctattcttcgttaatatctatatcgctcagcttgttttttcaaaagttacagacttctttttatctatttgtgatgatagtaggacttctatcgagtgtaagcttactttttccaagattgactagtattgccttttcactttatttggtagttacgttcgcgatggctctcttaattgttcttctaaggaacaagcaaacgttttatagaaatgattccaatagattttattggctaatgttattaatcttttacctcatgatttgtttcagttctattggtttcattttttttaattcatcagctttattttctgggttctggttattttattttttagctctaattacaggattaatactcatcttgttttatcataatgatgcagtatttaaacaaaaagaacaattattaacacttatttttctgattttattatatattgttctgtttattctaattttaaagagaacgagtctagttgttcttgaaacggtaacaattattttatattttcagtactttttccagttagttggagaaagttatagactaataactggaagtaggcaatctgaacatgtagggatcaattcaattttgaaagaagaagctttaaaacaagagtttgcgaatttcctgcatgataatattcttcaagatatcaatgctttaattcaactatctcgtttagataatccaagtgtatcagttaaaattattgaagaaagactagagtatctcaatacttttgttcgtgagcgaatgaatcagtatagtcctcaattactaaaaggcttatctttgtatgataactatcgtatgatgctaaaagcatttgaaaaacgttaccctaaaatgaatatttctctgaacttttattcagcaaaaaacatagctatctatcctccttatgattttttaatatatagatggttaagagagctcgttaataacgcttataagtattccaatgctcaaacggttactgtaaaattggctagagatgggcgggaactctctttatcagttatggataatggctattatcaaaagttaaaaccatttaaacatggacacggattattagtcttggaagaacaagtaaagtcagtaggggggactattacttttgagcaatcggactctaacggattaagagttgatatcaaattgatgatggagggagagaaggcaattgaaaattttattaatagatgatcatatcctgtttgcagagagtttagccttaactatacatcagtatcagccggatatccacattgatatattgaatgatgaagatgaatttaaaagcattttttcaaaagtaattgagtaccaagtgatactattagacattaacttagacaataaatttagtacggatggctttggaatagctcaagatattttagaggaatatcctgattccaagatagcattattgacgggatttgatttacctgtatatgaatatcaagctcagaagataggagtaaaggcatttgttcctaaaaatataagttccaaaagattaattcagattcttaaagatatttcctatgggaaaaattattttccaaatcagaattattttatagatgaattgacagaacgcgaaaaagaaatacttatacatttgggaaatggcgaaaaacgaaaagacattgctaaatccctttacatttctgatagaacgctaacgaatcatattcaaaatatattggaaaaactcgaagttgattcaacgttaaaagctgttataaaagctcagaagttaggatatattaaataattaccctttttactcatgattttgtattgtaaattagatatactggtgtaaaggaggttcaaattcatgaaaaaagtaataccgtatgcgttattaactctatcaatggtttttctattcaatgctatccgaatatatcttgagagtaggtctctgtatgttgtcgatgttttgttattcatagtctttctagtatgtagtttaaaatttagaaagcaataatgtagaataaatacaaagatatactatcttccagattgaagaaaaagtccattttggacaattttcttcaatcttttttcttaatgatgaaaattaaaaactcttagaaacaccgaaatatcaacatttctaagagtttaatgatttgttatctttcgcaaactttttctattttttatatttttaggagtttgtctacgttctgaaagatatactatcttctacatttgataagttataaattattcagatggtcataatttggttgttaaaaagacccattgccagctatagcaggagacatataatctataggggagaggagcagattgattctatctttttgatgttaagttagtttagaattttgtttctgaagtttgtaacttgtcgaaaactgagtgactgacgtttgatatccttgatgagcttatcgattgcgttaaattagtgttagaatagggaaattcagtcagatagcagttctaactactgttgtacgacagaggttgagaaagggagtctttaggtagtggaagtgtaggtgagtttttcggtgtagtattgagtaatctttatgcagatgggttgtgagatttggtgtttttcgtgatgagagacacttcagtgactgtcatctgtttacaggatttacactgaagaagacatctttgggttttaacatagttggcatctctttgatattcagaatagatactgtagatttatgttggaaataacatctgactaaggcagtttggagctgggtaatcaagttcacgctgaatagtgatatgcgtgttgacttgaaaaagtagtctcgatttgatgttcttattttctaattcgaagagtactgttgtattctaaaattgttccataagcacttcctaatgatggttagggaggtcttgtggaacttttttgtatactcaaaaaactatagcctctgtatagattgttactacagaaattatatgataagtcttttattgtagtatatcttaaaaatatttagctcaaattattctacaaagtagttaatccggtaaataaagttatttggctcagttttaaagtaattggagtgaaaattttaaattttcttaaagagtcattatagctatattttcttgaaaagatgtttgagcaccaatttttgaaaattagttcagtagaatttggaatagagctggctacttcaaaacgcaacgaactataattcaacatctttatctattgaataggttctagtttgctatttttgatttttacagagtataatttgtagttaatcatatttttcttgtgataaaatttccaacttaatcttgatatgatgaggctatcactaataaaggagtaagccatgttaaataaagtgaaagctcgatttctgattggagtaggaggtttaatcgcagtcagttttatggtcatgattggctacacgattggctcacaaaccatttcaaagcaaacagagaaccaaatacgagcagaagccaataaacttgtgtcaaagaaaaagcaggaggaaagagcgacagtcctatcagatgagcttgtcaaagaatttctcactcaatatttcaccaaggttcagctgggtgaaaataacgctcgtatcaagccctacatgacggattcggcattttcagaagaggaagcaaatcagaatatagcgatcaatcaagtttataaagattatatgcttgactaccgatttgaatcagcgagtatctatgtcaatacagaaagcaatgttgcacttgcggaagttacctatcaagtgacctatgtttctgatttgagtgagcaacaacagcgaaccactcagacagaaatcaagacggttatgttatcctactccaaagtttccgacaagctcctggttaatcagttgaccatttggaatgggaaactggaggacatgaaagaagccacaaatggtgctaattccagcataccaacgatccaaggaactacaacaagtgagaacaactagcaggagacagtcttctgctttttttgatagggagacaagatgacacgaattaaaatagtaaaacaaaaggccattttagatgtggctgaaagtttgggttattccttcagacgtttatcaggacacatttatgaacacccagaccatgattcctttcggatttttgccgataccaatactttcaaatggttttcaagagatatacaaggagatgtaattgactttgttcaattagtggcaggtgtttctttcaaagaggctgtgtcttatcttgaaactggaggctttgaacaagctaaggtgatagaagaaacttatcaatcgtttcaatattatttgcatgaagaaccctttcagaaatcacgtttttacttaaaagatatccgtggcctaagtaatcagactatcaaatcctttggtagacaaggattgcttgctcaagctacttatcaatcggagcccgtattagtgttaaaaagctatgaccacaacgggaccttacaggccgcaagccttcaaggtctcgtcaaaaatgaagaaaaacacgatcgaggttatctaaaaaaaatcatgaaaggatctcatggctatgtcggtattagtttcgatattgggaatcctaagcgactcattttttgtgaatcagttatcgatatgatgagttattatcagctttaccaaaagcaattatccgatgttcgcctgatttcaatggaaggtttaaaactttctgtgattgcttatcagaccttgcgtctagcagcagaggaacaggggaaattggcatttctagatacagtaaaaccaagcaggcttagccattatcttcaggcaatacaagagacgacaaccttttttcaaactcattcaaacgtcataacaatggctgttgataacgatgatgcaggaagagaattttgtcagaaactgtcagataaaggacttccgatttctcaagatttaccaccattgcagggccttgaaacaaagtcagattggaatgatattgtgaaacagcagagtgaactatccttaagtgattatttccaaacagcccaagcacaagtcaataagtatcatcctccacctaaatgggggcatgctttagaattgtgataacaaaatcaagtccgctatcataatctagaatgtgacaaggaggacaccttatgagtgtgattgaacgtctggctgaaaaagtagctagacaagaagaaaaggtctcacgtgagacggagaaattggaaaactatcgagaccaactacaaacagctatgtacagtacctttatcaaacggcaacaatctagtcagttgtcatttcatgaagcactagagcaagcctttggtaaagaaaccacactacacccagattacagaaatgaggatacagaatgagtaaaacatggaattttgatcagccactagatgatgtgaaaccaacatcatcccacgaagaacgagctaaaatcgcagcacttttccataaacaggatgaaacactaattgaagaagtagattatgtggctacttttgaacagaaacaaaaggagtcagaatctaaagatgtacagacgcctgaattaaaagttaaacaaaatcagccaaagaaggtaaatattacaagtgactacaaacagtacttgacagacaccattgcacaaaacaacaaggatatttccgcctgtcagaagcaaattgaagaacttcatcaattgattgatgaaaagaatatccaaaataaaaagttgcgggctatttcagtagccattgatgatttataaatcaggtagtcctctgcgggctacctttttacgatttaaggagaagaaaatgaaattctttcaaaagaaaaaacagaaccaggatcaatttaaacgactgattcatcgactatctgagatgtcagatactgaactaaccaaagtagaacaactcctagatgtggtttttgatacagattttaagccagatacgaacgttgaattatctcaaagcgttataactgatgaggaacaaagtcttgataagtcgatccaagaagcgaaaaacaaactcaacacagaacaattagagaaacgaatagaacaatttaaacagtcgaataaacctaaaaacggataacgcaccaaaaatggtgcgttatccgttttttatggtataatgaaagcaaatatgaggaggtctttatctatgattggagacaacatcaagtcactacgtcggacacacgatttaacacagccagaatttgcgaaaatggttgggatttcacgcaatagcttaagtcgttatgaaaatggaaccagcaccgtatcaacagaactcatcgaccgcatttgtcaaaaatttaacgtttcttatgtcgatattgtaggagaggacaagatgttaacacctgttgaagattaccaactgactttaaaagtagaagtgataaaagaacgtggagcagccattttatcacagctttatagataccaagatagtcaagacatcgcctttgatgatgaatcgaatccttggattttgatgagtgatgacttggccgaattgattaacacgaaaatttaccttgtcgataccttcgatgaaatagagcgctacaatggctatttagacggtatcgagcggatgttagacatggtgcatcattgggtggtggcataatgagattggaagaatttagtgaggaagagtttcagaaggccttgcagcgaactattagggcccaaagtcgtgggaagacgattccaaatcaaccgagagccattttacttggaggtcagagcggagcaggtaagacgactattcatcgaattaaacaaaaagaatttcaaggtaatatcattatcatcgatggtgatagctaccgttctcagcatcccaattacttagccttgcaagataagtatggcaaggacagcgtggactataccaagggatttgcaggaaaaatggtagagcatctggttgacgaactcagcacgcagggttatcatttgttaattgaagggactttgcgtaccactcaagttcctcgtcagactgctcaattattatcttctaaaggttaccaagtttccttagcagtgattggcaccaaaccagagctttcttatctcagcaccttgattcgttacgaagaactttatgccatcgatcccaatcaggctagagcaacaccaaaagaacaccatgatgggattgtagagaacttggttgataacctaaaggagcttgaaagcgataaactctttgaccaaattcagatttatcaaagagatcgaacttgtatctacgattctgaaactgatgaaggctccgcagcagaggttcttcaaaactgtcttttcgggaagtggagtaaggttgaggaagagatgttgaaggtaggacaggaacggttgggggaattagtttcagctaatggagagaagaaatgaaattaacaaaagtaattattaataattttagatcattcggtgatagtcaagttattggatttaacgatcaaactgtactgattggaaataatagttcgggaaaaacaactgtattacaagcattgagtaagctgttttcggataaacaaaatgatagaataataaggagttgggaaatgcttcaggtagtatgttaagtaggctggtaaatagtataaattggaccgacgatgagataaaagaaattacaaacaaaattgatgagttgactaatacctttttatctgaaagcggagcattaacccaaatcaaccaagaaattcagaaatcgtggaaactatatcatgaagacaaccgtttttctcaagcagagttgactattaattcttctgagatggcgggagcccttagacaaattgctttaaaattttcaccgacaaccacagaagaagcgtttacagtttcagatttaggagatgggctgagatctattttttatttttcacttgttgattcgatacttgatattgagttaaaaattattaaagaccgtgaagaaaatcctgataatccaagatttaaattgataccaccaatactaaccatcttagcaatagaagagccggaaaatcatattgcaccacatcacattggtaaactggtgaaacgatttaaacagttgagtaataatgacaactctcaagtagttttaacttcacactctccagctattgtaaaaagaattgagccagaagatttgagatatttaagaattgaaaataatgatagagtgcttcaaactattgtttctggtatacagctacctcaagccattgatgtatcttataaatacattaagggagcaattcaagcttatccggaactatattttgcgaagttagtagtactaggtagtgaggaactgttattaccaaagttttttgatttacttggaaaagagattgatagctctcagatttcaattgtccctctagggggcagacatgttaactatttttggaaattattgaattcactcaaaattccttacattaccttattggattttgataatgaaagatacgggggtggctggggaagaataaaatatatttctcaacatctgtatgagctgaacacagagtttcaagaatggttcaatactcagggacttgattttaatgaaattggggcacgagagtgtgaatcgatggaagataaacgtctgattaattggtttaataagctcgaagaatttaatgtctatttttcgtcgcctcttgatattgatttcttgatgctacaacattataaggaacattatctggatatgttatcttcgaaagaagggccagtcgtttcatatgctgattcaggtgggaatagtaaaaaagtaaaattaactgatttggattgtattgataaattgcagttagaagggctcgaaaaacgtatagaagaagcaaagaaagctactttaaaggataagagtggaccgggggatagttttactagtgaagagaaagaattaatgatttggtatcaatatttctttttaggtagaggaaagccaacaactcatatgcaatttttatcatctattagtaatgatgaattaaagagaaacctacctcctgtgtttaaaaaaatggttagacgtgcggaagaattacttggagatattgactatggtgaagagtgacgattggtttcctaaaggagtcattaaattagaggatgctgctttagaagtagttaaagatgttactaactgtttagtcattgctggaccaggtgctggaaaaactgaattactggctcaaaagttagactatcttttttctacaaataaatgtatttctccaaaaaagattttagctttaagttttaaaacagatgctgctgcaaatttgaaagatagagttaaaaaacgttatggtgatgaatatgcttctcgatttacgtctctaacttattcggcatttgaaaagaagattttagaccaatttagaaatgttcttcctgaggagataagaccatcgaaggactatttgattgaggaaatggaagtcataaaagaggttcttgatagaaatggattaagtactactggaatgtggaagaatagcattaaggaaattgcagaaagaattgccttgagtgagaatgattccactataaaaaatgatctgttaaagggtactcagagtaataagccggtcctcttgtataggcaaataacaaagttgagtactcaaataatagctactaacgaatacatccgtaaagcacttcaaatgacatacgattttgtatttctagatgaatttcaggatactacatatgcccagtataatttattaaaaacttgttttttaggttcgtcttgtaaattaacagcagttggagataataagcaagccattatgagatgggcaggtgcaaagcctgatatttttccaaactatattcgagactttaattcaaatgaatatcaattgtcgatgaatcaccgctctgtacctaaacttgtagaatttcaaaaagaggttcatcagatattaaatagtaatcacagttctattcagacaaataattacccagaatttcaagagggtgaaataacattatttgaatttgaaaatgaaagtatagaagctgagtcaattgcagatgatatcaaatcaaaaattcaaggaggtatacgaccatcagaaatttgtatcctagcaaaacagaagatttatgattatagttcaaaattgatagctatattaagtagcaaaggaattaaagcaagaattgaaaatgaatatcaagagctcttaaaagaccctacttgtaacctattgttagatttaatatcatgtagtcaaggaaaacgggatcctttaatttgggaaaatattagtcatttctatggaaatatcaatggaatcgatgagtttactgatgaattatctttggcaaaatcatataaggagattgatgatgtgattagtgatatcgcttccctaatttcaaattttattcccgataaagacagcatgctaaatttaattgagtatattattgaaaatataggtgatgaaagaattatttcaaacttttcaatatacaatgggaaaagtgacctaactaccattgtcaagaacttttcaaaattattatatatagagtactcccagtcacctggtttatggatagaaatagtatctaatttcaagggggagaactcaattccaatcatgactattcataagagtaaaggattagaatatgaggcagtttactttctaggcttggaggattctgcgttctggaatttcaataatcaaccagaagaagataaaagtgcgttcttcgttgcactttcacgagcaaaaagtgacctaattttcacatactgtaaattgagaaataatagggcacaaaacaatagaaatattaatgagatatattcattgctaacccagtctaacctagtaaaggttgttaattaataatgattaaggcataggaatttgttttttaaataatctaattcctatgccttatattgcatgtttgagtagttgaaaatgtgatagtagattatcaagatatacttttttgaaaactttttaataacttaattaagtttagttcagttaagtttttttcattttcttgactattatagttaggatttttttgtttcgcctttttaaagtacaaattcaaatctttttttgaaactgctagttgcttcattatctcttctgttgaaaatttactttttattaagttgtgagtcttttccaattccattttatcatcataaattactatattatttttaaatctttctaaaggatatttttcgagaccagatattgaagaaaaaactttcagcaattggtattttaaagtatttttgtataagcagtggtaataaatagtgtcagaataaccacattcaaaactttctaaatcaggggagggcactagacaatttccctgatctaaatataaagccataaagtcgataattgtaaggtgtcgataacggagttgtactaattgccaatcaacattggtttggtattgatttaaactataaggcatagtttcgaaacgatttgcatcatcgtcggtaagattaatataaaaatcgggattaggattataaataaaaaatcttccgtcttcattttcaatgaagaaccaatttttatagtcttttaaaacatgaaaatactgctctttgatatttaagtcggtatgattaaacttttgaaacaacttatttattaactcaaaaggagccgaggagtctttaggagtatttatagagccatttctagcatatattaatccttttggcaatgcagtatctttcttaggcttataatcttttgttaaaaatacaggaactctatctgtatcatgaataattaagatgtcaatttctttgttgtctatagtttcagtttgtatgctaattttaatttgattatttgttgatataaagagtttatgtagtaaatctgttaaatcttcttcatttcttctattatcatcattgtttacaccgattatatctaaagtgatattatcaattccaaaaatgatataacaatcttcgtgatgtgatgtattaacaaaattcaatatgtcacgaactaattcagactttgaatgatgccatttttgtttgaaatcatgaaactcatcttcaggagtattaagtagtaaatctatttgttctatattcagcatgcctatttctccaaatattcagtttacaggaattgtatcatagaatttaagaaagtcaattataactgtattagttgtgataacttttccaaatcatccctgctatcctaatatcattcctaagaaaatcaagttttgatttttcactgggggtttgggggcgaagccaccaagttatcttatcttatcgtaggctgtcaaaactggaagattttggttagctggcgatatgatttttggggtattgtgaccacaatgcctgagctcgcaaagaccgaacaagaagggacttaggtctttaggaagcgtactgacaatgtgaggcagccttacactgtcagacaagaataatattgaaaggatagtaggatggaacaagaaattaagttgattcgtaagcaatttagaatcacgagacaagaagaaaaacagataaaagaaatgatgagggaacaaaaactggatagtttctcagaatttcttcgtcaaaatttattgaaaaagaattatcaggatagaatttttgaaagttggttttccctttggcagtctcaaaagtttgaacaaattagtcgagatgtgcatgaagttctagttgtcgcaagagaaaatcaccaagtgactcaagaacacgtttcaatcttattgacctgcgttcaagaattgattgcggaagtcaatcaagcacagccactcagtcgtaagtttcttgaaaaatatatgggctaggagggagtaatggtttatcgctatcgtaccaatctcaaaaaagtatttttaacagattcagagttacatcaactgaatgaactgattgctaagagtcactgtcaaaatttctcagtatatgctagaaaagttctgcttaatcccaatatgtcctttgtcatgattaacactgatacctatgagcagttagtgtttgaattgagacggattggaaataacattaatcaaattgcgcgtgcgattaatcaaagccatctgatttctcaggaccagttacaagaattgagtaaaggagtcggagagttaattaaggaagtggatagagaatttcaagtggaagtgaaaagactggaggagtttcatggtagtcactaagcattttgcaactcatggtaaaaaatatcgtaggcgtctgattaagtatatcctcaatcctgataaaacgaacaatttgaaattggtatctgattttggcatgtgcaattacttagactttcctagctatgaagaaatggtagaaatgtacaatgtcaactttaccaataacgacaagttgtacgaatctagaaatgaccgacaagaaaaacatcaacagaatattcatgcccatcacctcatccaatcattttctcccgaggataatctgacacctgaagaaattaaccgcattggttatgagaccatgatggaattaacaggaggccgttttcgttttatcgtggcaactcatacagacaaaaatcatgttcataatcacatcctaatcaacgccattgatcgtaattcagataaaaaattgatatggaattatgccttagaacgaaatttacgtatgatttcagaccgcatttctaaaatggcaggggcaaaaattattgaaaagcgttactcgtatcgtgactatcaaaaatataggcagtctagtcataaatttgaattaaagcaacgtctatattttttgatgcaacagtcaaagtcctttgatgattttttagaaaaagcagagcagttacatgttcatattgattttagtcagaagcattgtcgattcatgatgacagatagatccatgacaaagccaattcgaggacgccaactcagtaaacgagatttatatgatgaagtttttttccgtacacgttttgccaagcaagagattgaaagtcgattagaatttctgttgaaccgtgttaattctttggaagagttactgacaaaagcaaaagaattgaatctaaccattgacttaaaacaaaagaatgtaacttttatcctggaagaagataatcaaaagataagtttgggtcataaaaaaataagtgataagaaattatatgatgtcaatttttttcaagattattttaaaaataaggaagtcggtgattcagaaggattagagaatttgcaggagcagtaccatgcttttcgagaagaacgagatagggacaaggtagccactgaagatattgagaaagcctttgaggaatttaaggaaaagcgagatgccgttcatgaatttgaagtggaacttgcagaacaccaaattgagaagctagtcgatcaggggatttatatcaaagtgtcttttggtattaagcagagtggtcttgtttacattcccaactatcaactggatattatggaagcagacaatctgaaaaagtataaagtttatattcgtgagacaacttcatactttgttcataacaaagaatactcggataagaatcagtacatcaaggggcgcactttgattagacagttgaccaacgatagtcagatgataccatacagaaggccaacggttgaaagtttacagaaaaaaattactgagattaaccttttgattgagttaaccgagatagataaaaagtatcaggatatcaaagacgaactagtcgtagaaatagcagagctagatattaaactgactcaaaccaatgaaaaaatcgacaccttaaacaagatggcggaagtgcttatcaaattgaagagtgatgatctgagcagtcgaaaactagcaaagtatgacttttcaaaactgaatttaacagaatcaattacattagaacacgtgagcgaagaaataagagtcttgcaagaggaactaggacattatcttgatgagtacgaaggattagctagaaggctagaaacatttgtgaaattattaaacatagggggaaaacttaatataaaaatgcaagataatatttacatatgataagattatttcataatactgggagaaattattgaaaaaaataatagtatccacaaccttctctattagtaagagttttcactgttataagttagataaatatttttttattttgagataaagtacctcatttccgtatagttttgccagtgtttttgcatcaaaatagtattctaaaatctcttctttagataaaaatttttcacaagaattagcaacaagaagaagtagagatggcatttggtacacagtattatttttcttacaaaaatctatagtatctaatgcgatctctaaagcctctattatttgaccattaatggttaggtgatgtgcataattatactttaatcgaatatagccttcaatatcttcttttaattgaaattcttttgttgaataaatttgaagaagctttttatagttttcctcgtactcatcattatgtttggtgttggagtaaaaattcgttaaggtattaagaacttttaaaaaaatgtttttgttttctgtcaaagaaccgacaagatttttcagtagacggatagctttctcctcttgattataattataaaaaagtataagagattcaatccatgttaaataatgctgttcgtcctcactaaatatttttttcttatttttttcaagattataaatatattctaggtctgtgtaattcctatgttctaaaagtttagaagacatggtaacaaagctttgtaagtcattgatggtttcaaaagagtcattgaaaaataaatttatatctacttgaagtttttttgctatcttataaagaacatcagcatgaggaatataattatttctctcaattttactaattaagctttgttcacaaatgccttcagctaagttttgctgagaaagtccaagtaatttgcgtcttgtcttaattttatcacctatggattgcatattagatctcgcttgaattatatattgataatatcgttgataattattttactctttaataagtttgttgtcaaaatatattctttattaaatagatagtaaacgaaaatatctcaaaaaagataatggcattgaaaatatctcaaataatatataaacaattgactttttccatatagtattatataattgaatcaccaataatatgaaaggggtttcattatgaaaaagacttattctataattcttttaatgttaaaactatctatagtcttactcgtcactttactcttcaataattgtataaaatcttgttcttttaaaataagtataaataacagcaaacctattgaaatatactcagaaaaaaatatcaaaatggagcgtttaggattaattgatggtaaagctgattttcgagcttaattagtaatgtttgagatattttaaaatattgaaatttttcaattgagattgtaagtaaatatatgttttttgtaaaaatttcttatattgtataaaatttgtacagttcagatgaacttgtacccattgaaagtaatttaggttggataaagaatggttaattgcatagttagtaatgctcaaacaattagagaaaagtctaagtattttaattcagacagtaaaattgattttttatttctacaaaaatggcgaaatgtgaggacattaatgacggatgagctgtttgaacttatgttaaaagaagagggctacacatatgaagagttcgcatattcattacaagaagacatagagccagtagaaaatccagaatggtataataagttcttacttattatagaaaattatgattataacaatattaattatgatgcagggattaatgttttaacattgccatttagtaagtattctttagatcatattaaggatgaaaagagagaattaactaattttagggttgatgaagaaatatttaataaattgattttacaacagagtgaagagttgtttaatttgattggtaaaactatggctttaaaattagcagaatataagagtgtgagtgatgctcaaaaaactaatgggatttttgaaaagtttttaaaagaattattttattctaaagactcttttcttcaattttttgaggagtatcctgttgtagctagaatgttaacaatccgaactcaattttttatagagaacatttgtgatttcttttataatgttaataaagatattgatgaattaaaaaatatgtttaatttatcagaaatcttaataactgatttggaattatcggttggagattctcacgagcatgggaaatctgtaattatttttgaagccaatcatagtcagaagatagtttataagcccaaaaatctaagtattgaagaaaaattatcgaaattattagagtggtttagtagtgaagatctcttagatttgcatttgccaaaaggattgtacaaggatgattatacttacaatgagtttgtggataaaaaaccatgtacaagtattgaagaggttcaaaatttttatacacgatttggttatttaatcgctttatgttatttattaggaatagatgatttacatttggagaacgttgtagctagtggtgaatatccagtaataattgatatagagacggcttttcatttatctccaaagataattccagataatatttttaataatattcttcaagaattagagcatgactctataaaaggatcatgcttacttccaagaaaaattccagttggaatgaatggggcagtggaattaagtgcattgcttggacgaggaggggagactggcagtacggtatcaactccgataaatattaacagtgatgactttagatattcagaaaagaatgtatatttttctgctggtaataatattccgatttttaatggagaagaagtagattcgaaagattttagatttaagatagttgaaggatttgaagattttttcgattttgtcttaaaatacaaaatggaactaattgagaaaattaaccagtttaaagatacaaaggtcagaattttgcttaaaggtaccgagaaatatgcagctatgcttagatattctagtcaccctaactatggaaagtgtatgaaatatagagaaagactatttctgaatatttgggcctatccatatttggataagagagttgttgtgagtgaagttcgagatttgttatttggagatataccgatattttataataaagtagggtctaaaagtttaattgatagtcaaggaagtatataccataattactttttagagtcaggattagataagtacaagaaactgatttccgttttgacaaatagggaggtagataagcaaaagaatattttattaattgagttggggctatacgatgaatatttgatttctaaaaaaggaactaaatgtagaaaattacaaaaatcaagcattaattttattcgagaagcgaagcaaattgctaattactttattgataatacaaaaaagtataaagatatgatatccatgataaatcttgattgtgacggtgaaaatcattggggtttaaaaccaatgaatgaaagtttttatagtggattaagtggggtcgcgttattctttcttgaactatataacataacaaaggagtctatttattacgattactacaagggatatatcagttctgctattttacaaacaagaaatacaacttttcaaagtccattttttggttggctttcccccttgtatcctcttcttttggagtatagatataattcaactatagttgatgaggattatttcaggtttactattaataaactaaatgcattaacaatgaaggatttatcaaatatagatagtattgattatatttctggaatttcgggagttattgttttattgcagcaaattaatttagtatacccgtataaaatatcatcaaaaactctggatctgttctacttggctcttaaagagcgattggctagggacgaggaaatagaaagcgaagctgggttagcgcatggatttgatggtgtaactgttgctatatctttaaagagagaattattagatttaaatcagaagtatcttaatattggatataagaatttacctaaattatcaaatcagtataagtggtgcttaggattatcgggaatcattcaggctaaattattgatacatcgtatagcaccatcgcttttaaacgttaataacttatatgttttatttgaaaaatttgaacaaagtatattgcatgttccaattgatgatgacagtctatgtcatggtaaggcgggagtaattattactttgtgcttgatttatgaatttactggtgaagaaaaatggaatgatatcatgatgcatcagttgcatgatttaaagttgaattctttattcggattttattcgcttcctagaattggggatacctattctcttggtttatttgatgggatggcaggaataggatggatgtacttatacttatcaaagaaatgctctaatatattgatgctcgggttctaaatagagagtatggagtagagggagatggtcaaaatggtttaattatattaaatttagtttttgcatacagtattttcaagcatagaaaggattaataatgttaaattttaaagaatcagctgtaactggagcagcgtttgaagatatgtcagttgctgaaatgagtagaattcaaggaagtggggatatttctcctgaaacaactccagcctgcgcattaataattagtaaagctatatctggtgctgcagtcagttttgctttctcagtaggggtagtaaagactgttaaaggaaattgttaggaggatacgacaatgtatgatattgatgtaaataaatatgctggtgattcatttgaggaaatgagtatagcagaaatgacaatggttcaaggatctggggatatgagcgcagaacttactcccactactccagcctgttacgcaagtattatctatactgctaaggcctcttcaccaaactgcgcatatgctgcaactgcaatttcgggtgcaatttcgggtgcaattatttcagctgtaaaatgttagcgtttgttatttaaatctaggaacaggttcgtggtatgatttcaaaattgctttggacttgttcctctttattttactataagaatggtaatttaatagtatatatattggaagtagaatttattgaatcggcatggaatttattgttccctgttacttcggttactcagattatgattttgtggtttgtgtagtcgtaaatatttaatttttgttagtttaactaaatgtctccaaaagtataacatggatggaaggaatgatttatgaagaagatttttatacttatagggagtagaaggaaaagaggaagtacatataaatttgtaaattcaatcgtcagcaatcttattgattatgagattgaatactgttttccacaagatttaaatattcggccgtgtgttggttgtcataactgttttcaaaaagctaagtgtgttttaagagatgacatctctctattagaaaataaaattctatcatcggatatctttattgtagcctctccagtatacttacactatatgtcaggagatttaaaaatgatattagatagattatcttggtgggctcatacacttagattacaaggaaaaccagttgttgttttgagtacaaatggctcaaatggggaaacatctgttacagaacctttaagtgaaataatgacagttatgggggggaatgtgattgcgaatgccaatgcttctcaatttccaaatcaaattaatgatgaatcatggatttctgaggtatcacttcagattgttgagcgaataaaacagcactctcttcactccccttgttcaaatcagtatttagagaaagcatttattgctcaaaaatataatattctacagcaaaaagaaatgagtactcttcttcctgaatatgaatttggtgaattgaaattttgggagaagactgggatgttaaattttgatacgtttgctggctatttgttgcataaattaaagcggggagttaacattgaagattaagtttcaacaacagagtgagcattctgagtgtggtcttgcatgtgtagcaatgctgattgattatttttcagaaaacatgaggctaacttatttgaggcaaagatacggtgttccaaatggaggatataattttgcccaaatgacatcaatatttaacgattataatattaaaactagatgtgttcatttgagttatgataaaattagtagtgttcccatgccgtgcattgcattctggaagaaaaagcattttgtaattattgaaagagttataagaaataaatatcatattatagatccggctataggtaaactttgtttggacgaagaagattttaagcattttttttcacaggcaattatatatatagatggaacgtatcctaaaaaaagagataacttccggatttttaagtattttttgaaaaaattaatcaagcataataaaattgttttttctgttttaagtgtttcgttcattgttcaactatcagtgttattgattccttattttattcgttcaataatagataataccccagtatggaaggaacaaggatattctgttctggttttgactttaataatgatcagtttattatattttggaactaatcatatcaaaataaaattaatagctaaacttcaaacaacaattgatagagattctataacaatagttattagtcatttattagagttgccatattcatattttactaatcgcaataaaggtgaactagtttataccttaaattcgaatacttatgttagacaagtacttattgaacaagttattgaattaattataaattttatttttgctgtactgtatttggttgcgatgttttttattaatgcaacattaacatatataactttgatattagtgatgttgataagtgtatcaattgttataaattccgtgtataataaaaaattaactcagaatgaaattatgtcactgtcgaattctcagaactatgtaaatgagattattaataatatatcgactataaaatcaacagcttcgcaaagaaatatatttaaaaaatggtcaaagaattttgaggagcaacttttctatgaggttaagcgggcaaattatagttcgatttttatgaatttttcacaaagtcttcaagtgctatatactttgataatttacgttgtagggattcaactgtctcaatcaggaacggaactgacactcggtagtattgtaggattcagttcgattggtgtgtcatttatttctccattaatttcaattctctcttcatacaatcaaatattttctattagtatttatgtaaatagattgttagatattttagatacacctgtagaagcaatggcatttggaaattatcaaattgagagcttatctggaaaaattgaagttagaaatcttagttttcgatatagtaaattttcatcagatattttcaaaaatgttacgtttagtattcatgaaagagagaagatagctattataggggagagtggttctggaaaatcaaccctactgaaactattatctggattttataaacctagtaatggattaatcctagttgaccagcaatcaatggataaatttgatttagaaagttttaggaaaaagattggtgtgattttacaagaggaccgtttgttcagtggtacacttagagataatataactttaggaagagaaatttcagatgaagaaatttggaatatcttgcttgagtcccagctagatgctttggttagtaattttccattaggattagatactattatatcagaaaatggaaataatttgtctggagggcaaagacaaaaaatatctcttataagaacacttatatccaagccatcaataatttttcttgatgaaccaacaagctctatggatgtattatctgaaaaacagatgatggatttgatttttaaactagactgcacagttgtagtcgtttcacataggatttctttagttgaaaagtttgacaagattctagtcattaaagatggaaaaattgaaggatttgattcccatgataaattaattaaagattgtcctacctacattagtctttattataaaaagtaaaggagaataaaagtgaaaagtaataatattactcaccttacaaaaaaaatgataattttaattgcagctacttgctttagtacttttccagcagttatgatttttggaaaaaaaggttttttagtttttgctgttttattttattggacattaatgacctcatttgagattgcaaaaataaaacggaaggaagatatttatactgtatcagatttattgcgagataggacaaccaatgaatataaaatgatacaatcaaatgaacgtctaaaaaactttataggtaatatgaagaaatctcaaatcattttttatggaattatttttggaatagtaatggctttaattccagttttactttatttttttattgttggtattgataaaatattgttttagtttcccaattatatatgttattgtagcgtctgaattatagaggagagtagaaaatgaattttaaaaagacattactattttgtattgttgttatgtctgcactttttgtacatactactaatatatttgcagaggatgctagtagtctatcctatatgatttccaatcaagaagcttggaagatccctgaaatttcaaaagagatggattctgaacagattacatatatacctgaaattaatcttgtgagttttcaaaatttgggtagaaaagattataatgtagtatcaaaatattttgatttagaaaaaggccactcccttcccaaaatagatataaaaaaagagattgaagtagattttattgccaatagtaaaattgaatcaatcaatgttgattcagtaagtaattctaatttagaaatcctggacattctatcagataaatttaataactttaattgggcttataagaaaatattatcaaatagaattccaactaaaaagagattaggagaaggaattaagattggaataattgattcaggtattgattttagtcaccctaaactagtaaataatgttgttaaccagaataattatgtcaacgacaatcagacagacgagttagggcatgggactcaagtagcaggtgtaattaatacgcttgctccaggagtagagatgacatcttataaagttatgggagcagacgatggagagtctttaaatgttattaaggctataattgatgctactaatgacgatatggatattattaatatcagtcttggttcttataaaaattttactaagcaggaagagaaatttacaactattgctttccttagggcaattgaatatgcacgaaggaatcaaacaatcgttgtagcttcggcagggaacgagtcgctaaatctagatacattcagtaataataacatgcatgtacctggcggacttccctcagtcattactgtagcaactactgataagagaggtaaaaaagcagattattcaaattatggttctataattagtatatcaagtcctacaggaagttttggagaacaatatatggcaactggtcaaattgatgctcgtgaaatgatgatgacttactatcccttgacaaaagattctctaattggctcattagctggatttccaaaaggatatacactttcattcggaacaagtttagcagctcctgctgtaagtgcagctgttgcagtaattttatctgaaaatagtgcacaagatagagatgttgagcatatcatattacaattaatgagaaattccgagcaatttaatagtgactcagagagcattggctacggagaattaagaattagatgagaattttatgctgttttgaagagagtctgagctatcttcttgtaatatgaggatattggtaactgcctcttgactatattgaaacgttccaaagttgatttgactctggaacgttttttgtacgacgggcatgtcgtacatctgaggtacaagtcctccatggacaccgtaatcggtaatcgcccaataagaagatatattgaaaagtcgcatagtgaaagccagcgtctttaggcgctggctggtgataggggtttatagcccttgttcaaaccaaccatttgacgggtggttatgattgctaataaagaatatatatttttagatgagtacactgttatctcctttggaatttcactgtcagagtatttggaatagggaatctataacttcagataaactaaacacaacacataaattatggtttattggatacttttctttaaagagtgatatgaatgcggcaagttaataatactacaaatatggacatttagattgctaaatattgctttaaatcttacataaagtataagtaaatgatttgataatctgctgaaaaaattattgtcatagtatgacaatttccatgatttctccgaaaagtaaaatatactttttataggagacaaatatatgtaccaacgaataagggatttgagggaagatcatgatctcactcaaaagtttgttgcaaatttactttccttttctcatgcgaactacgcaaaaattgagagaggtgaagttgttttaacggcagatgttcttgtgaagctttctaaactttatgatgttagtacagattacctattagggttaacagattgtccagataggataaaacgtaaaatgaaatagtctctccttgatttgtacattgacaattaaataaaccaatgtgggactttcttatttgtcgagcaatttagtgatatacgcgatgataggagcgataattattagctataaaatagagtggttctctatctgccatgaccacaaataaggacaatgatacttctgaacgttcaggctgccaacgtaaaaggacagcgggtgaaattcccatgattgatataaccagtcatacccacagagaataactaagagtaacatgttatcaggaggggaagttttggaacaatcagtagtaaaatatagtatgcaactttcaatgcttaactttttatattccataaatatgttgactaaaaaggaatatgaaaaaattaaaaatagactgcgtattaagtacacaaattagctgaccttttgatttgaacgcggtaatatagaatcaaaaggaggaatttcagtatgaaaaaaaataatgttgaaataataaaagctgattcgttagtacgtcgtagaggcgacaattccgaaagagacttaaaaagggttgctgcttattgccgagttagttctgatagcgaagatcagaagaacagctatgaatctcaggtgagacattataaagattatatctctcagcggtcggattgggaactggcaggtatctatgcagatgaggggatttcaggaacacaagtaggaaagagacaagattttcaacgattaataaatgattgtgtaaatggagaaatagattacattgttacaaaggcaattgctagatttgccaggaataccctagatactttgaaatatgtcagaatgttaaaagatatgcaaattggagtatattttgaggaagaaaacatagataccctaacaatggatggagaattgcttttgactattctaagctccgttgcacagcaagaagtagaaaatacatctgcacatgtgaaaaaaggattaaaaatgaagatgcaacgtggtgaattggttggctttcaagggtgcctagggtatgattatgatgtagaaaccaagcagctttcaattaataaaaaagaagccaaaattgttcgttacatttttgaaagatatctagaaggtattggaggaaaagtaatagctagggaacttgatgaacttggttacaaatcgccaagaggattagaacattggaatgatacaacagtcctaggaataataaagaatgaaaaatataaaggtgatattctgatgggaaaaacttttacagttgacccaataagtaagagaaggttgactaattttggagaagaagataagtactatatcaaagataatcatgaacctataatatctaaagaagattttgaaaaagctcaggagattagactgcgtcgagcaggaaataagaagactgctgcaaatgtaaatggcaagcgtgaacgctattcaaaaatgtatgcttttagtagcatgttagaatgtggtttttgtggttcaatattatctagaagaagttggcactgccgttcaatattatctagaagaagttggcactgccgttcagattatcgcaaggttgtgtggcactgtgttacatcgataaagaaaggaaagaaattttgtaagcatagtaaaggattagaagagctcgctattgagggggctttcatggaagcttacagacaactttatcattcaaatgaaaacttaatgacagacttacttgaaacgattgaatctgaattgaatgacaatagccttaacaaggaattaaaaaggattacaaacaaacttcgaacattactaaaaaaagaggaaaatcttgtaaatctaaggctcgaagggaaaattagcgataccatatacaatgaaaagtacaatgaaatttcctcagaaaaagagtttctagcagaagagaaggtaaatattgaaacaaccttaaaatcagaaatcgatgtaaagaaaagactaactgagtttaagcacttgctatcttcacagaaaatgcttacagagtttgatcgtgcagtttttgaaagtattgttgagaaaattattgttggtggagttaatagcgacggagaaattgatcctgcaatgttaaccataatattcaaaacaggtgaaactcaaaacaaggatggaaaacaattcaaaagtaaacgtaaaaatgctaaactagaaacagataaattgtgtcctcaaaacagtgacgaggataaaaaaatgtattctcaaggaccagacaacacatgttgaagttgtgagtctgttagaaaaacgcaattagattgtccagcttactctcttgttttctggcaagctgcttcaaaggtccgcaggacctttgaactcccaaagagaatggcacttcaaaaataaaatgaatgaggggaaagttgaagaatttgacggtatctctttgattgtttggacagccgataataagataaaagcgttgaaagagtttggttgtaattgtaacaactacaatccttacaaagagagtgaaacaccattatttagagatgaaaaagtaaattggttttgaggagataaaatattgatggaaagtagacctgagtttgacaaaattacatcgtttgatgaatttactaaatactattggtatcgtgaagaactttcacagatatgcaagtcattaggattagaatatagaggcacaaaacaggaactcaattatattattgagcaatactttaagggcaatttgattaaaaaatcatcaataaaaaatgaaaagaagcaagtggaaaatattactttagatacaccattacttgaatgtggtttttcttttaatgcaaagttcagagaatatttcactgttttaacaggtatctcaccatttaaatttactgctgatatggcaacagcttggaggaaagtaaaaagagaaaaggatttgagttttacaattcaagatatgctaaaagtttattatggaaaatcaaattatgctaagtatgacaattcggtttgtcaatggaatcaatttttaaaggatttctgtgcagacgaaaatagttgcaactactcaaacaaattaaaagtagcttctattctttggaaagaagtcagaaattcaagaaatgaaaaaatttattcaaagaatcttttgactgaatatgccgataaaataaaagagtattgcaagtaggatatttttagcttgctaaactaacaattcgataaaaactaacacatgaacagtaaatcaaaaagtgaacagatgttggaattttttggtaagtattcaatgtcaatgattttaataggaatagtcttagtaggaatcattattataggaattgcaataaatttatctattggacttgttcggtaactacatcatctcgtggttgatgatgccacaaatcaactcagctcttaattcaaaccgttttctacgattacgatagggttgtgccatgatttgaaaggttttaaatttggcattaaagtgctcgacttcaatacggattttagccatctctcgattcaaccgtttatcctcttcggttaagaggtgatgtttagaacgcttagcaggaataaaggtattttcgtgaaatttcaagatgcctaaataccctaaatctacgaaaacaagggtattttggggcaaactatagccaatactctccttaaaaagggtaaaatcatgcgtatgatcatctgaaaaagccaattgacaagcacgatggctagtcaaatcaagcatgatttgagtttttatggtatgtctttttttaccagaataattcttgctttggttttttagggcgttgaataggactttcagtgacatcaatggcaacagtggcgctaggtgcttctaagtggtctaaatcaaagagacctgaagaacgaagggggtcctctacccatgtaatggtctcat

## Supplementary Note 2

### English translation of Dutch Encyclopedia pages

The encyclopedia entries were translated using Google Translate and subsequently manually curated.

Source: <http://encyclopedievanzeeland.nl/Varkens>

### Pigs

Text from the Encyclopedia of Zeeland. 1982-1984

Pig farming at Zeeland was traditionally largely limited to the house slaughter of pigs for personal use by farmers and farm workers. In the 19th century, only four to eight pigs were kept on big farms. Nevertheless, the number of pigs between 1850 and 1900 increased from 15,000 to 30,000. Fattened pigs were usually slaughtered in November at approximately 200 kg live weight. It was mostly the Flemish land pig, which grew fairly slowly but were resistant to be firstly held as a walking pig on the dung heap and then to be fattened with barley flour, possibly supplemented with pea flour, buttermilk and boiled potatoes (seed potatoes). The slaughter was accompanied with some ceremony, on the day of slaughter family members and neighbors were treated to meat. Little was done to improve the pig.

**Around 1860, I.G.J. van den Bosch, director of the Wilhelmina polder, imported Great Yorkshire pigs, which, both in pure breeding and crossing with the Flemish land pig, spread across the Zeeland islands. In Zeeuws Vlaaderen, however, they remained faithful to the old land pig. Especially on sandy soils where more piglets were bred there than were needed for their own supply: the surplus, known as the Cadzandse piglets, continued their way into the provinces.** Still in 1937, the whole Zeeland pig production was mainly intended for their own use. Over 24,000 pig farmers kept around 31,000 fattened pigs and bred with 4,000 sows approximately 48,000 piglets. In that year there were 63 mutual pig insurances (pigs funds) in Zeeland, which represented approximately 6,000 pig farmers.

Breeding: In 1915 the provincial commission was founded for improvement of the pig in Zeeland. Registered were the Flemish bred land pig and Large Yorkshire pig. In 1932, 83 sows and 38 boars of the refined Danish land pig were imported. In 1936, virtually all breeding boars in Zeeland were of the Danish type: therefore the provincial commission closed the studbook for the Flemish land pig. However, the breeders in Zeeuws Vlaanderen clung to the Flemish land pig and therefore initially hampered the expansion of the Danish type which is better and since 1946 called the Dutch land pig. The Zeeland studbook for pigs was discontinued in 1972. Since then, the Zeeland breeders are affiliated with the Southern Dutch studbook in St. Oedenrode, which is present in Zeeland, Noord Brabant and Limburg.

Stables: After 1960 in Zeeland modern fattening breeding farms were built with units of at least 200 pigs and 30-50 breeding sows. Fattening pigs are kept in flocks of 8-10 animals per cage with straw as bedding. Fertilization and feeding occurs increasingly through mechanical systems. Even newer is the slatted floor shed, in which the pigs trample manure in the gaps between concrete beams to a manure pit located below. Since no bedding can be supplied, these stalls are heated. During the last years pig bedding and fattening is concentrated on fewer companies, but with a greater number of animals per farm. The company average is in Zeeland is still behind in development compared to the rest of the Netherlands. Modern pig farming requires an entirely new approach regarding the relationship breeder-fattener (pigs healthcare, accommodations, feed (by factory-prepared compounds)). The familiar house slaughter pig is doomed to disappear.

Authors: Den Engelsen, M.A. Geuze

Source: <http://www.encyclopediedrenthe.nl/Varken/varkenshouderij>

### Pig/Pig farming

Since the mid-19th century pig farming became to one of the three pillars of the mixed company on sandy soils, the so-called: Étagebedrijf. Along with this development the habitat of the animal radically transformed in a short period.

Before that the original land species could still be found in Drenthe, which were referred to by names such as 'steep-ears' and 'smooth-ears'. The steep ears (also called 'staggen') were the smallest; it was a pig with little steep, short stout legs, a straight back and a thin tail. When they were ready for slaughter at the end of the manure season they weighed 180 around the (old) pounds. Both species were in fact much like a wild boar. Smooth-ears or ‘loboren' were heavier at slaughter and weighed around 225 (old) pounds. Drenthe seems to have been held a third species as well, called ‘cross-ears’ which stood in between the first two species .

**After 1850 these ancient land species disappeared rapidly to make room for crosses with foreign, i.e. English, species. The provincial report on 1853 reported: "*The breeding ... of pigs of English breed, crossed with home grown, is expanding more and more, and provides continuously good income*”. Initially, the ‘English breeds' were thought to be exclusively pigs of the Berkshire breed, which as a type was considered more a bacon type of pig instead of a meat type of pig. From 1865, however, Berkshire crossings occurred less and less, since they gave way to the crossing of the ancient indigenous varieties with Yorkshire pigs. The reason was a growing interest on the export markets, particularly the UK, for more proportionality between bacon and meat.**

Until about 1890 the Drenthe farmers sold their fat pigs to merchants who then exported them alive. When under pressure from a deteriorating economy the exporting countries (England and Germany), closed their borders to live cattle, farmers were forced to switch to the fattening of an entirely different, lighter type of pig though which they could focus specifically on demand for fresh pig meat originating from England. This special market required a type of pig that only needed to be fattened to a weight of about 50 kg and these pigs were soon referred to as London's piglets. It was a form of pig farming which was ideally suited for the small farmers and agricultural laborers business because the money invested could be recouped quickly.

Together with the rise of the cooperative dairying (see: dairy industry), this form of fattening have therefore had a major influence on the rise of small farms on sandy soil. Noteworthy is that farmers in some Southwest Drenthe municipalities like De Wijk, Ruinen, Ruinerwold, Havelte, Diever and Dwingeloo had already focused since the late 18th century on the breeding of 6-week piglets which, with the rise coming from milk production, in the spring were sold to grazing lands elsewhere in the country. Later, that is, after about 1890, it was assumed to be more or less pure-Yorkshire boars with the newer types of native pigs. With the increasing demand for six-week piglets – partly resulting from the rise of the fattening London’s piglets - piglet breeding became more and more omnipresent here, with Meppel at this point developing into a major trading center. The result was therefore that the supply of six-week old piglets at the Meppel market between 1870 and 1910 increased from about 9,000 up to about 50,000 per year.

When England in 1926 suddenly closed its borders for import of fresh pork, farmers on the sandy grounds saw themselves forced to switch to fattening of heavier, so-called bacon pigs or ‘salters’ where neither they nor the processing export abattoirs had experience with. Moreover, this market had to fear the competition of the Danes who had previously specialized under similar conditions to this type of fattening and in that area had a lead now. The requirement type for the preparation of bacon was a long and fleshy pig, with an even, not too thick strip of back fat, which were delivered with a weight of 90-96 kg. To develop a proper bacon type they went on to import the Danish breeding stock. By systematic crossing of animals of the Danish Landrace with the pigs here they succeeded in a short-time to improve the quality of the Dutch bacon pigs significantly. This new breed was called the Dutch land pig (NL) and was soon to be found anywhere in the sandy areas. The government stimulated this whole process in the framework of the agricultural crisis measures in the 30s, by focusing on the quality improvement of pig farming and by paying the farmers through the specially constituted so-called Pigs Central strictly according to the quality of delivered pigs.

The significance of the pig for étagebedrijf was evidenced by the fact that it was counted at the beginning of the 50s that the income from this type of business was derived on average for around a fifth of the total. As a result of the changes in the farm on the sandy grounds, pigs disappeared from many companies in Drenthe when the farmer focused on the dairy industry. The number of pig farmers in Drenthe decreased from over 15,000 in the 50s to only a few hundred now.

Author: Bieleman
